# Assessing the Impact of Timing and Coverage of United States COVID-19 Vaccination Campaigns: A Multi-Model Approach

**DOI:** 10.64898/2026.04.07.26349269

**Published:** 2026-04-08

**Authors:** Anjalika Nande, Soren L Larsen, James Turtle, Jessica T. Davis, Shraddha Ramdas Bandekar, Bryan Lewis, Shi Chen, Lucie Contamin, Sung-mok Jung, Emily Howerton, Katriona Shea, Clara Bay, Michal Ben-Nun, Kaiming Bi, Anass Bouchnita, Jiangzhuo Chen, Matteo Chinazzi, Spencer J. Fox, Alison L. Hill, Harry Hochheiser, Joseph C. Lemaitre, Sara L. Loo, Madhav Marathe, Lauren Ancel Meyers, Carl A. B. Pearson, Przemyslaw Porebski, Emily Przykucki, Claire P. Smith, Srinivasan Venkatramanan, Alessandro Vespignani, Timothy C. Willard, Katie Yan, Cecile Viboud, Justin Lessler, Shaun Truelove

**Affiliations:** 1 Johns Hopkins University, Baltimore, Maryland, USA; 2 University of Oxford, Oxford, UK; 3 University of Illinois, Urbana Champaign, Urbana, Illinois, USA; 4 University of California, Berkeley, Berkeley, California, USA; 5 Predictive Science Inc., San Diego, California, USA; 6 Northeastern University, Boston, Massachusetts, USA; 7 University of Texas at Austin, Austin, Texas, USA; 8 University of Virginia, Charlottesville, Virginia, USA; 9 University of North Carolina at Charlotte, Charlotte, North Carolina, USA; 10 University of Pittsburgh, Pittsburgh, Pennsylvania, USA; 11 National University of Singapore, Singapore, Singapore; 12 Princeton University, Princeton, New Jersey, USA; 13 The Pennsylvania State University, University Park, Pennsylvania, USA; 14 The University of Texas Health Science Center at Houston, Houston, Texas, USA; 15 University of Texas at El Paso, El Paso, Texas, USA; 16 Northern Arizona University, Flagstaff, Arizona, USA; 17 University of North Carolina at Chapel Hill, Chapel Hill, North Carolina, USA; 18 National Institutes of Health Fogarty International Center, Bethesda, Maryland, USA

**Keywords:** ‘COVID-19’, ‘Scenario modeling’, ‘Ensemble projections’, ‘Vaccination timing and coverage strategies’, ‘Seasonal dynamics of respiratory infections’, ‘Mathematical modeling’, ‘Multi-model aggregation’

## Abstract

Six years after its emergence, SARS-CoV-2 continues to have a substantial burden, however, the impact of vaccination and the optimal timing of its rollout remain uncertain. To explore these uncertainties, the US Scenario Modeling Hub convened its 19th round of ensemble projections for COVID-19 hospitalizations and deaths in the United States. Eight teams provided outcomes for each US state and nationally from April 2025 to April 2026 under five scenarios regarding vaccine recommendations and timing. We assessed recommendations with two eligibility scenarios (high-risk individuals only and all-eligible) and two timing scenarios (classic start: mid-August, earlier start: late June). These were crossed to create four scenarios and were compared against a counterfactual scenario with no vaccination. We found that compared to no vaccination, our ensemble projections estimated 90,000 (95% PI 53,000–126,000) hospitalizations averted in the high-risk and classic timing scenario across the US. Expanding coverage averted an additional 26,000 (95% PI 14,000–39,000) hospitalizations, which when coupled with earlier vaccination timing further reduced national hospitalizations by 15,000 (95% PI −3,000–33,000). These findings estimate significant benefits from a broad all-eligible vaccination recommendation, and suggest an additional benefit is likely to be gained from an earlier vaccination campaign.

## Introduction

Six years after the first reported case of COVID-19 in the United States,^1^ SARS-CoV-2 remains a substantial source of disease burden, with an estimated 390,000 to 550,000 hospitalizations due to COVID-19 in the US 2024–25 respiratory season.^2^ During this period, policy questions have evolved alongside disease dynamics and variants of concern,^3^ and in particular, the impact of vaccination campaigns at the population level has been an area of continued exploration.^4,5,6^ Substantial heterogeneity across age in transmission^7^ and risk of severe disease ^8^ has necessitated evaluation of vaccine prioritization and eligibility, for example in the context of resource limitations when vaccines were first approved,^9^ and later on, expansion of vaccine approval to younger age groups.^10^ Several factors have the potential to influence COVID-19 booster impacts – and consequently, vaccine policy recommendations – in future years. These include that the majority of the US population is no longer immunologically naive,^11^ the propensity to get vaccinated has decreased, the increased prevalence of hybrid immunity^11,12^ may decrease the rate of immune waning,^13^ and the landscape of variants continues to evolve^14^. Further, for the past several years, both a summer and winter wave have been observed, in contrast to influenza and RSV dynamics, which only show a winter season wave.^15^ In light of these considerations, the population impacts of vaccine campaigns in 2025–26, and subsequent seasons remains uncertain. To navigate these uncertainties and support evidence-based policy decisions, scenario modelling can compare alternative futures and enable projections informing vaccine policy decisions under various epidemiological and immunological assumptions.

Since December 2020, the US Scenario Modeling Hub (SMH) has generated 18 rounds of ensemble-based projections at the state and national level to inform COVID-19 policy.^16,17^ These projections are ensembled by aggregating outcomes from multiple mathematical models, solicited in an open call.^18^ This approach has been shown to enhance the reliability of predictions, compared to those from individual models.^19^ For each round, SMH defined a set of scenarios in collaboration with decision makers, aimed at answering specific questions for policy or about critical epidemiological uncertainty; these have included vaccine effectiveness, vaccination coverage, timing of vaccine rollout, waning immunity, emergence and characteristics of new variants, and non-pharmaceutical interventions.

This study presents the results from SMH’s 19th round of COVID-19 projections conducted in early 2025, examining five scenarios (A-E) across a 52-week period for the 2025–26 season (April 27, 2025 to April 25, 2026) that vary vaccination campaign timing and target population coverage (Table 1). Unlike seasonal influenza and RSV, since 2022, seasons of COVID-19 in the US have been characterized by two annual waves, making vaccination campaign planning and evaluation challenging. While winter peaks remain common, substantial summer activity has been observed in the last three years, potentially driven by a combination of climate factors, behavioral responses, waning immunity, and changes in susceptibility driven by viral evolution.^20,21, 22^ To account for this dual-wave pattern, SMH evaluated the potential impact of starting the vaccination campaign 1.5 months earlier than in previous years, alongside different coverage recommendations, on projected COVID-19 hospitalizations and deaths. These scenario projections are not intended as forecasts or to predict exact epidemic target outcomes, but rather to explore plausible epidemic trajectories under alternative vaccination strategies.

## Results

### Projected national-level burden of hospitalizations and deaths

**Figure 1.**
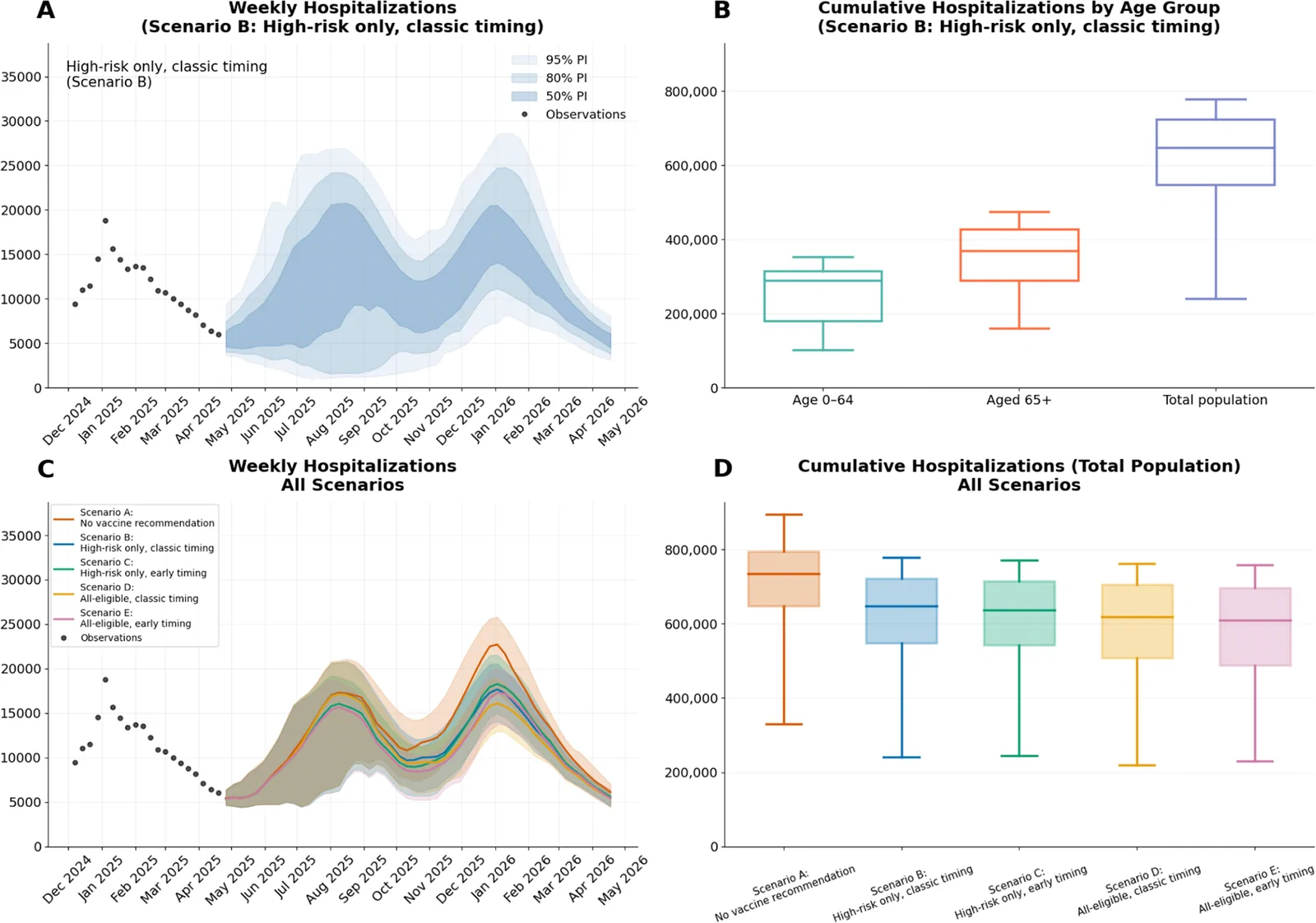
National-level ensemble predictions of COVID-19 hospitalizations in the US, 27 April 2025–25 April 2026. A) Projected incident hospitalizations across Scenario B (high risk group vaccination and classic timing). Shaded regions represent the 50%, 80%, 95% projection intervals. B) Projected cumulative hospitalizations by age-group and total population of Scenario B . The outer boxes represent the IQR and the whiskers are the 95% projection interval. C) Projected incidence of hospitalizations across all scenarios. Shaded regions represent the 50% projection interval and the solid line represents the median value. D) Projected cumulative hospitalizations for all scenarios . The outer boxes represent the IQR and the whiskers are the 95% projection interval.

The national-level hospitalization ensemble of eight models projected two distinct periods of increased COVID-19 activity: a summer wave peaking in August 2025 and a winter wave peaking in January 2026 (Figure 1A), with similar timings projected for peak deaths (Figure S2). Based on the 50% projection interval, both peaks were expected to approach those observed during the previous winter season (2024–2025), peaking at approximately 20,000 weekly hospitalizations nationally. While projections for the winter wave were relatively consistent across models, uncertainty around the summer wave size was greater, with two of eight participating models projecting insubstantial summer activity (Figure S3). At the national level, there is limited variance across scenarios. Peak timing is largely consistent, however the peak magnitude and cumulative burden varies slightly depending on the vaccine schedule and coverage (Figure 1C,D). State level projections were more heterogeneous, with some states projected to experience much larger winter waves (e.g., California, Hawaii) and others to have larger summer waves (e.g., Pennsylvania, Ohio) (Figure S4).

Projections indicated substantial continuing COVID-19 hospitalization and death burdens, with an estimated 648,000 cumulative hospitalizations (95% prediction interval [PI] 241,000–778,000) and 40,000 deaths (95% PI 25,000–59,000) during the 2025–2026 season under Scenario B (vaccination recommended for high-risk individuals only, with classic timing consistent with the previous season; Figures 1B, S2F). The majority of the burden was projected among adults aged 65 years and older, accounting for 57% of hospitalizations and 81% of deaths.

### Impact of vaccination coverage and timing

**Figure 2.**
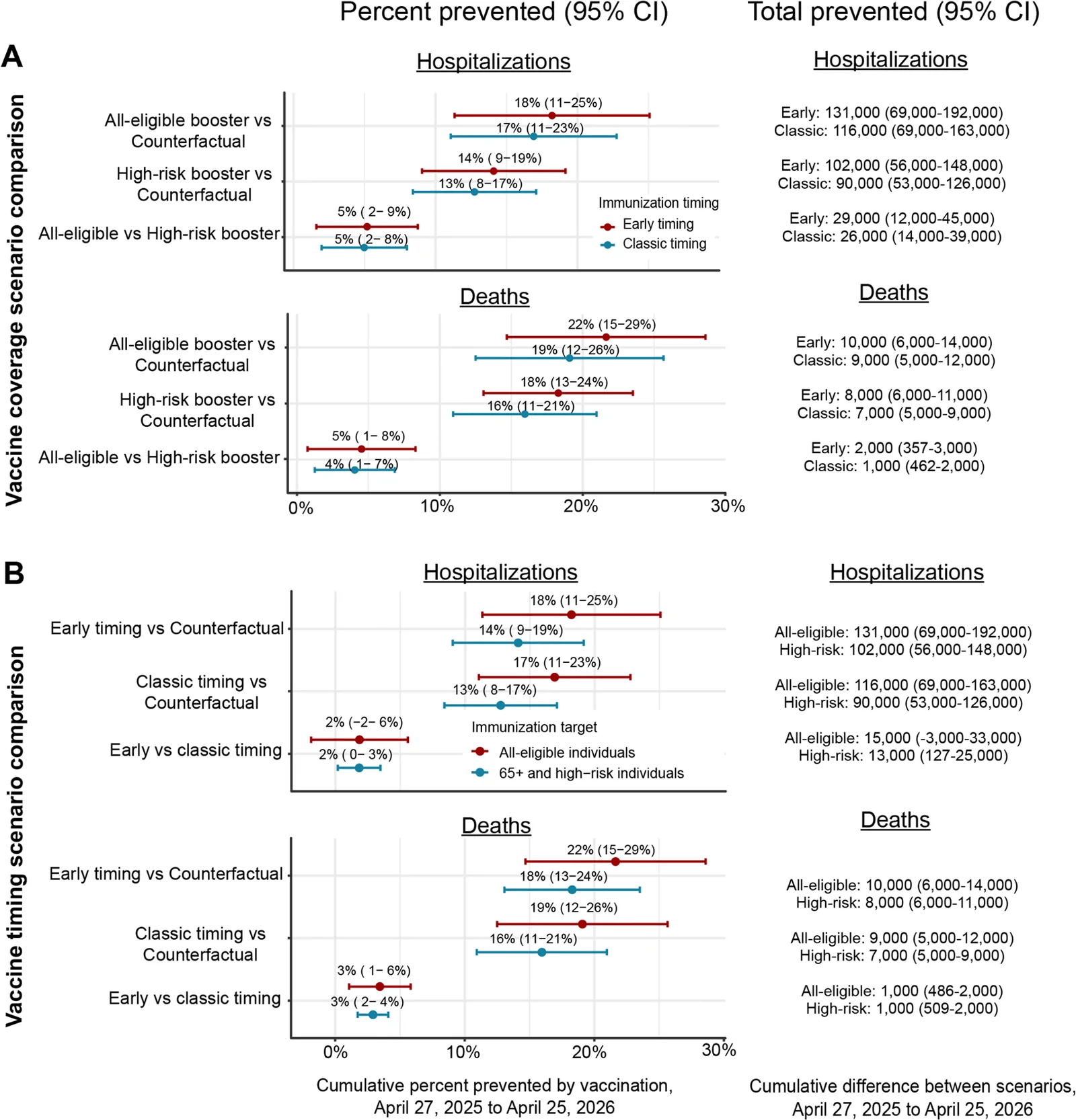
Projected national-level impacts of COVID-19 vaccination recommendation and timing scenarios in the United States, 27 April 2025 to 25 April 2026. A) Projected percentages and values of hospitalizations and deaths averted due to either all-eligible or high-risk only COVID-19 booster vaccination recommendations, under either early or classic timing assumptions. B) Projected percentages and values of hospitalizations and deaths averted due to early (starting 29 June) or classic (starting 15 Aug) COVID-19 vaccination timing, across the projection period among all age-groups, nationally. These projections suggest significant value of continued COVID-19 booster vaccinations, for both high-risk and all-eligible recommendations, as well as potentially significant impacts of shifting the vaccination campaign earlier.

Across both national and state levels, cumulative pooled results showed that vaccination recommendations, whether limited to high-risk groups or extended to all eligible individuals, were projected to substantially reduce hospitalizations and deaths compared to the counterfactual Scenario A with no vaccination recommendation, regardless of campaign timing (Figure 2A). Relative to Scenario A, recommending vaccination for high-risk groups (Scenario B) was projected to reduce cumulative national hospitalizations by 13% (95% confidence interval [CI] 8–17%) and deaths by 16% (95% CI 11–21%) over the projection period with classic vaccination timing. This corresponds to approximately 90,000 (95% CI 53,000–126,000) fewer hospitalizations and 7,000 (95% CI 5,000–9,000) fewer deaths nationally.

Extending vaccination recommendations to all eligible groups (Scenario D) was projected to avert about 116,000 (95% CI 69,000–163,000) hospitalizations and 9,000 (95% CI 5,000–12,000) deaths, preventing an additional 26,000 hospitalizations and 2,000 deaths compared with the high-risk–only recommendation. Advancing the start of vaccination campaigns by 1.5 months (Scenarios C and E) was projected to additionally reduce hospitalizations by 2% (95% CI −2–6%) and deaths by 3% (95% CI 1–6%) relative to the classic timing (Figure 2B).

Most of the vaccination benefits were projected to occur among adults aged ≥65 years, the result of both direct reductions in outcomes and indirect effects through decreased transmission. In this group, Scenario B was projected to avert 67,000 (95% CI 38,000–96,000) hospitalizations and 6,000 (95% CI 4,000–7,000) deaths compared with Scenario A (Figure S5). Despite equal vaccine coverage among those aged ≥65 years across scenarios, all-eligible vaccination recommendations (Scenario D) were projected to avert an additional 12,000 (95% CI 6,000–18,000) hospitalizations and 1,000 (95% CI 395–2,000) deaths among this group, reflecting significant indirect benefits of vaccinating the full population. While these patterns are consistent across models, individual model estimates varied considerably, reflecting differences in model assumptions on seasonality, vaccine effectiveness, and waning immunity (Figures S6–S7).

### Relationship between vaccination timing, epidemic timing and vaccination benefits

**Figure 3.**
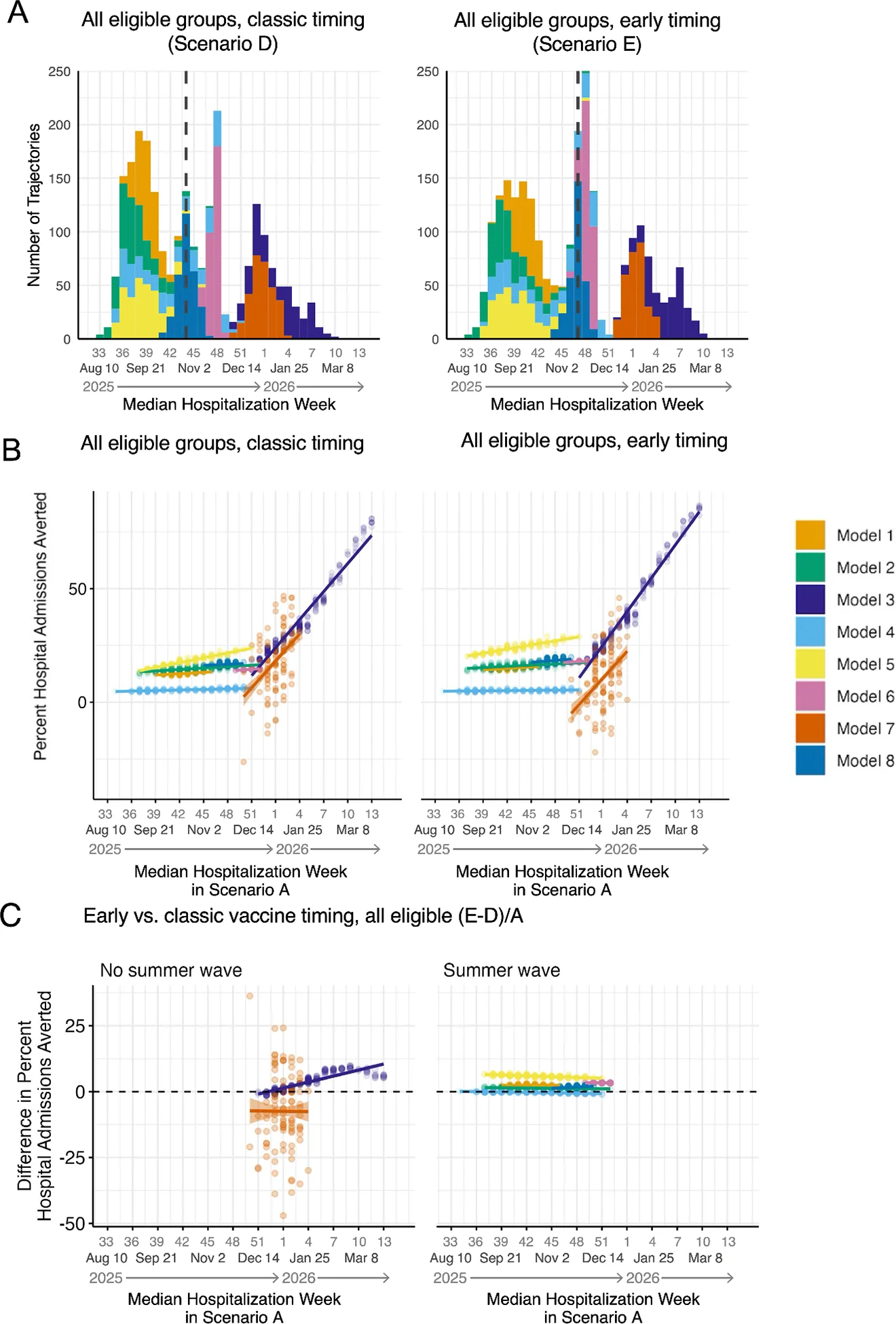
Relationship between projected COVID-19 trajectory timing, vaccination timing, and estimated vaccine benefits, 27 April 2025 to 25 April 2026, United States. A) Distribution of the timing of COVID-19 hospitalizations for Scenario D (left) and Scenario E (right), as characterized by the median hospitalization week (MHW), and colored by model. The median MHW across all models is indicated by a vertical dashed line. B) Percent hospitalizations averted in Scenarios D (all eligible, classic vaccine timing) and E (all eligible, earlier vaccine timing), relative to the counterfactual Scenario A, vs. trajectory mean hospitalization week (MHW) in Scenario A. C) The difference in percent averted between early timing (Scenario E) and classic timing (Scenario D) vs trajectory MHW, calculated as $\frac{E-A}{A}-\frac{D-A}{A}=\frac{E-D}{A}$. Models have been grouped into those with a summer wave (right subpanel), and those without (left subpanel). Solid lines correspond to results of linear regression; coefficients are provided in Tables S5–S10. Dashed lines denote the threshold above (below) which early (classic) timing is better. Note: under classic vaccine timing, at least 50% of the final vaccination coverage has been attained for all age groups by epidemic week 46.

Projected national-level epidemic timing of individual model trajectories was characterized by the week at which 50% of hospitalizations were reached—which we refer to as the median hospitalization week (MHW; Figure 3A). Focusing on the all-eligible vaccination coverage and classic timing scenario (Scenario D), the majority of MHWs fell earlier than EW 43 (week of 2025–10-19) for four out of eight models, while two models exhibited trajectory timings concentrated between EW 44–51, and the densities for the remaining two models were concentrated during EW 50 and later. This heterogeneity persisted across all scenarios (Figure S8), therefore all timing interaction analyses were broken out by model—rather than taking results from the ensemble as a whole. In addition to MHW, trajectory timings were classified by which wave was larger: summer, winter, or similar (see Supplementary Results 2A and Figures S9, S10).

Earlier vaccination timing relative to epidemic timing has the potential to reduce COVID-19 burden, but the actual impacts of a 1.5-months earlier start time were not conclusive. First, when regressing across all paired trajectories and models, we observed a consistent positive relationship between later trajectory timing and greater relative vaccine impacts, regardless of scenario (Figure S11). Later trajectory timing also appeared to increase the relative effect of 1.5-months earlier vaccine timing overall, though this association was nonmonotonic (Figure S12). However, because each model covers only a portion of the MHW space (Figure 3A), it was challenging to analyze the interaction of epidemic timing and vaccination timing in the ensemble. To address this issue, within-model timing trends were analyzed. When looking at continuous trajectory timings at national (Figure 3B) and sub-national (Figures S13,14) levels, the percent hospitalizations averted was consistently higher with later epidemic timing across all models and scenarios.. Comparing the percent averted by early versus classic vaccination timing in scenarios with the same vaccination recommendation groups (C and B, D and E), earlier vaccination timing averted more hospitalizations regardless of trajectory timing for six out of eight models across national-level (Figure 3C) trajectories and five out of eight models across state-level (Figures S15,16) trajectories. However, models do not agree on how trajectory timing affects the relative hospitalizations averted (in the classic timing versus early timing scenarios). There was a consistent positive association in two out of eight models across national trajectories, and three out of eight models across state-level trajectories. This means that later epidemic timing would increase the advantage of early over classic vaccine timing in these cases. Subnationally, the percent of hospitalizations averted was positively associated with MHW across 98.2% of location, model, and scenario combinations (Figure S17). In 66.2% of location-model combinations, later epidemic timing was associated with reduced effectiveness of early vaccination against classic timing scenarios, but the strength and direction of this association was largely model dependent (Figure S18). Note, these trends were largely preserved when considering a discrete characterization of trajectory timings (summer-dominant, winter-dominant, and similar) instead (Suppl. Results, Figures S19–S21).

### Effects of vaccination scenario on summer and winter waves

**Figure 4.**
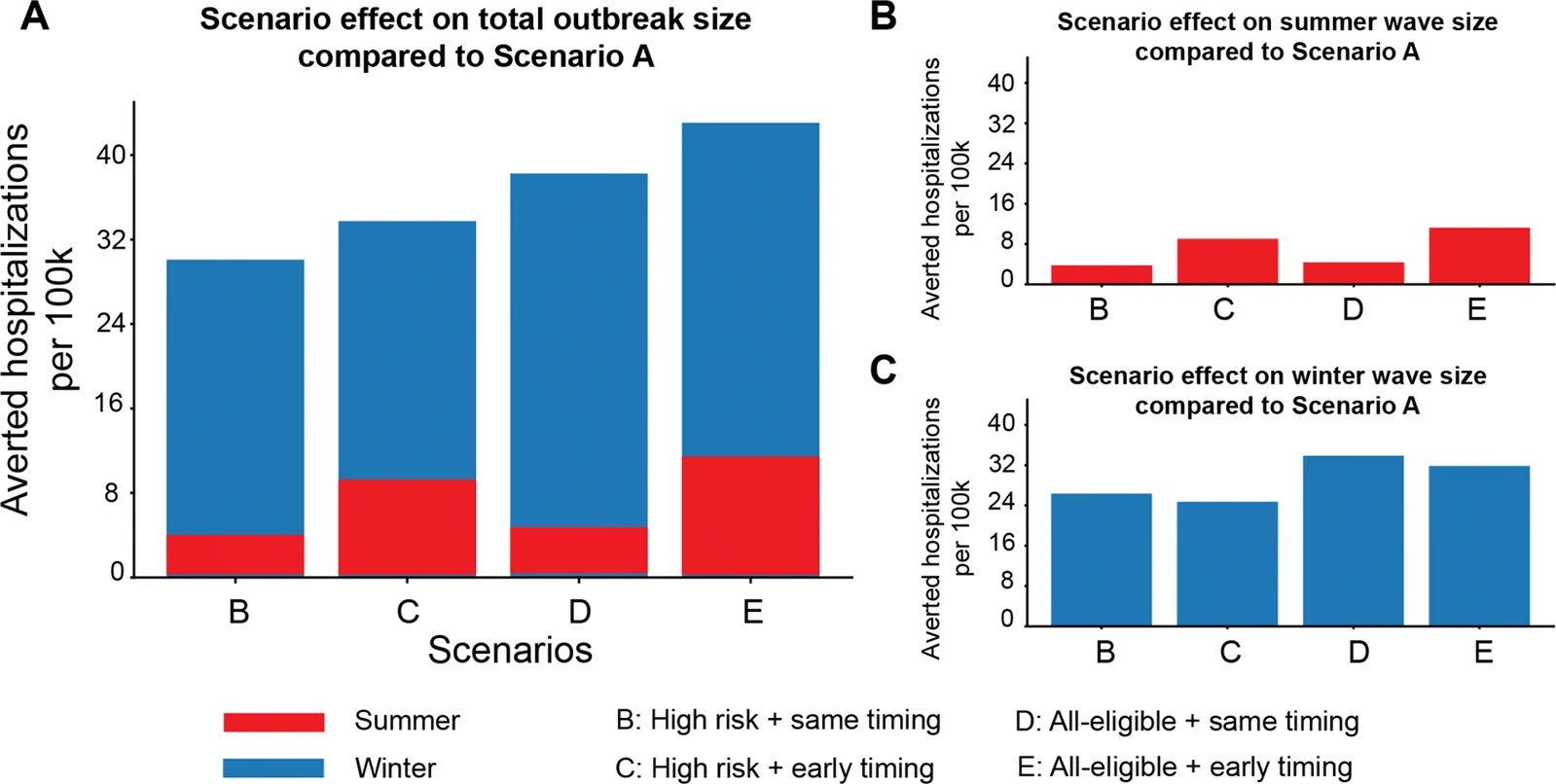
Averted COVID-19 hospitalizations under the vaccination scenarios compared to the no vaccination Scenario A, 27 April 2025 to 25 April 2026, United States. Mean vaccination effect, defined as the number of hospitalizations averted per 100,000, on the A) total, B) summer and C) winter wave sizes for the ensemble model with scenarios as the fixed effect and states and teams as the random effects. The effect on the total outbreak is divided into effects on the summer (red) and winter waves (blue). Comparison between scenarios indicates that vaccination timing has a larger effect on the summer wave, whereas vaccination coverage has a larger effect on the winter wave. Across all scenarios, vaccination has a larger effect on the winter wave compared to the summer wave.

We investigated the effect of the vaccination scenarios on the summer and winter wave sizes in the ensemble (Figure 4) and the association between them for each model and location (Supplementary Methods 1D, Results 2C, Figure S22). Comparing the two scenario axes, vaccination timing had a larger effect on the summer wave size compared to vaccination coverage level (Figure 4B): earlier vaccination averted 5.30 (per 100,000) more summer hospitalizations (Scenario C vs B) on average, whereas increased vaccination coverage (Scenario D vs B) averted only 0.63 (per 100,000) more hospitalizations. In contrast, vaccination coverage had a greater impact on the winter wave size (Figure 4C): increased vaccination averted 7.52 more winter hospitalizations per 100,000 (Scenario D vs B) on average, while earlier vaccination increased winter hospitalizations by 1.64 per 100,000, on average (Scenario C vs B). Overall, expanded eligibility and earlier timing increased averted hospitalizations by 12.95 per 100,000, on average (Scenario E vs B) over the entire outbreak (Figure 4A). Full results of the analysis are provided in Table S11. These trends were maintained when the analysis was repeated for each model individually, separating out locations by size of the summer and winter waves, or by historical vaccination coverage levels (Figures S23–S26, Tables S12–S22 and Supplementary Results 2D).

## Discussion

In the nineteenth round of US Scenario Modeling Hub COVID-19 projections, we found the need and value for continued vaccination to curb COVID-19 burdens (hospitalizations and deaths) during the 2025–2026 season. Further, we found novel results that indicate earlier vaccination timing is likely to provide additional population-level benefit. Specifically, vaccination recommendations for all eligible groups and an earlier vaccination campaign combination was the most impactful in averting COVID-19 hospitalizations (18%, 95% CI: 11–25%) and deaths (22%, 95% CI: 15–29%), compared to the counterfactual scenario without vaccination recommendations. While these effects varied substantially across models, the diversity of model structures, approaches, and assumptions allowed us to further explore questions about vaccine timing, epidemic timing, and the drivers of summer vs. winter wave sizes.

While segregation of epidemic timings across individual models hindered ensemble analysis of the relationship between epidemic timing and vaccine impacts, analysis of within-model trends yielded multiple key insights. First, there were overwhelmingly positive associations between later MHW and higher percent of averted hospitalizations across individual models, scenarios, and spatial scales, suggesting strong evidence of modest benefits from earlier vaccination relative to epidemic timing, presumably because more individuals were immunized by the time of high epidemic activity. While not directly observed, there is likely a point of diminishing returns when an early vaccination campaign (i.e., 2.5-mo earlier than classic timing) is too early. Second, early vaccination timing generally reduced COVID-19 burden more than classic vaccination timing, but the relationship between the scale of these impacts and the timing of the epidemic was inconsistent and inconclusive. Moreover, we find optimal vaccination timing may fall outside the range of the discrete vaccine timing scenarios, indicated by the larger trend line slope for models with greater period between vaccination and epidemic wave (i.e., Figure 3C). This suggests the need for a more granular and broader vaccination timing axis to effectively capture this optimum at both national and state levels. A follow up study with greater vaccination timing variability, including in the context of the immune landscape and waning of immunity, could further explore this tradeoff, and provide more granular evidence to support optimal timing of vaccination campaigns at the state level.

Despite model variabilities, effects of vaccination scenarios on the summer and winter wave sizes were qualitatively similar across individual models, with more averted hospitalizations in states dominated by winter waves. Additionally, we found that the summer wave is more responsive to an earlier vaccination campaign than to increased vaccination coverage, the result of shifting new vaccinations to time periods with high infection burden. In contrast, the winter wave benefits more from increased vaccination coverage than an earlier campaign. These results provide new insights into optimal vaccination strategies to tackle the strong biannual seasonality of COVID-19 burdens^15^ across different regions and should be considered for maximum impact of vaccination, particularly given variability of summer/winter burden across the US. It is also worth noting that while this work focuses on COVID-19 in the US, the underlying framework and insights are more broadly applicable as COVID-19 in other countries ^23^ as well as many other respiratory pathogens ^24–26^ exhibit spatiotemporal heterogeneity in transmission and burden, and would benefit from tailored vaccination strategies rather than a single uniform schedule.

Our work has several limitations. First, our scenario design doesn’t consider the effects of increasing vaccine uptake. Scenario E specifies the highest national coverage based on historical data, 42.8% in high-risk individuals and 24.6% overall (Table S2). While higher uptake would likely reduce acute disease the most, changing vaccine hesitancy is often quite difficult and therefore was not a focus. Second, our ensemble model has high uncertainty on the projected summer wave size. This is partially due to the lack of robust calibration data available for summer and fall 2024, during which NHSN hospitalization reporting was paused^27^, and variability in how individual models handled this issue. Despite these uncertainties, national projections accurately captured the seasonal distribution of reported hospitalizations in 2025–2026 (Figure S2), lending support to our conclusion regarding vaccination timing. Although we cannot directly evaluate the accuracy of these scenario projections due to reality not perfectly matching any single scenario, our projections appear to be somewhat inflated. This overestimation is possibly a result of changes not accounted for in models, including reductions in reporting and continued declines in susceptibility and severity, as COVID-19 transitions to endemicity. Fortunately, this overestimation should primarily affect estimates of absolute vaccination benefits, and relative effects (percent averted) should be less sensitive to these issues. Third, we remain in the early stages of understanding the mechanisms of COVID-19 biannual seasonality. There is substantial spatial heterogeneity in the summer wave across the US, including in timing and intensity.^22^ A more comprehensive characterization of this biannual seasonality is needed to design and implement more effective COVID-19 interventions, such as precise vaccination timing and coverage for different locations. Finally, our projections share the common limitation of long-term models that overlook continual viral evolution where emerging variants or subvariants with distinct epidemiological characteristics may exhibit changing dynamics.

Given rapid viral evolution, SARS-CoV-2 is likely to continue to cause substantial disease burden, including hospitalizations and deaths in the US and around the world. In this work we found that the 2025–26 season, and possibly future seasons, would likely continue to be characterized by two waves nationally, in summer and winter, though the exact drivers of these dynamics remain uncertain. With this biannual burden, COVID-19 presents an important question for policy and public health: should we shift away from the traditional fall vaccination campaign timing, and when would be the ideal timing to maximize the value of the COVID-19 vaccine? While the evidence from this work points to earlier vaccination campaigns in locations that experience greater relative burden of disease in the summer, further investigation is needed. This work highlights the importance of empirically-relevant modelling efforts that can provide essential insight to assess and guide policy, potentially helping to maximize the benefits of declining resources like vaccination.

## Methods

Eight teams contributed weekly projections of COVID-19 hospitalizations and deaths by state and nationally during a 52-week projection horizon from 27 April 2025 to 25 April 2026; projections and aggregated results were released in June 2025. Teams calibrated independent models using weekly COVID-19 hospitalizations and deaths from the National Healthcare Safety Network (NHSN)^27^ and National Centre for Health Statistics (NCHS),^28^ with calibration specifics (e.g., timeframe for calibration or inclusion of additional data sources) at the teams’ discretion ensuring that no data from the projection period was used, and submitted 150–300 trajectories (e.g., randomly sampling from a range of parameter values or stochastic iterations) per target, scenario, location, and age-group.

### Trajectory pairs:

To ensure comparability between scenarios, teams were required to ‘pair’ trajectories across scenarios, targets, horizons and age groups. Pairing dictates that all model parameters outside of the scenario assumptions remain the same, such that an individual trajectory in a scenario is comparable to its paired trajectory in the other scenarios. The pairing requirement plus a counterfactual Scenario A (no vaccination recommendation) enables estimation of the number and percent of averted hospitalizations and deaths.

### Ensemble methodology:

Individual team’s model projections were combined to produce ensemble projections using a trimmed linear opinion pool method,^29^ and estimates of burden averted under different mitigating scenarios were obtained using meta-analysis approaches.^6^ For both hospitalizations and deaths, eight models contributed to the ensemble.

### Scenario design:

Five scenarios (Table 1) were considered across two axes: vaccination group recommendations and timing of the vaccination campaign. For vaccination groups, three levels were considered: i) no recommendations; ii) recommendations for high-risk group only, including individuals over 65 years and those under 65 years with pre-existing medical conditions, such as chronic obstructive pulmonary disease (COPD), diabetes, chronic kidney diseases;^30^ and iii) universal recommendation for all eligible individuals across age groups. Vaccination uptake was set to 2024–25 coverage levels for vaccination groups in each scenario (Figure S1). For vaccination timing, we considered two start times for all locations: i) classic timing, with the vaccine roll-out aligned with the 2024–25 campaign (i.e., 15 August 2025), and ii) an earlier timing, where the vaccination campaign would start 1.5 months earlier (i.e., 29 June 2025). In all scenarios, updated vaccines were expected to match the predominant SARS-CoV-2 variants circulating on 30 June 2025. The vaccine effectiveness (VE) against COVID-19 hospitalization was assumed to be 45% at the start of the vaccination campaign, consistent with a recent analysis of US COVID-19 hospitalizations.^31^ VE against infection and death and the pace of immune escape were left to the scientific discretion of the teams.^32^ We did not consider the impact of a new variant with rapid, significant antigenic change, nor substantial increase in transmissibility (akin to Delta or Omicron variants). Severity of future circulating variants in naive populations was assumed to remain similar to the Omicron lineages.

### Trajectory timing analysis:

To understand the relationship between epidemic and vaccination timings, and how it affects COVID-19 burden–for example, understanding whether the combination of an earlier epidemic and vaccination campaign leads to lower burden–we characterized timings of individual model projections at the level of the trajectories. Trajectory timings were quantified in two ways: 1) continuous - median hospitalization week (MHW, defined as the first epidemic week reaching ≥50% cumulative hospitalizations) and 2) categorical - using the percentage of cumulative hospitalizations occurring by epidemic week (EW) 43, as on average, it is the midpoint between summer and winter waves. Models (model number: 4, 5–8) that provided fewer than 300 trajectories were upsampled for a consistent comparison across all models (Supplementary Methods 1C). Trajectories were defined as “summer-dominant” if more than 55% of cumulative hospitalizations occurred by EW 43, “winter-dominant” if fewer than 45%, or “similar” if between 45–55% accrued. Frequency of trajectory timing classifications at both national and state levels were analyzed. Then, using Scenario A as the reference group, we systematically quantified the percent hospitalization averted in Scenarios B through E. We also computed the percent of averted hospitalizations in earlier vs. regular vaccine timing in scenarios with the same vaccine eligibility criteria (i.e., Scenario C vs. B, and D vs. E). We focus on hospitalizations for this analysis as deaths are highly correlated with it.

### Analysis of vaccination scenario effects on summer and winter waves:

We also investigated the effect of the vaccination scenarios on the summer and winter wave sizes. A mixed-effects model was fitted to the cumulative ensemble estimates of the total, summer and winter wave sizes, with scenarios as a fixed effect and locations (i.e., states) and teams as a random effect. The coefficients of the fixed effects (i.e., the vaccination scenarios) correspond to the number of hospitalizations averted per 100,000 population relative to the reference Scenario A. Supplementary analyses were conducted at the level of individual models to control for model-specific effects, state specific effects related to historically higher summer or winter outbreaks, and variation in vaccination coverage (Supplementary Methods 1E,F).

All analyses were performed in *Python* and *R* and the analysis code is freely available at: https://github.com/midas-network/covid19-scenario-hub_r19_manuscript. Authors used ChatGPT and Claude to assist with writing some of the analysis code. After using these tools, the authors reviewed and edited the content as needed and take full accountability for the final content.

## Supplementary Material

Supplement 1

## Figures and Tables

**Table 1.** COVID-19 projection scenario definitions for Round 19 of the US Scenario Modeling Hub, 27 April 2025–25 April 2026. Scenario specifications focus on the impact of different target group recommendations for updated COVID-19 booster vaccinations (rows) combined with different timing of booster vaccination (columns) for the 2025–2026 season. Modelling teams were instructed to provide projections for hospitalizations and deaths in 50 states, D.C., and at the national level under these five scenarios.

	Vaccine recommended, same timing of immunization as last year campaign	Vaccine recommended, early timing of immunization campaign
**No vaccine recommendation**	**Scenario A** No new vaccinations during 2025–26 season	
**Annual vaccination recommended for high-risk groups (65+ and those with underlying risk factors)**	**Scenario B** • Vaccination starts 15 August 2025 • Vaccine has 45% VE against hospitalization at time of initial roll-out • Uptake in high risk groups (individuals 65+ or with underlying risk factors) is the same as seen for 2024–25 • Uptake in all other groups is negligible	**Scenario C** • Vaccination starts 29 June 2025 (1.5 month earlier than in 2024–25) • Vaccine has 45% VE against hospitalization at time of initial roll-out • Uptake in high risk groups (individuals 65+ or with underlying risk factors) is the same as seen for 2024–25 • Uptake in all other groups is negligible
**Annual vaccination recommended for all currently eligible groups**	**Scenario D** • Vaccination starts 15 August 2025 • Vaccine has 45% VE against hospitalization at time of initial roll-out • Coverage saturates at levels of the 2024–25 booster for all population subgroups (approximately 22% of adults nationally, 44% of 65+, 26% of high risk individuals 18–64 yrs)	**Scenario E** • Vaccination starts 29 June 2025 (1.5 months earlier than in 2024–25) • Vaccine has 45% VE against hospitalization at time of initial roll-out • Coverage saturates at levels of the 2024–25 booster for all population subgroups (approximately 22% of adults nationally, 44% of 65+, 26% of high risk individuals 18–64 yrs)
