# Supplementary material for: Assessing the Impact of Timing and Coverage of United States COVID-19 Vaccination Campaigns: A Multi-Model Approach
Source: medRxiv. 2026 Apr 8:2026.04.07.26349269. Preprint. [Version 2] doi: 10.64898/2026.04.07.26349269 (PMC13086075; doi:10.64898/2026.04.07.26349269)
Supplement: Supplementary file 1 [file NIHPP2026.04.07.26349269v2-supplement-1.pdf]

medRxiv preprint doi: https://doi.org/10.64898/2026.04.07.26349269; this version posted April 8, 2026. The copyright holder for this preprint (which was not certified by peer review) is the author/funder, who has granted medRxiv a license to display the preprint in perpetuity.
It is made available under a CC-BY 4.0 International license .

Supplementary Materials

Table of Contents

1. Supplementary Methods
    a. Modeling team descriptions
    b. Quantifying national vaccination coverages
    c. Upsampling model trajectories for timing analysis
    d. Association between the sizes of summer and winter waves
    e. Mixed effects analysis per model for summer and winter leaning locations
    f. Division of states into low, medium, and high coverage
2. Supplementary Results
    a. Characterization of trajectory timing by classification
    b. Categorical epidemic timing analysis
    c. Association between summer and winter wave sizes
    d. Scenario effects on hospitalizations per team
3. Supplementary Figures
4. Supplementary Tables
5. Code availability
6. Supplementary references

---

1. Supplementary Methods

1A. Modeling Team Descriptions

A total of eight models submitted to this scenario modeling challenge (Table S1). The types of models ranged from mechanistic compartmental approaches to hybrid Fourier transformation and compartmental approaches. While the scenario design required the same assumptions about vaccine timing, coverage, and efficacy against hospitalization, modeling teams had different assumptions around population structure, natural history, immunity against infection, seasonality, and calibration process. Full details on model descriptions and assumptions can be found here: https://docs.google.com/spreadsheets/d/12VFgTtOoyTN806E_I6q1ufUX-vXv07MvdVcmXFtAjBY/edit?gid=0#gid=0

Model Label	Model Name	Institution	Model Type	Reference
Model 1	CFA-Scenarios	CDC CFA	Meta-population, compartmental model	
Model 2	JHU_UNC-flepiMoP	Johns Hopkins University and University of North Carolina at Chapel Hill	Mechanistic age-specific compartmental model	1
Model 3	MOBS_NEU-GLEAM_COVID	Northeastern University	Meta-population, age-specific, stochastic compartmental model	2
Model 4	PSI-M_CoV_2025	Predictive Sciences	Mechanistic age-specific compartmental model	

medRxiv preprint doi: https://doi.org/10.64898/2026.04.07.26349269; this version posted April 8, 2026. The copyright holder for this preprint (which was not certified by peer review) is the author/funder, who has granted medRxiv a license to display the preprint in perpetuity.
It is made available under a CC-BY 4.0 International license .

Model 5	UIUC-IMMCYC	University of Illinois - Urbana-Champaign	Mechanistic age-stratified compartmental model	3
Model 6	UNCC-Hierbin	University of North Carolina - Charlotte	Fourier transformation and compartmental hybrid model	4
Model 7	UT-ImmunoSEIRS	University of Texas at Austin	Mechanistic age-stratified compartmental model	5
Model 8	UVA-adaptive	University of Virginia	Meta-population, compartmental model	6

Table S1: Table mapping the description of the models to the labels used in the analysis in the main text.

1B. Quantifying national vaccination coverages

In this study, we consider scenarios with three different levels of vaccination: no vaccination (counterfactual - scenario A), high-risk (scenarios B and C), and all-eligible (scenarios D and E). In addition to scenario and location differences, vaccine uptake time-series are stratified by age group and high/low risk populations (see Figure S1). Table S2 summarizes these differences by aggregating total doses (millions) across locations and age groups. Percent population coverage appears in parenthesis. The counterfactual scenario A is excluded from the table because it specifies no doses administered.

Scenario	Low Risk	High Risk	Totals
B - high risk, classic timing	0 (0.0%)	38.5 (39.8%)	38.5 (11.8%)
C - high risk, early timing	0 (0.0%)	41.4 (42.8%)	41.4 (12.7%)
D - all eligible, classic timing	35.4 (15.4%)	38.5 (39.8%)	73.9 (22.7%)
E - all eligible, early timing	38.8 (16.9%)	41.4 (42.8%)	80.3 (24.6%)

Table S2: National vaccine doses (millions) by scenario and risk population. For each scenario specification, millions of doses are listed for each risk group. Additionally, the risk-population percentage uptake appears in parentheses.

1C. Upsampling model trajectories for timing analysis

The scenario specification stated that teams must submit 150 to 300 trajectories for each location and scenario. As a result, some teams submitted 150 trajectories, and some submitted 300. For the analysis of trajectory timing by model, we upsampled such that each team had equal representation in the distribution of trajectory timings. Because we only had two trajectory counts (150 and 300), upsampling was implemented as a simple duplication of trajectories from models with a 150 count.

1D. Association between the sizes of the summer and winter waves

To investigate the association between winter and summer wave sizes, we split each trajectory at EW 43 for each model, location and scenario. Summer wave size was calculated as the cumulative number of hospitalizations that occurred until the end of EW 42. Similarly, winter wave size was the cumulative number of hospitalizations from EW 43 through the end of the simulation horizon. As an exploratory analysis of the biannual seasonality of COVID-19 hospitalizations, associations between the summer and winter wave sizes for each team, location, and scenario were determined by the Pearson's correlation coefficient.

medRxiv preprint doi: https://doi.org/10.64898/2026.04.07.26349269; this version posted April 8, 2026. The copyright holder for this preprint (which was not certified by peer review) is the author/funder, who has granted medRxiv a license to display the preprint in perpetuity.
It is made available under a CC-BY 4.0 International license .

1E. Mixed effects analysis per model for summer and winter leaning locations

We investigated how sizes of summer and winter waves differed across scenarios. A location was tagged as summer-leaning or winter-leaning based on which wave had more than 50% of cumulative hospitalizations on average across all the Scenario A trajectories. A mixed-effects model was fitted separately to summer and winter leaning locations, with scenarios as a fixed effect and locations (i.e., states) as a random effect. The 'effect' was the difference in number of hospitalizations per 100,000 population relative to the reference Scenario A. We report the number of hospitalizations averted per 100,000 relative to Scenario A.

1F. Division of states into low, medium and high coverage

In our scenario analysis, the baseline vaccination coverage level for each state was assumed to equal that of the 2024-2025 vaccination campaign. Given that there was significant variation in the baseline coverage levels ranging from less than 10% of a state receiving the booster vaccine to greater that 35% (Figure S24), for each team, we repeated the mixed-effects analysis separately for states grouped into low, medium, and high vaccination coverage. This grouping was based on the historical coverage levels and states were divided into tertiles (so that each group contained one-third of the locations). Note, as in the main text analysis, states were also further divided into the summer and winter dominant categories resulting in six separate mixed-effects models that were fit for each team for each variable of interest (for e.g., the total epidemic size) using Scenario A as baseline, the other scenarios as fixed effects, and the particular subset of states as a random effect (Figures S25-S26).

2. Supplementary Results

2A. Characterization of trajectory timing

To characterize the timings of model trajectories we used median hospitalization week (MHW) and a seasonality classification where trajectories are classified as having a stronger summer wave, stronger winter wave, or similarly sized waves. Applying the same methods to national observed hospitalization data for the previous four seasons yields the results in Table S3. Reporting was voluntary—and significantly lower—during a long period of 2024, so the true timing of the 2024-25 season is likely earlier than the observed timings. However—ignoring the 2024-25 season—we see MHWs varying from epidemic week 40 to 48, and one season each classified as Summer, Similar, and Winter.

Season	MHW	Seasonality
2021-22	46	Similar
2022-23	40	Summer
2023-24	48	Winter
2024-25*	51	Winter

Table S3: National NHSN observed MHW for confirmed COVID-19 hospital admissions. **NHSN reporting was not mandatory from May 1, 2024 (EW 18) until October 30, 2024 (EW 48). The metrics presented here for 2024-25 are useful for comparison to the observed data, but likely not a good characterization of the actual season timing.*

In the main text we generally avoided drawing timing-related conclusions from the ensemble, but it is interesting to note how scenarios affected trajectory timing. Table S4 contains the median MHW across all models for national projections, by scenario. Scenarios with early vaccine timing (C and E) reduced the summer wave, resulting in a slightly later MHW. The scenario with no vaccination (A) results in larger winter waves, and thus later MHW.

Scenario	Median MHW

medRxiv preprint doi: https://doi.org/10.64898/2026.04.07.26349269; this version posted April 8, 2026. The copyright holder for this preprint (which was not certified by peer review) is the author/funder, who has granted medRxiv a license to display the preprint in perpetuity.
It is made available under a CC-BY 4.0 International license .

A	47
B	45
C	46
D	44
E	47

Table S4: National ensemble median trajectory MHW for each scenario.

When classifying national trajectory timings by summer, similar, and winter, all scenarios produced more winter- than summer-dominant ensemble trajectories (Figure S9). However, the proportion of winter-dominant trajectories was substantially lower in the classic (B: 42% and D: 40%) vs early (C: 50% and E: 50%) vaccination scenarios. Winter-dominant trajectories were also more prevalent than summer-dominant at the subnational level (Figure S10). Looking specifically at the counterfactual (Scenario A), 44 of 51 locations exhibited a higher proportion of winter-dominant trajectories.

2B. Categorical epidemic timing analysis

This section contains extended results using categorization of epidemic timings. At the national level, Figure S19 illustrates percent hospitalization averted for each model and scenario, grouped by epidemic timings: summer-dominant, winter-dominant, and similar. Looking more directly at the effect of vaccine timing, Figure S20 depicts the difference between percent hospitalizations averted for early and classic vaccination timing with one panel each for high-risk (scenario C minus scenario B) and all-eligible (scenario E minus scenario D) vaccine strategies. For each panel, 14 of 16 model-timing combinations show an increase in percent averted in earlier vaccination timing scenarios. Four out of five models with coverage across more than one timing group show a positive median averted-difference for earlier trajectory timings.

Extending the analysis to state-level, Figure S21 compares the impact of vaccine timing vs categorized epidemic timing for the full ensemble of models. The difference in percent hospitalizations averted (early minus classic vaccine timing) is shown for each subnational location. The top panel takes the difference of the two high-risk vaccine scenarios (C-B), and the bottom panel compares the all-eligible scenarios (E-D). In almost every location-timing combination the median outcome results in more hospitalizations averted under the early vaccine scenario. For high-risk vaccination (top panel), median outcomes are positive for 49 (50, 51) of 51 locations for summer (similar, winter) epidemic timings. For all-eligible vaccination (bottom panel), median outcomes are positive for 49 (49, 51) of 51 locations for summer (similar, winter) epidemic timings.

2C. Association between summer and winter wave sizes

The association between summer and winter wave sizes was assessed using Pearson's correlation coefficient r for each model at national and state levels for each scenario (Figure S22). Here, summer wave size corresponds to the number of hospitalizations up to (including) EW 42 for each trajectory and winter wave size is the number of hospitalizations from EW 43 till the end of the projection period. Three out of eight models (2, 3, and 6) reported a predominantly positive association between the two waves, i.e., a large summer wave implies a large winter wave, although variability exists at the state level. Sizes of the two waves were predominantly negatively associated for the other five models. These patterns are consistent across all five scenarios.

We speculate this variability in the sizes of the summer and winter wave results from a complicated interplay between the force of infection (primarily driven by intrinsic viral transmissibility and seasonality) and immune dynamics, for example, waning of prior infection- or vaccine-induced immunity. While the exact contribution of each mechanism is difficult to disentangle, as models implement them differently, these results suggest immune

medRxiv preprint doi: https://doi.org/10.64898/2026.04.07.26349269; this version posted April 8, 2026. The copyright holder for this preprint (which was not certified by peer review) is the author/funder, who has granted medRxiv a license to display the preprint in perpetuity.
It is made available under a CC-BY 4.0 International license .

dynamics likely drive the winter wave for those models with a predominantly negative association. This could be the result of a large summer wave depleting the susceptible population, leading to a smaller successive winter wave. Wave sizes can be explained by the force of infection for models with a positive association between the two waves, with both waves either high or low depending upon intrinsic viral transmissibility and/or assumptions about immunity waning in the model.

2D. Vaccination scenario effects on hospitalizations per team

For each team's projections, a mixed effects model (with the scenarios as a fixed effect and the state as a random effect) was deployed to investigate vaccination scenario effects on total, summer and winter wave sizes, with Scenario A (no vaccination) as the reference (Figure S23). These effects were calculated separately for states with historically larger summer or winter waves (see Suppl. Methods 1E). Vaccination effect on total epidemic size was larger for winter leaning states, driven by larger reductions in winter wave size across scenarios and models, except Model 4. There were 13.78 (average range across scenarios: 11.91-15.78) more hospitalizations per 100,000 during the winter waves in winter leaning states than summer leaning ones. Scenario effects on summer wave size were similar for summer and winter leaning states, with an average 2.34 (range: 1.35-3.72) more averted hospitalizations per 100k in the winter leaning states. A similar analysis was also done where states were instead divided by historical vaccine coverage levels (see Figure S24 and Suppl. Methods 1F) and the qualitative trends were unaffected (Figures S25-26).

medRxiv preprint doi: https://doi.org/10.64898/2026.04.07.26349269; this version posted April 8, 2026. The copyright holder for this preprint (which was not certified by peer review) is the author/funder, who has granted medRxiv a license to display the preprint in perpetuity.
It is made available under a CC-BY 4.0 International license .

3. Supplementary Figures

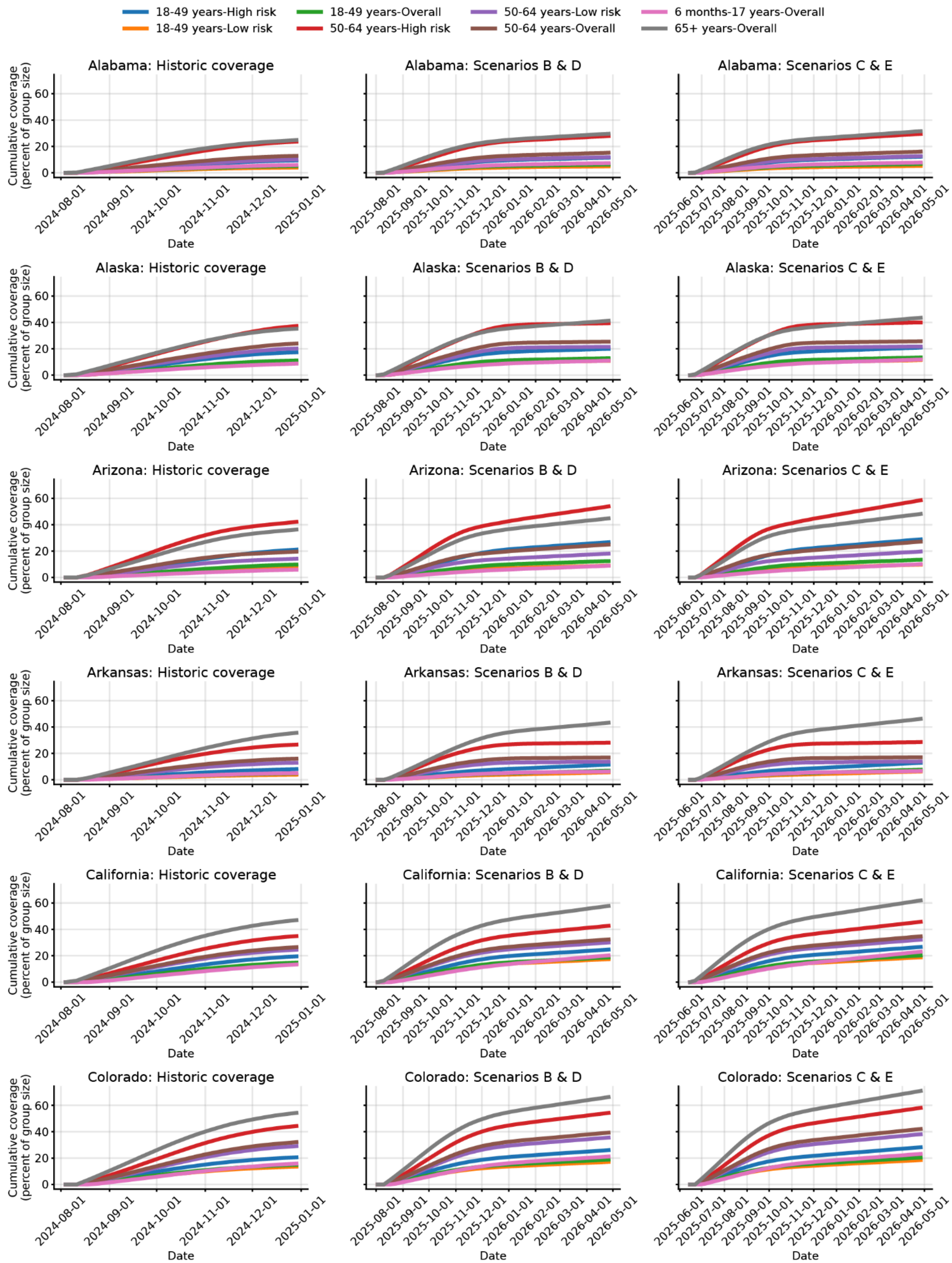

medRxiv preprint doi: https://doi.org/10.64898/2026.04.07.26349269; this version posted April 8, 2026. The copyright holder for this preprint (which was not certified by peer review) is the author/funder, who has granted medRxiv a license to display the preprint in perpetuity.
It is made available under a CC-BY 4.0 International license .

Figure S1. State and age specific vaccine coverage used for different vaccination scenarios from 1 Aug 2024 to 1 May 2026.

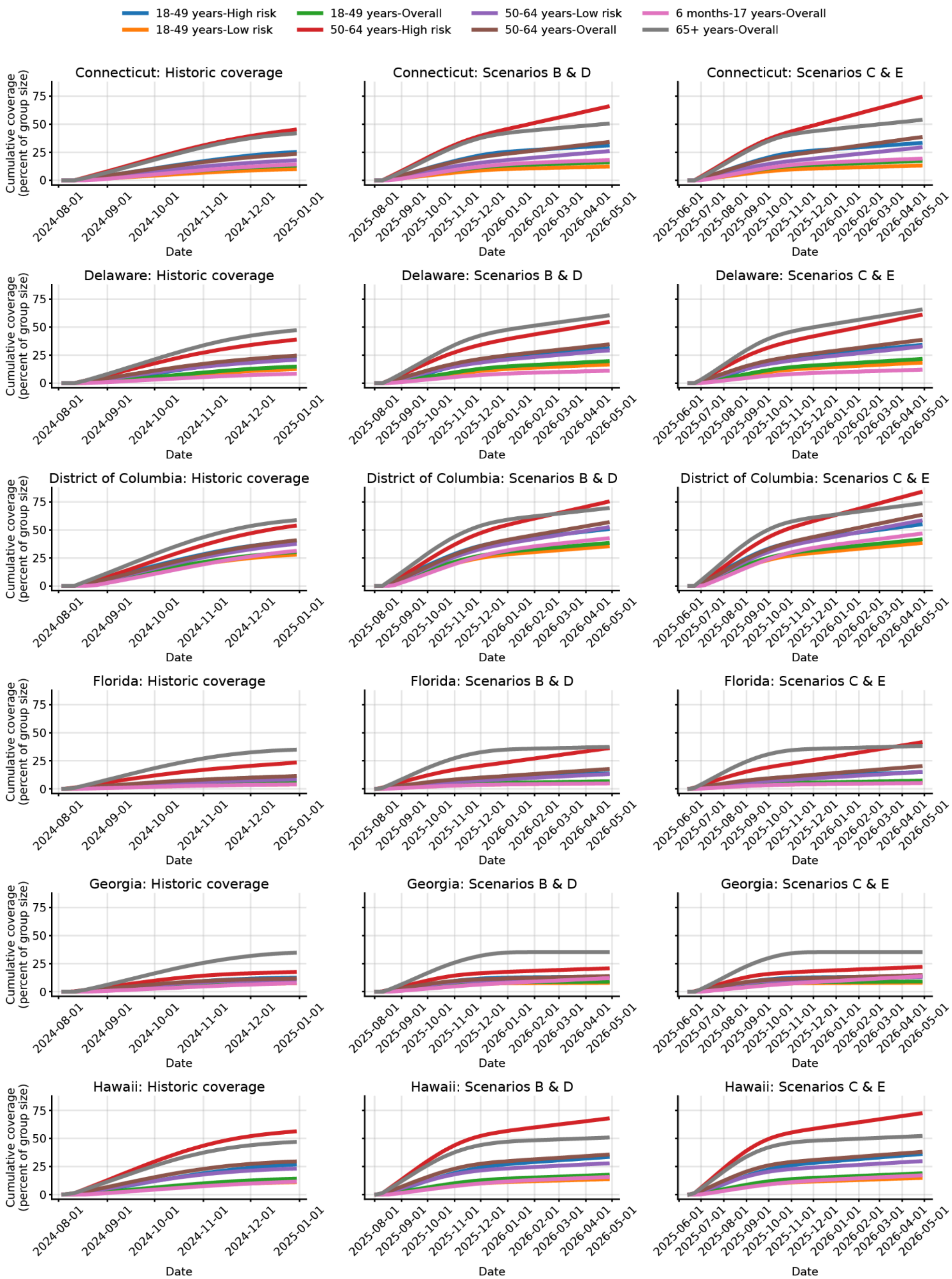

medRxiv preprint doi: https://doi.org/10.64898/2026.04.07.26349269; this version posted April 8, 2026. The copyright holder for this preprint (which was not certified by peer review) is the author/funder, who has granted medRxiv a license to display the preprint in perpetuity.
It is made available under a CC-BY 4.0 International license .

Figure S1 (contd)

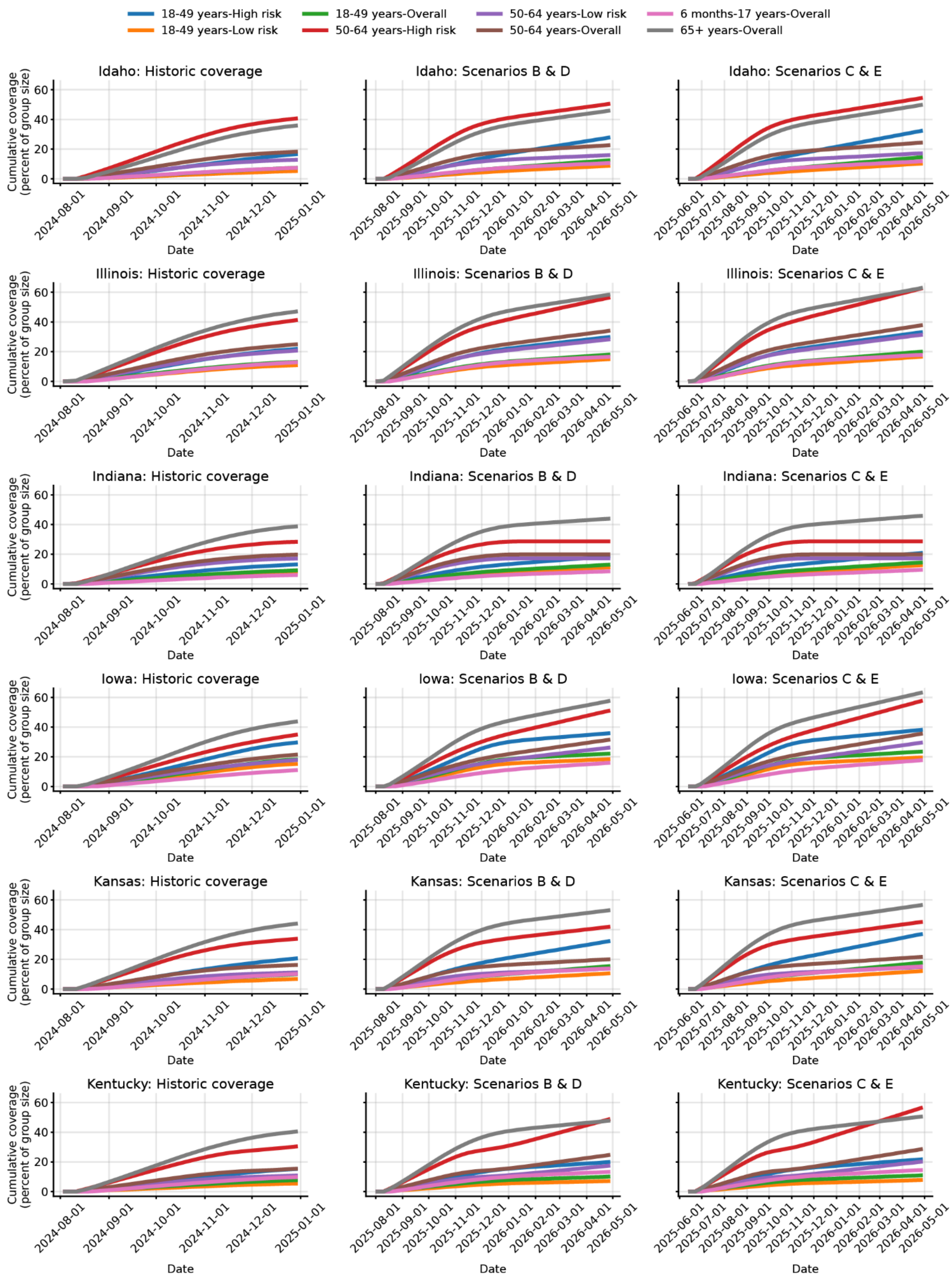

Figure S1 (contd)

medRxiv preprint doi: https://doi.org/10.64898/2026.04.07.26349269; this version posted April 8, 2026. The copyright holder for this preprint (which was not certified by peer review) is the author/funder, who has granted medRxiv a license to display the preprint in perpetuity.
It is made available under a CC-BY 4.0 International license .

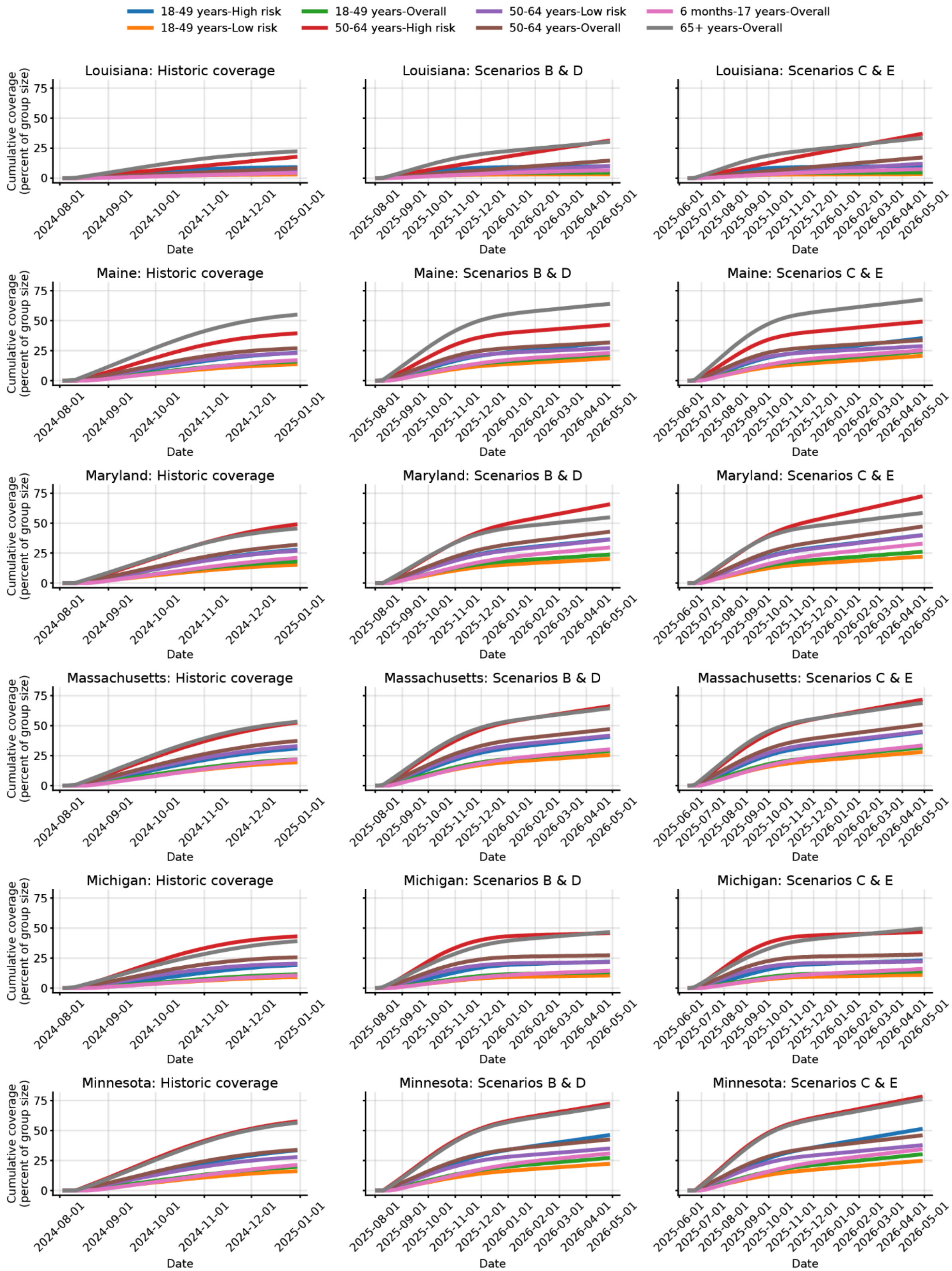

Figure S1 (contd)

medRxiv preprint doi: https://doi.org/10.64898/2026.04.07.26349269; this version posted April 8, 2026. The copyright holder for this preprint (which was not certified by peer review) is the author/funder, who has granted medRxiv a license to display the preprint in perpetuity.
It is made available under a CC-BY 4.0 International license .

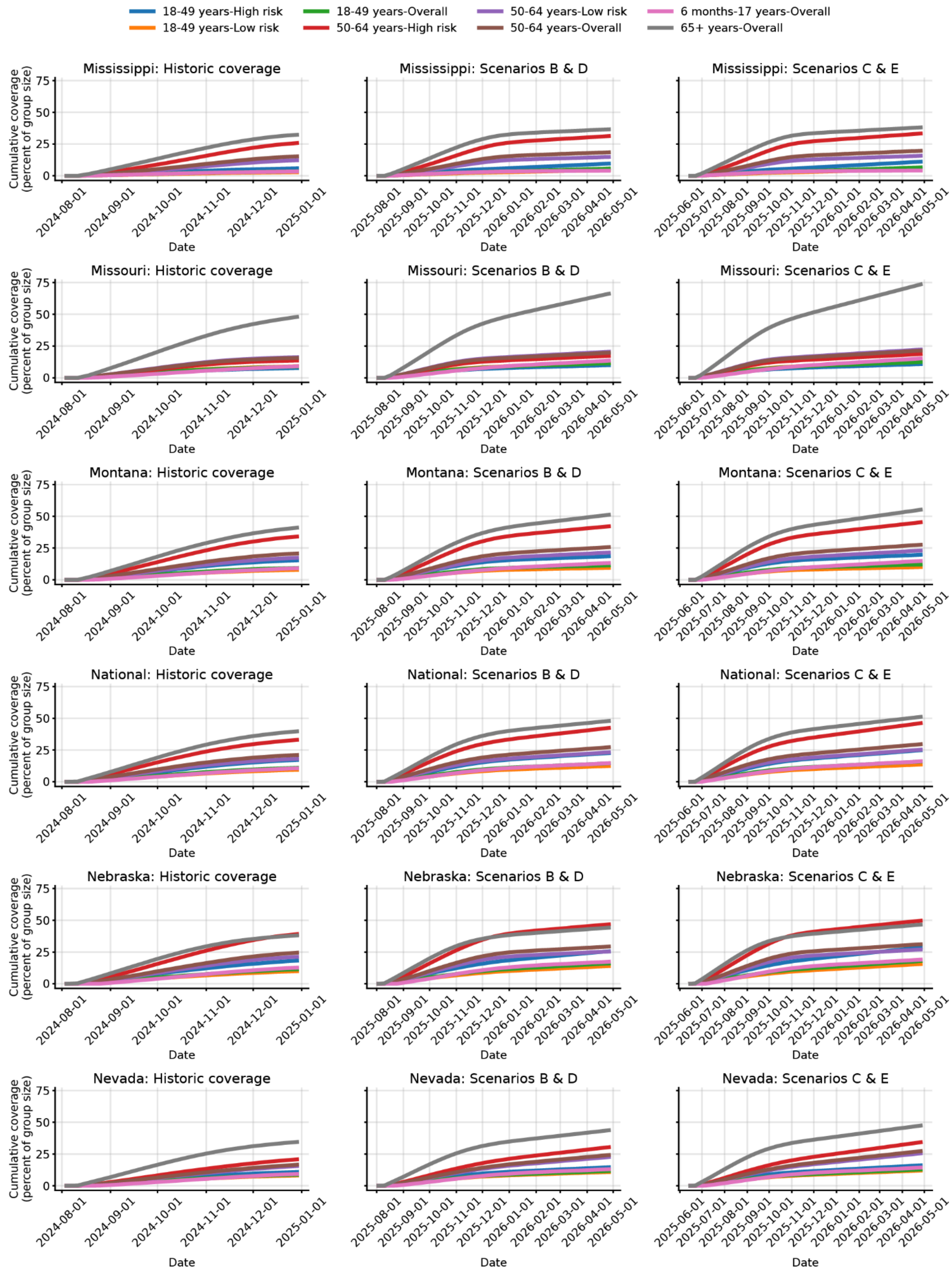

Figure S1 (contd)

medRxiv preprint doi: https://doi.org/10.64898/2026.04.07.26349269; this version posted April 8, 2026. The copyright holder for this preprint (which was not certified by peer review) is the author/funder, who has granted medRxiv a license to display the preprint in perpetuity.
It is made available under a CC-BY 4.0 International license .

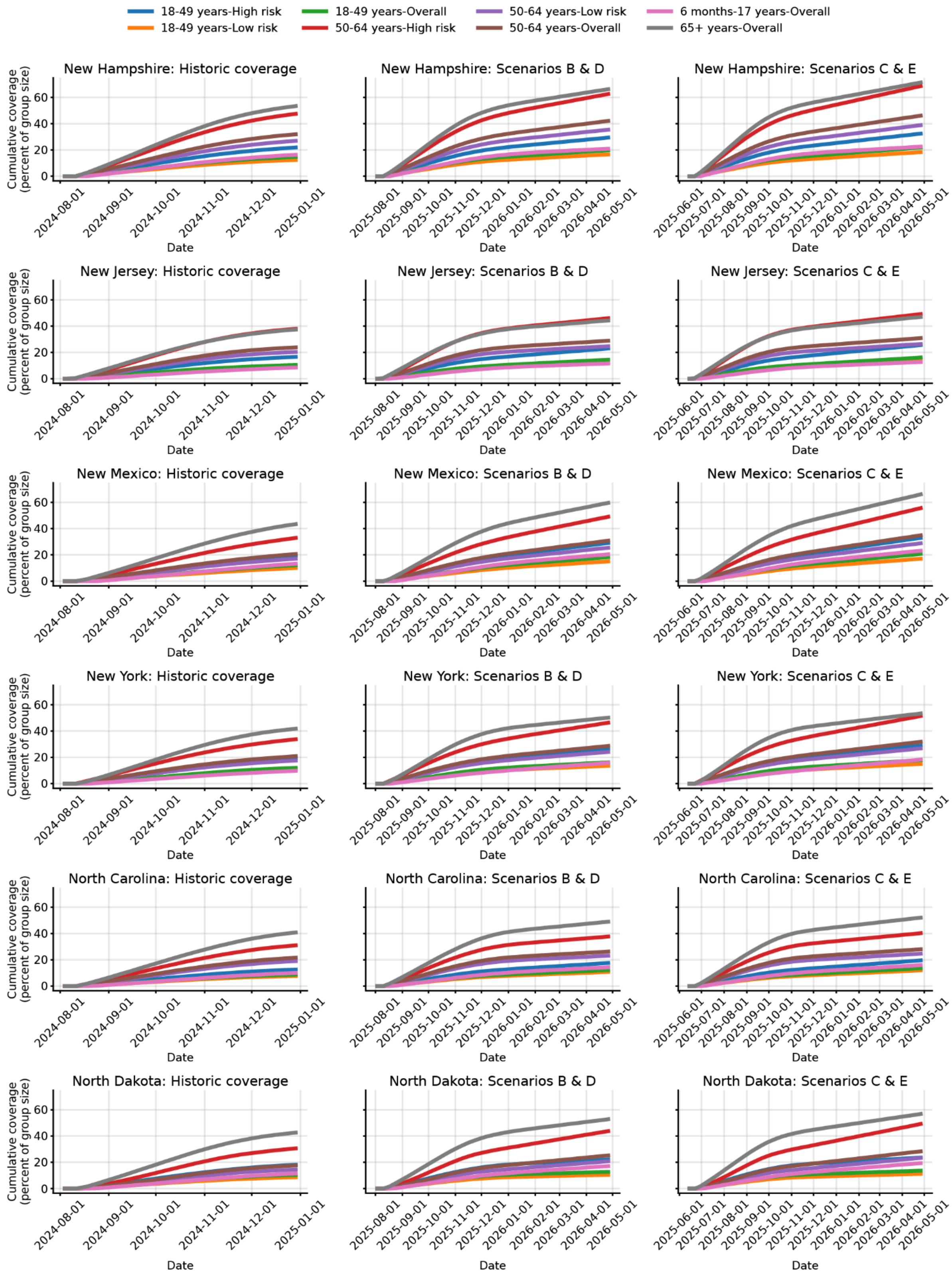

Figure S1 (contd)

medRxiv preprint doi: https://doi.org/10.64898/2026.04.07.26349269; this version posted April 8, 2026. The copyright holder for this preprint (which was not certified by peer review) is the author/funder, who has granted medRxiv a license to display the preprint in perpetuity.
It is made available under a CC-BY 4.0 International license .

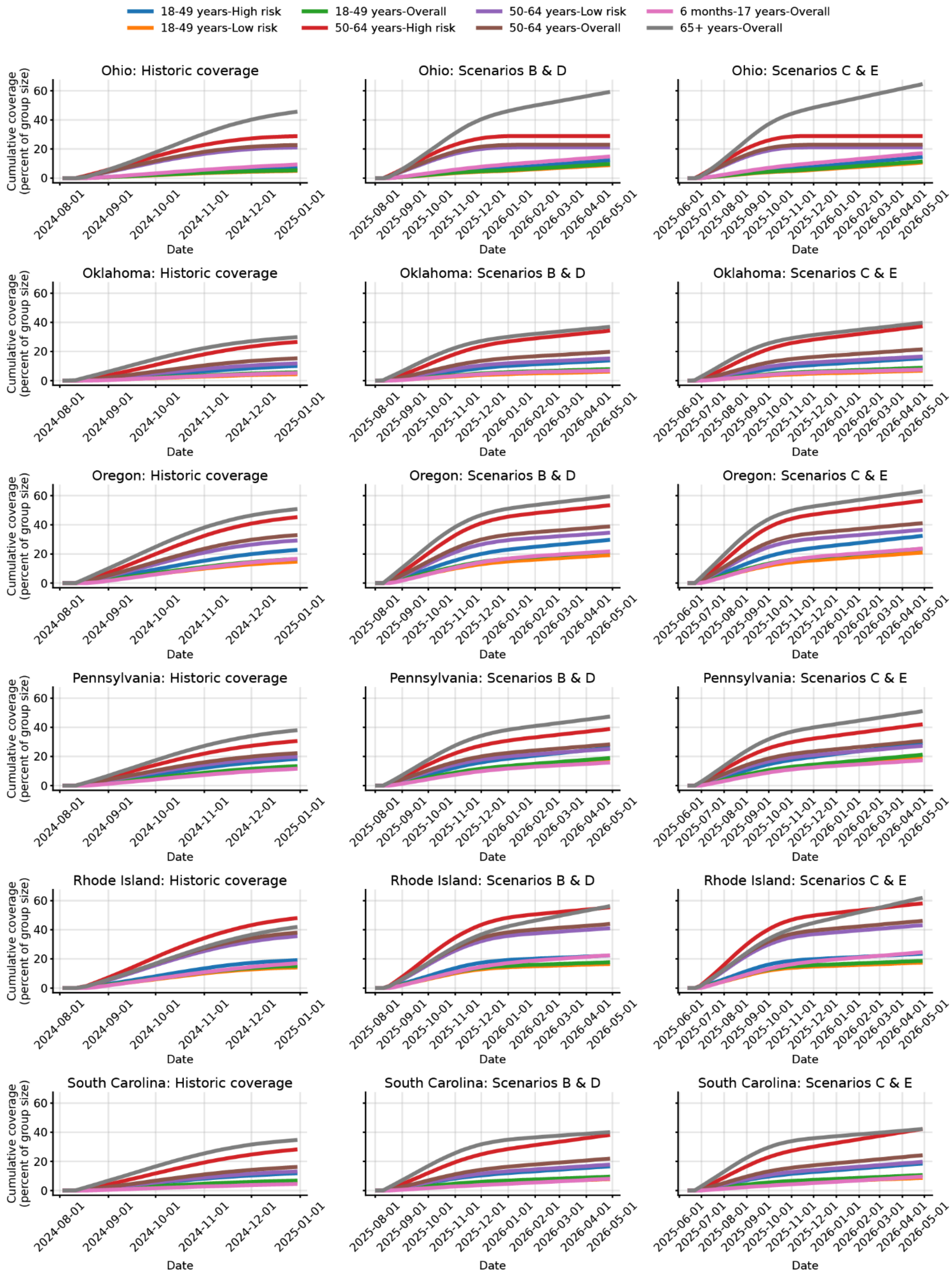

Figure S1 (contd)

medRxiv preprint doi: https://doi.org/10.64898/2026.04.07.26349269; this version posted April 8, 2026. The copyright holder for this preprint (which was not certified by peer review) is the author/funder, who has granted medRxiv a license to display the preprint in perpetuity.
It is made available under a CC-BY 4.0 International license .

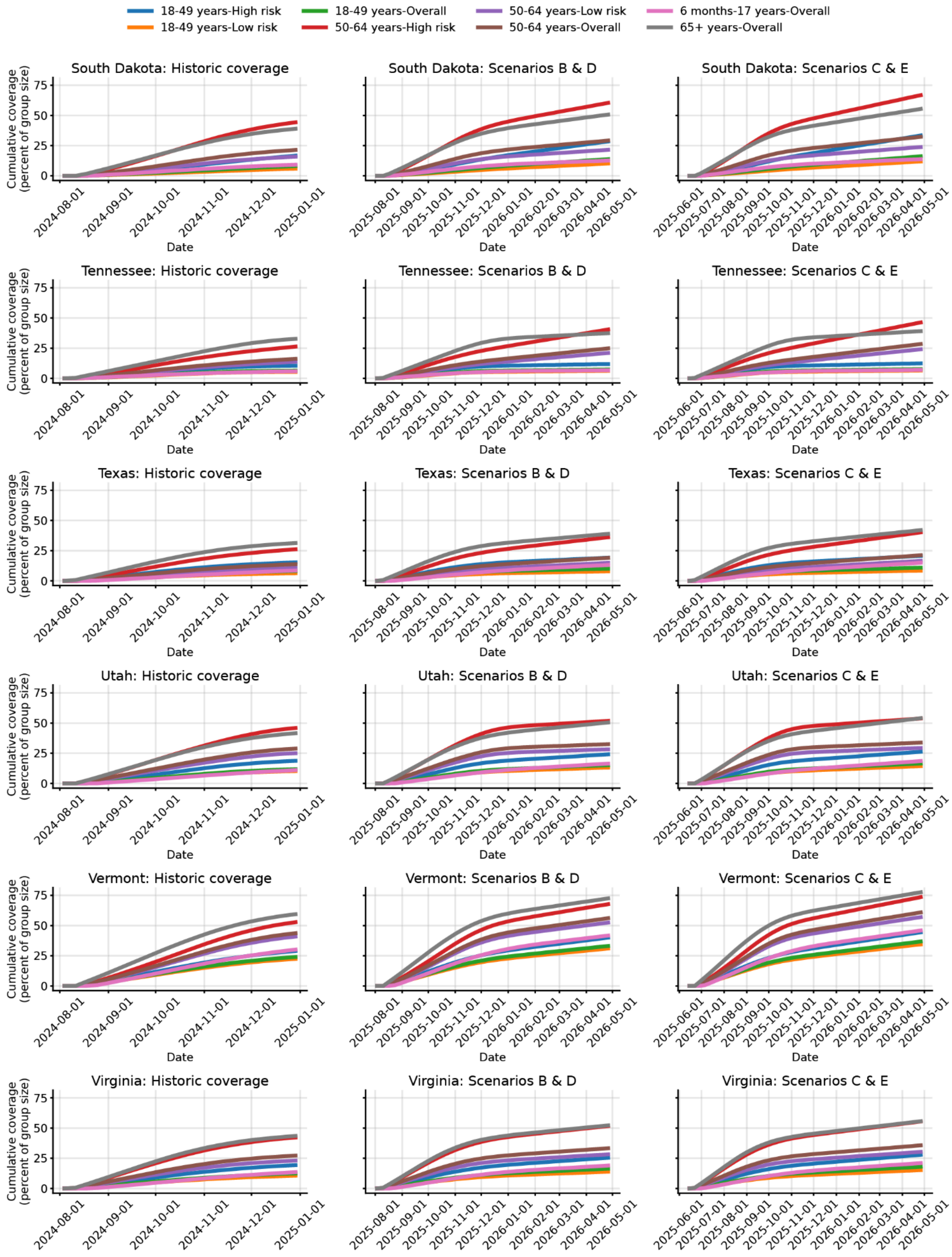

Figure S1 (contd)

medRxiv preprint doi: https://doi.org/10.64898/2026.04.07.26349269; this version posted April 8, 2026. The copyright holder for this preprint (which was not certified by peer review) is the author/funder, who has granted medRxiv a license to display the preprint in perpetuity.
It is made available under a CC-BY 4.0 International license .

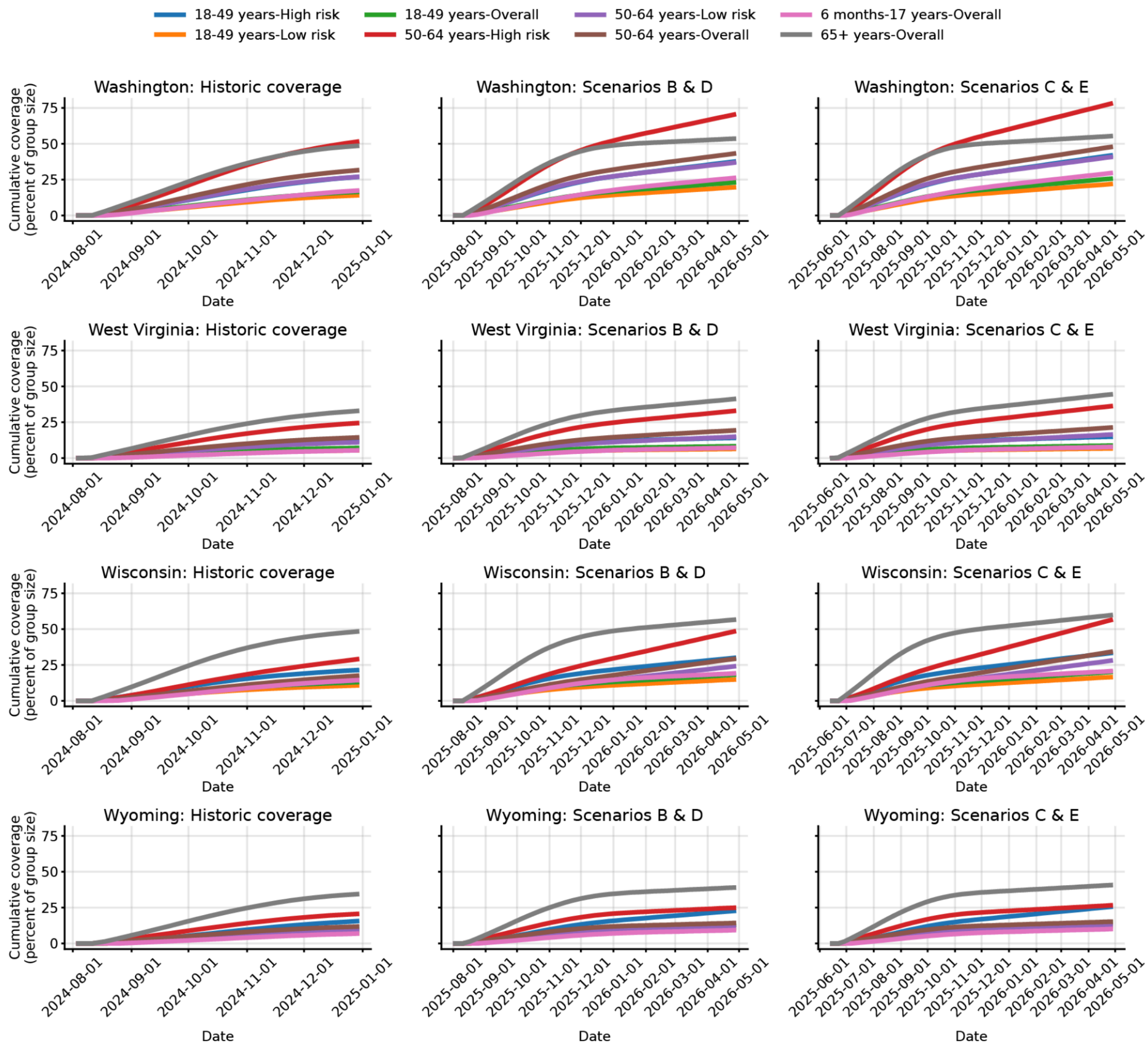

Figure S1 (contd)

medRxiv preprint doi: https://doi.org/10.64898/2026.04.07.26349269; this version posted April 8, 2026. The copyright holder for this preprint (which was not certified by peer review) is the author/funder, who has granted medRxiv a license to display the preprint in perpetuity.
It is made available under a CC-BY 4.0 International license .

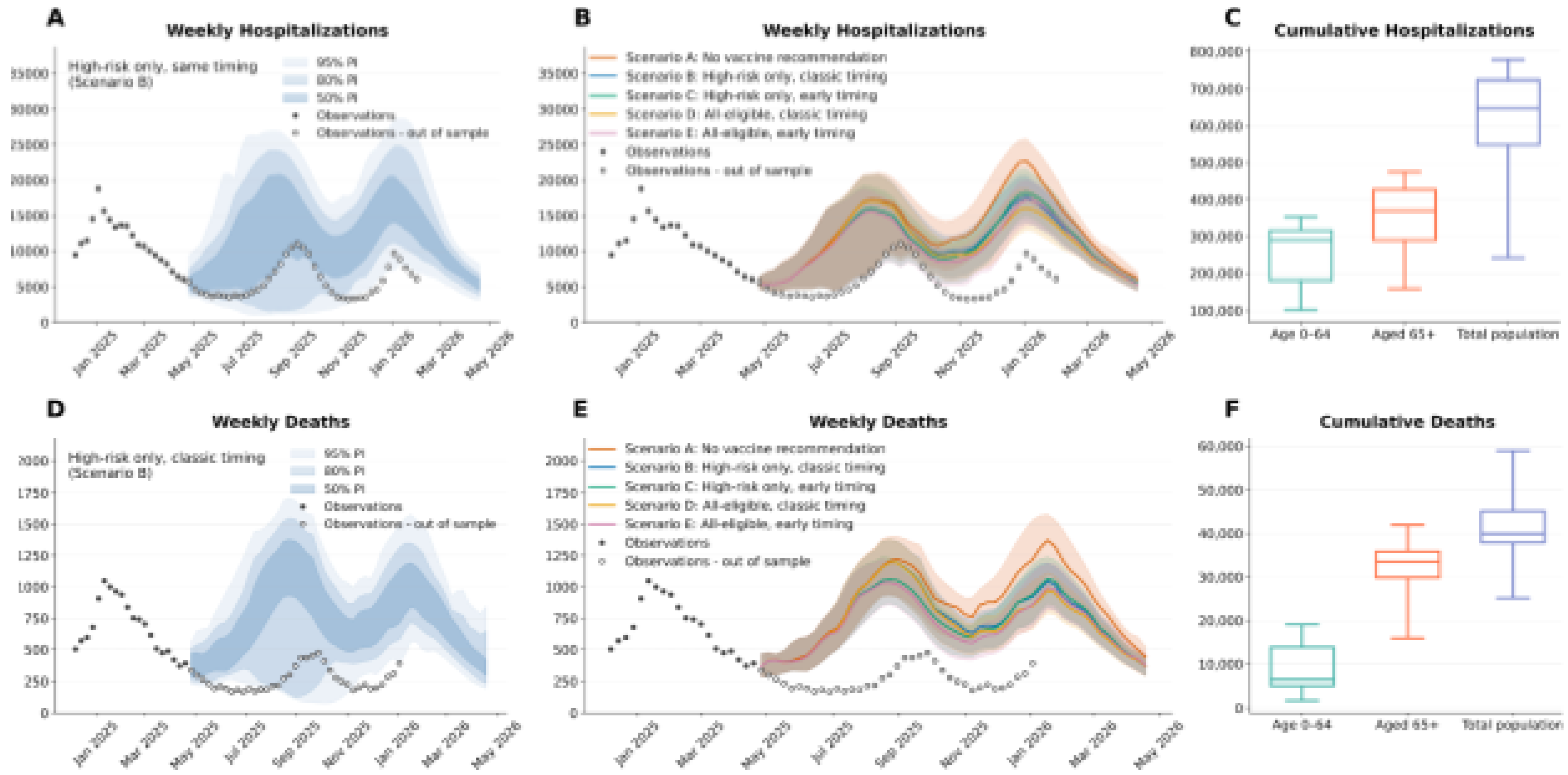

Figure S2. National-level ensemble predictions. A) Ensemble projections of weekly hospital admissions of Scenario B (High risk and classic timing). B) Combined hospital incidence projections across all scenarios. Shaded regions are the 50% projection interval and lines are median values. C) Cumulative hospitalizations across age groups, outer boxes represent the 50% projection interval and the whiskers represent the 95% PI. D) Ensemble projections of weekly deaths of Scenario B (High risk and classic timing). B) Combined weekly death projections across all scenarios. Shaded regions are the 50% projection interval and lines are median values. C) Cumulative deaths across age groups, outer boxes represent the 50% projection interval and the whiskers represent the 95% PI.

medRxiv preprint doi: https://doi.org/10.64898/2026.04.07.26349269; this version posted April 8, 2026. The copyright holder for this preprint (which was not certified by peer review) is the author/funder, who has granted medRxiv a license to display the preprint in perpetuity.
It is made available under a CC-BY 4.0 International license .

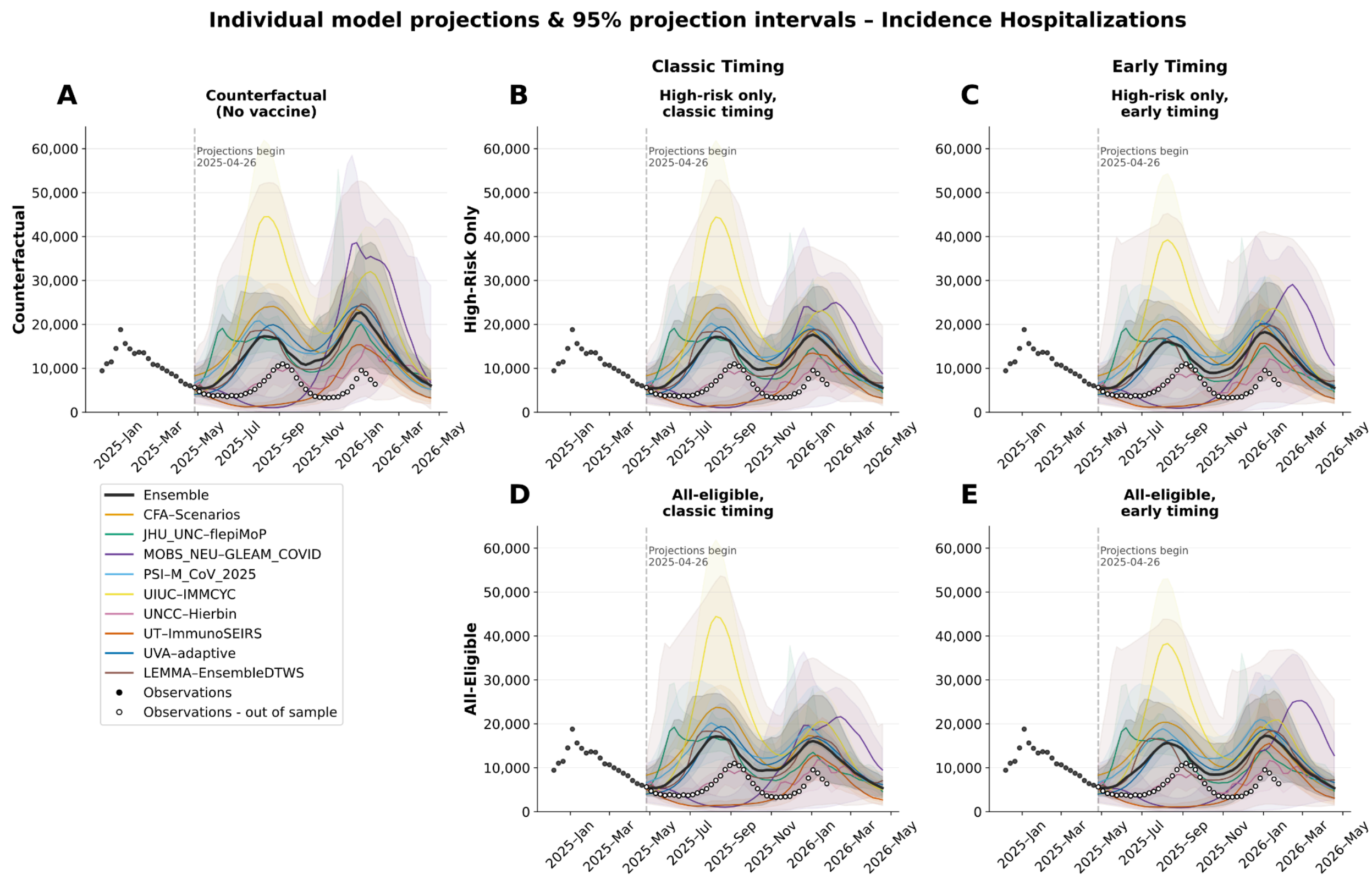

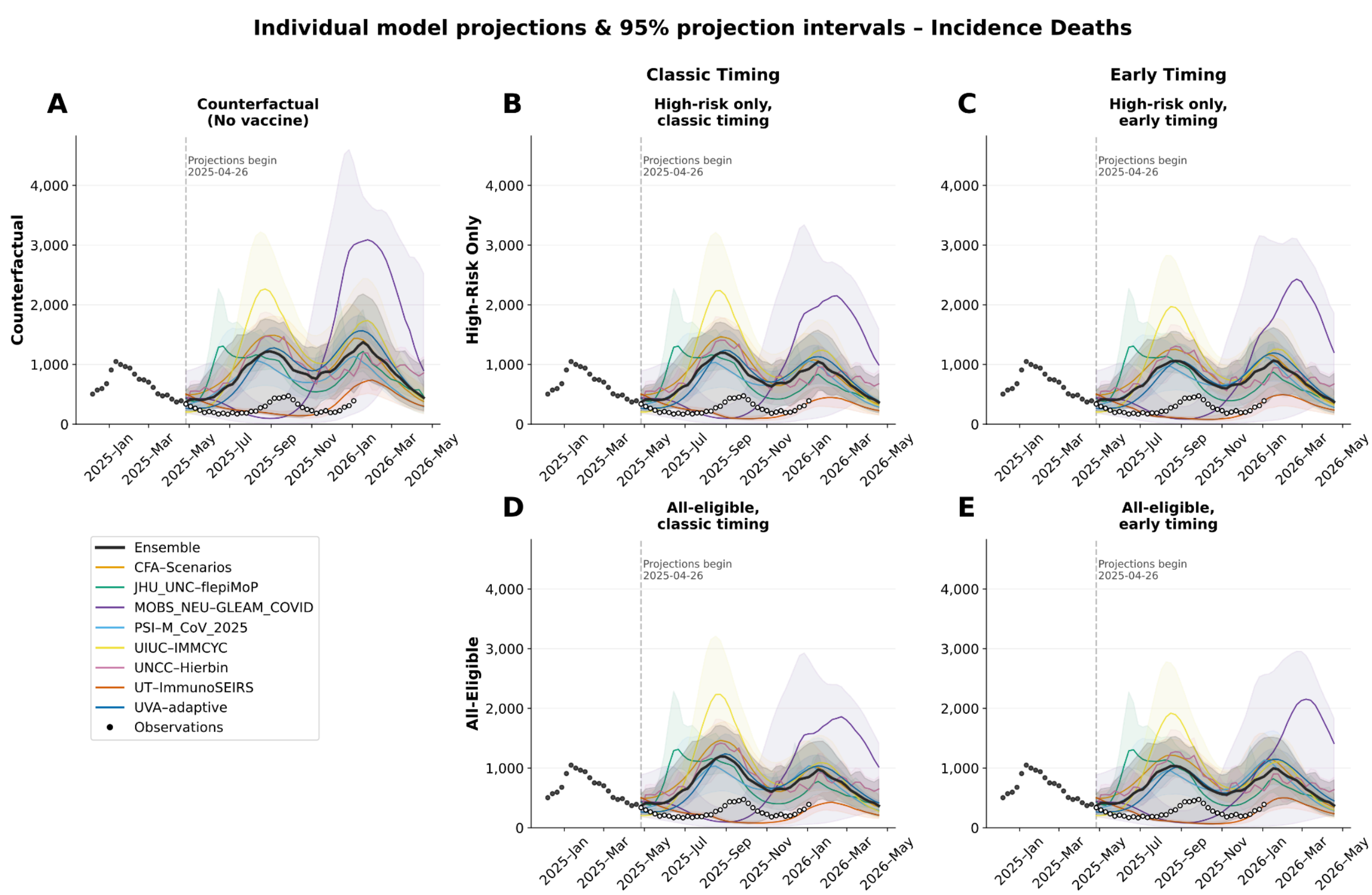

Figure S3. National-level individual-model projections of A) incident hospitalizations and B) incident deaths across scenarios.

medRxiv preprint doi: https://doi.org/10.64898/2026.04.07.26349269; this version posted April 8, 2026. The copyright holder for this preprint (which was not certified by peer review) is the author/funder, who has granted medRxiv a license to display the preprint in perpetuity.
It is made available under a CC-BY 4.0 International license .

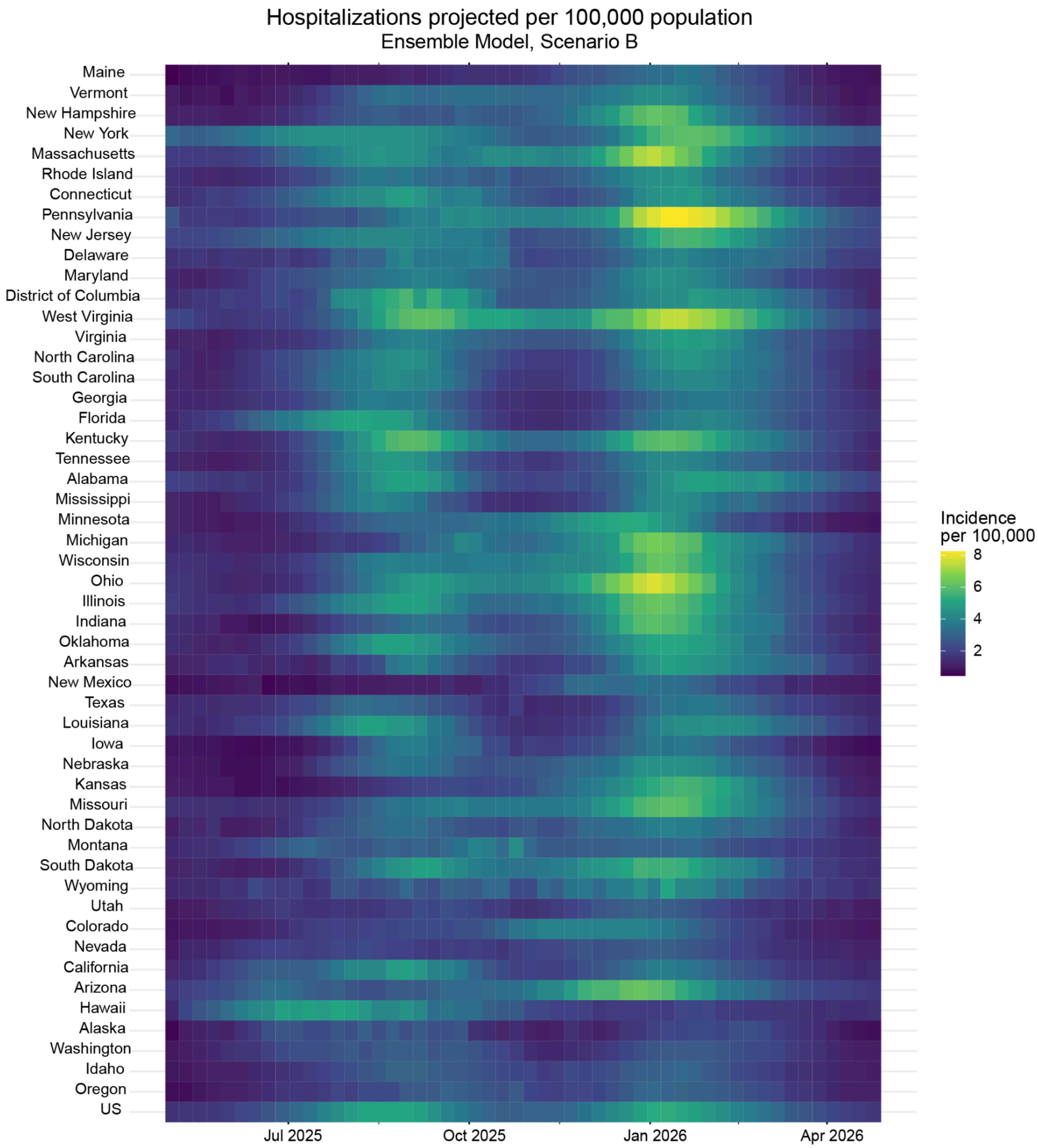

Figure S4. Median projected incident hospitalizations per 100,000 population for Scenario B, by US states in the ensemble model over the projection period 27 April 2025 to 25 April 2026.

medRxiv preprint doi: https://doi.org/10.64898/2026.04.07.26349269; this version posted April 8, 2026. The copyright holder for this preprint (which was not certified by peer review) is the author/funder, who has granted medRxiv a license to display the preprint in perpetuity.
It is made available under a CC-BY 4.0 International license .

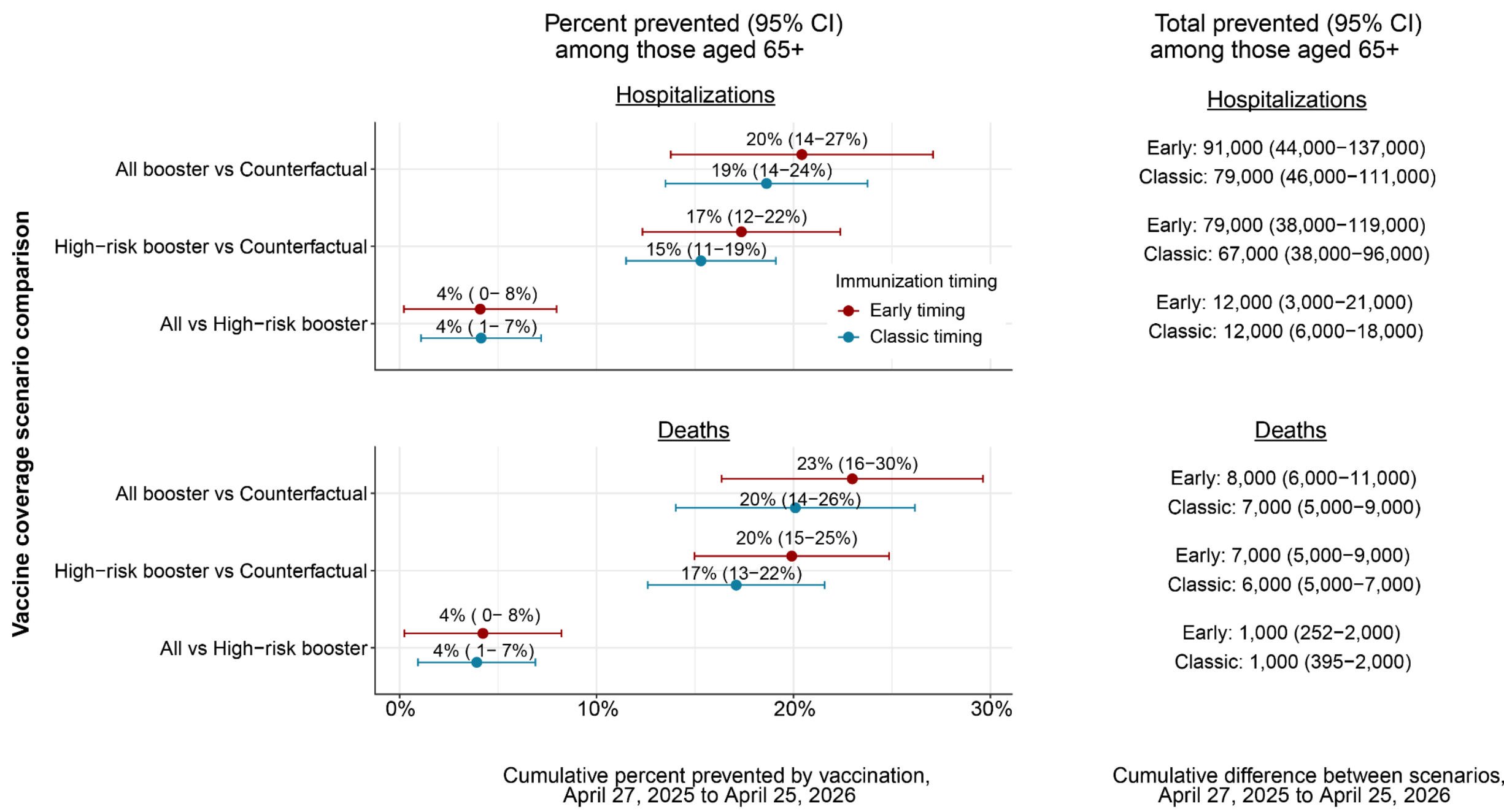

Figure S5. National-level impacts of vaccination coverage on hospitalizations and deaths across the projection period April 27, 2025 to April 25, 2026 for individuals aged 65 and over.

medRxiv preprint doi: https://doi.org/10.64898/2026.04.07.26349269; this version posted April 8, 2026. The copyright holder for this preprint (which was not certified by peer review) is the author/funder, who has granted medRxiv a license to display the preprint in perpetuity.
It is made available under a CC-BY 4.0 International license .

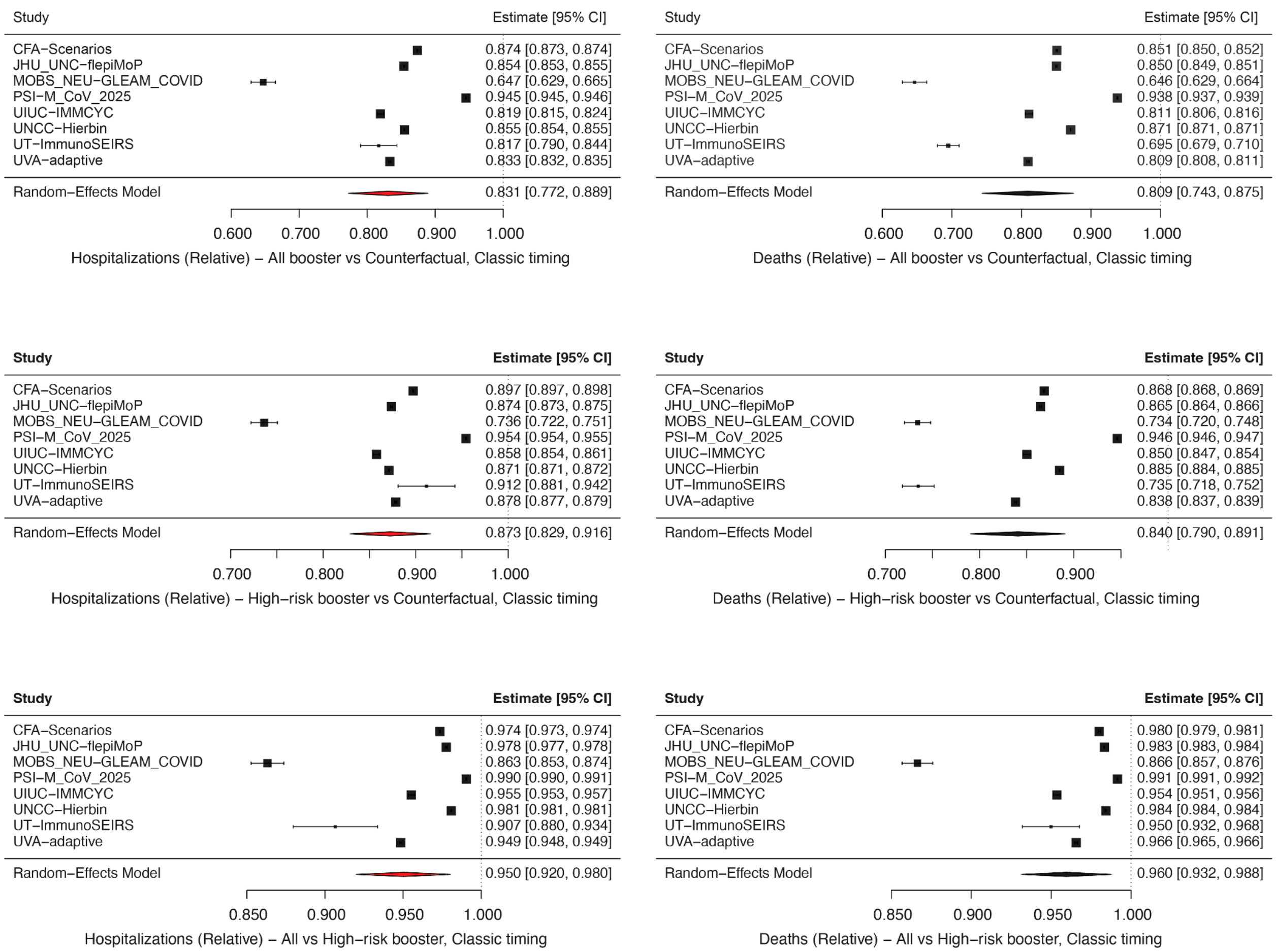

Figure S6. Relative differences between vaccination coverage scenarios by individual model, classic vaccination timing, for all age-groups, compared to the counterfactual (no vaccine recommendation) scenario across the projection period April 27, 2025 to April 25, 2026, United States.

medRxiv preprint doi: https://doi.org/10.64898/2026.04.07.26349269; this version posted April 8, 2026. The copyright holder for this preprint (which was not certified by peer review) is the author/funder, who has granted medRxiv a license to display the preprint in perpetuity.
It is made available under a CC-BY 4.0 International license .

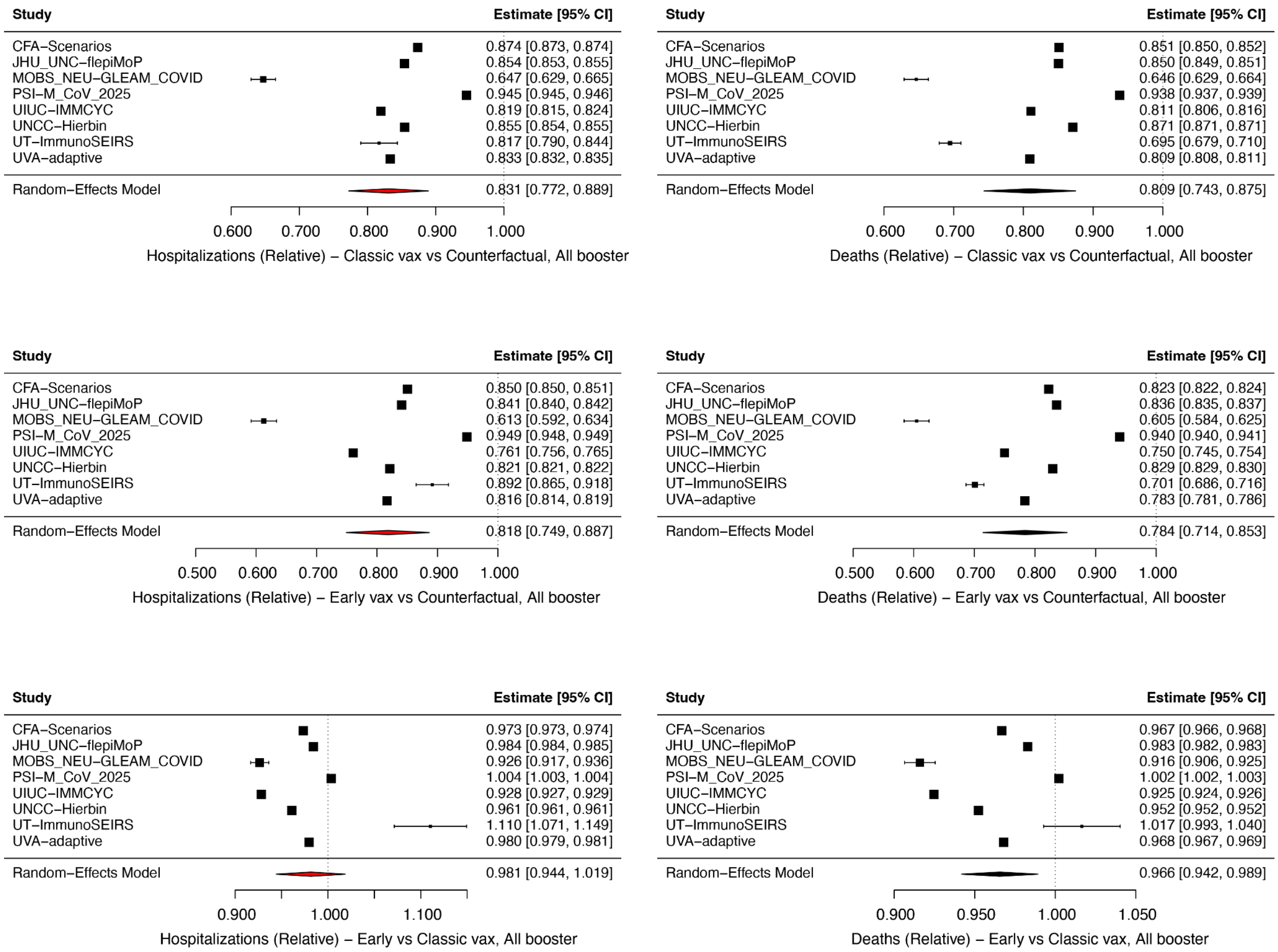

Figure S7. Relative differences between vaccination timing scenarios by individual model, all booster coverage, for all age-groups, compared to the counterfactual (no vaccine recommendation) scenario across the projection period April 27, 2025 to April 25, 2026, United States.

medRxiv preprint doi: https://doi.org/10.64898/2026.04.07.26349269; this version posted April 8, 2026. The copyright holder for this preprint (which was not certified by peer review) is the author/funder, who has granted medRxiv a license to display the preprint in perpetuity.
It is made available under a CC-BY 4.0 International license .

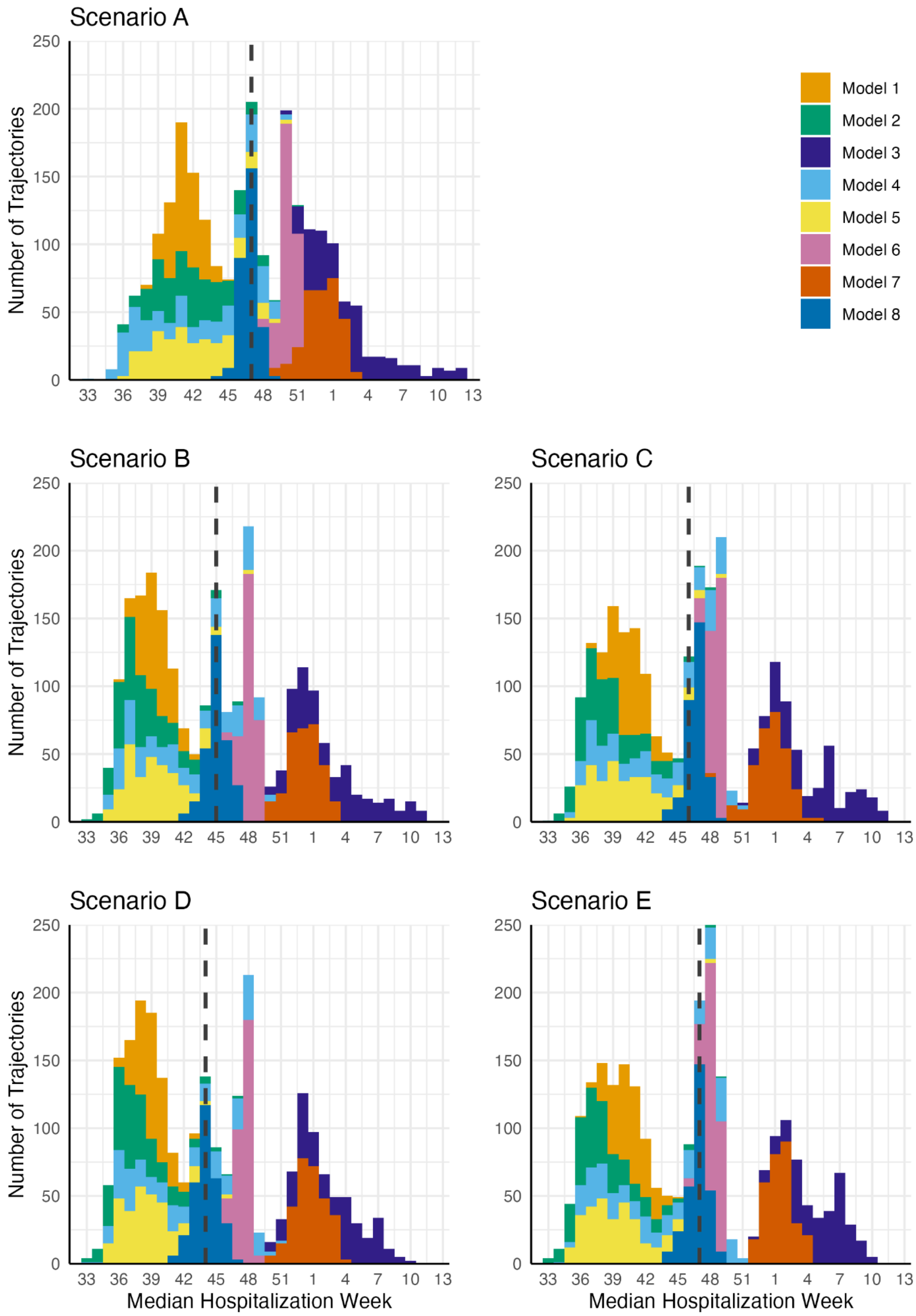

Figure S8. National trajectories distributed by median hospitalization week and colored by model. Vertical dashed lines indicate the median MHW across all models.

medRxiv preprint doi: https://doi.org/10.64898/2026.04.07.26349269; this version posted April 8, 2026. The copyright holder for this preprint (which was not certified by peer review) is the author/funder, who has granted medRxiv a license to display the preprint in perpetuity.
It is made available under a CC-BY 4.0 International license .

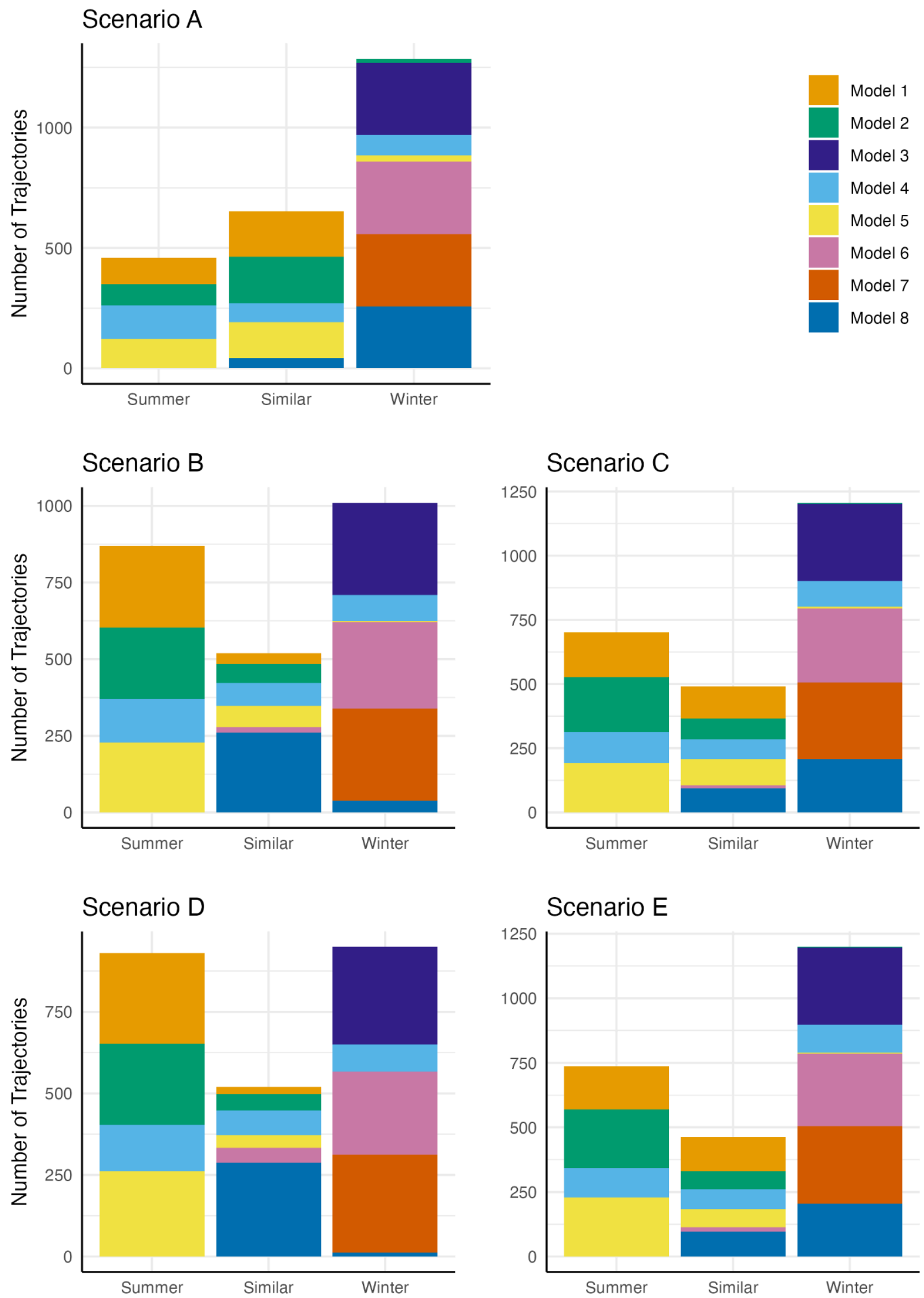

Figure S9. National trajectories characterized by larger summer/winter waves and colored by model. The categories are defined by trajectory cumulative proportion at Epidemic Week 43 (see methods for detail).

medRxiv preprint doi: https://doi.org/10.64898/2026.04.07.26349269; this version posted April 8, 2026. The copyright holder for this preprint (which was not certified by peer review) is the author/funder, who has granted medRxiv a license to display the preprint in perpetuity.
It is made available under a CC-BY 4.0 International license .

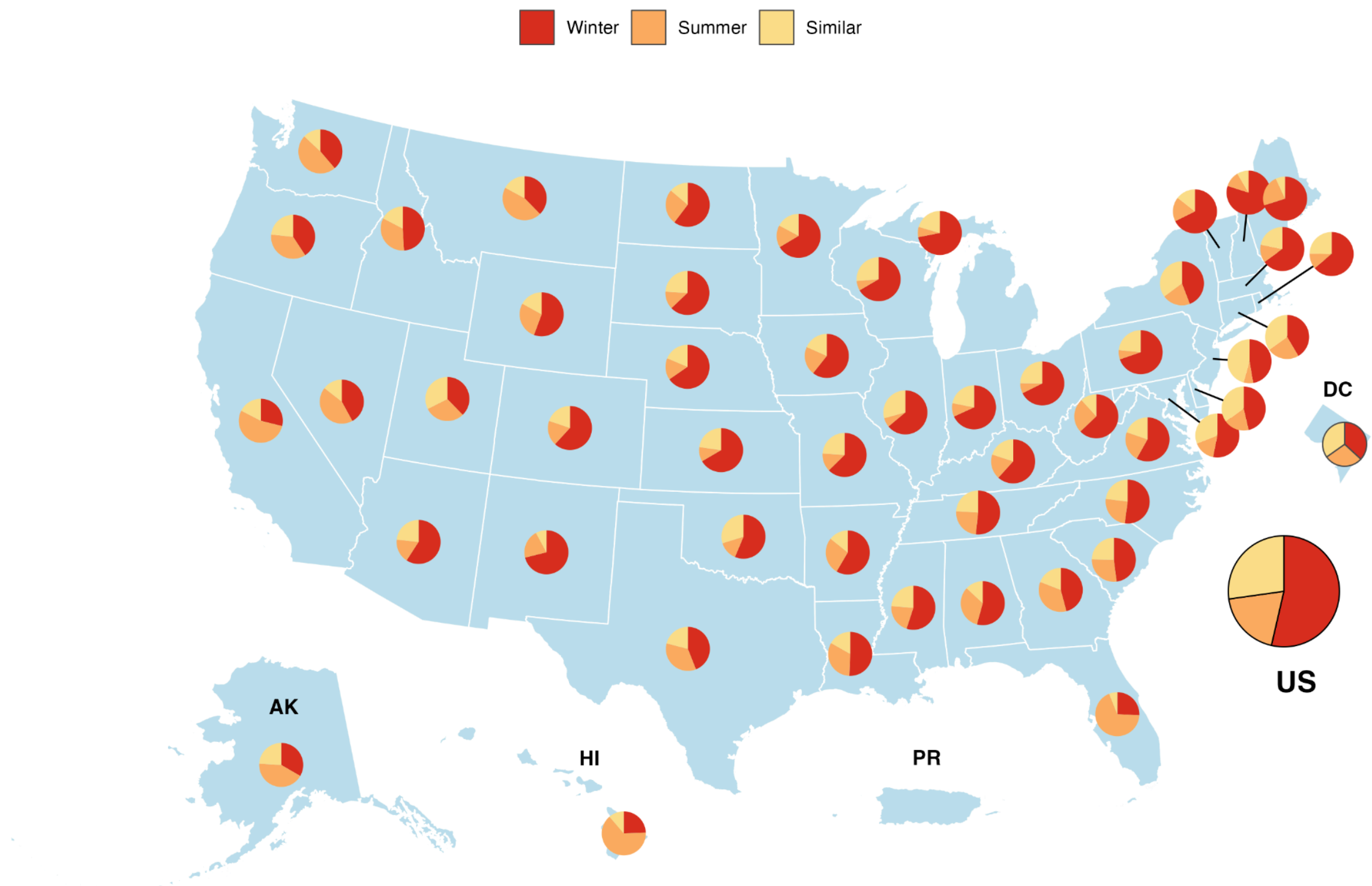

Figure S10. National and location-stratified trajectories for Scenario A, characterized by larger summer/winter waves. The categories are defined by trajectory cumulative proportion at Epidemic Week 43 (see methods for detail).

medRxiv preprint doi: https://doi.org/10.64898/2026.04.07.26349269; this version posted April 8, 2026. The copyright holder for this preprint (which was not certified by peer review) is the author/funder, who has granted medRxiv a license to display the preprint in perpetuity.
It is made available under a CC-BY 4.0 International license .

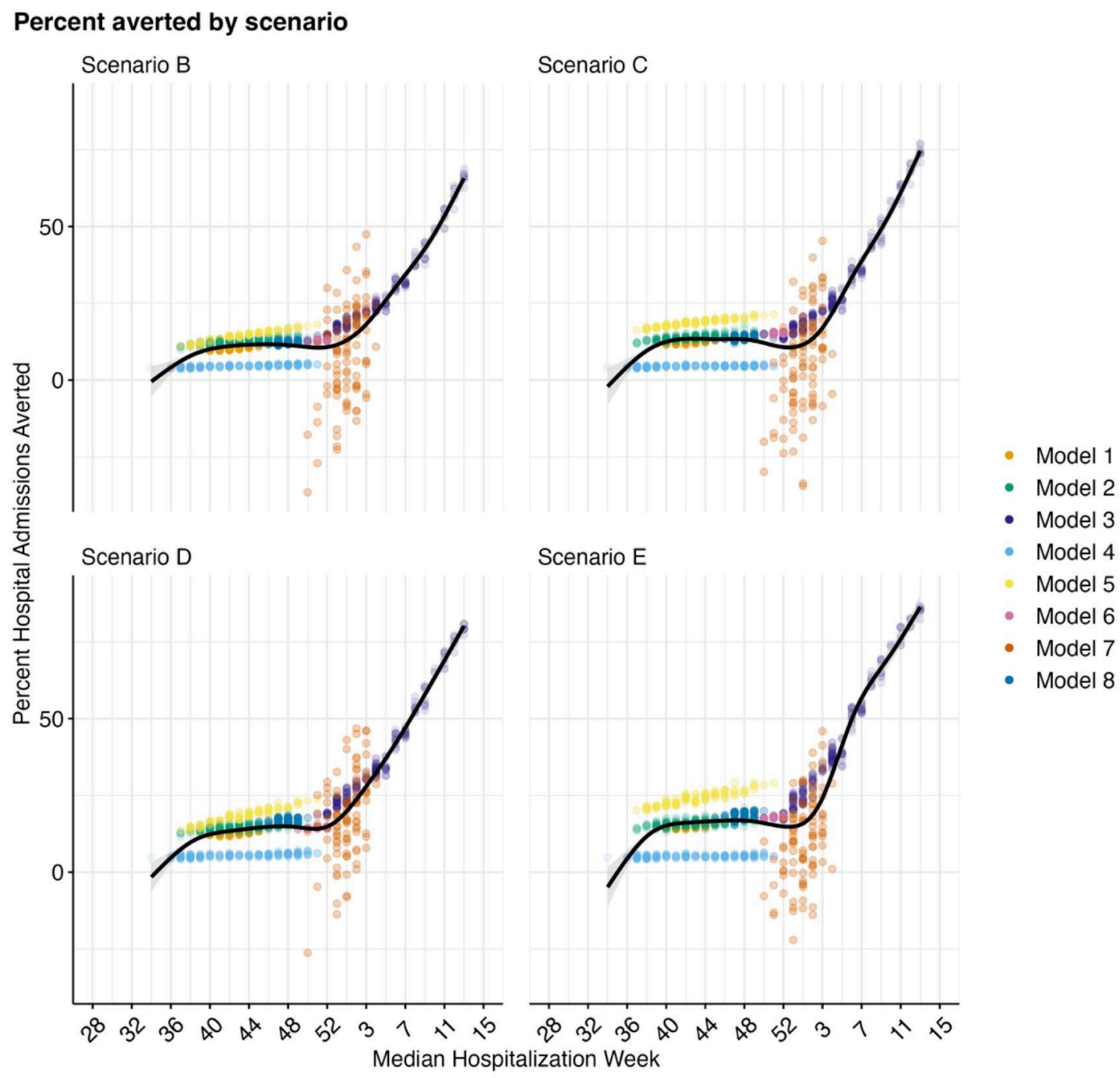

Figure S11. Hospital admissions averted by median hospitalization week at the national level. Points denote individual model trajectories; ensemble regressions (black lines) are generated with a Generalized Additive Model. Median hospitalization week is the epidemic week by which at least 50% of the total hospitalizations have been observed in the paired counterfactual Scenario A.

medRxiv preprint doi: https://doi.org/10.64898/2026.04.07.26349269; this version posted April 8, 2026. The copyright holder for this preprint
(which was not certified by peer review) is the author/funder, who has granted medRxiv a license to display the preprint in perpetuity.
It is made available under a CC-BY 4.0 International license .

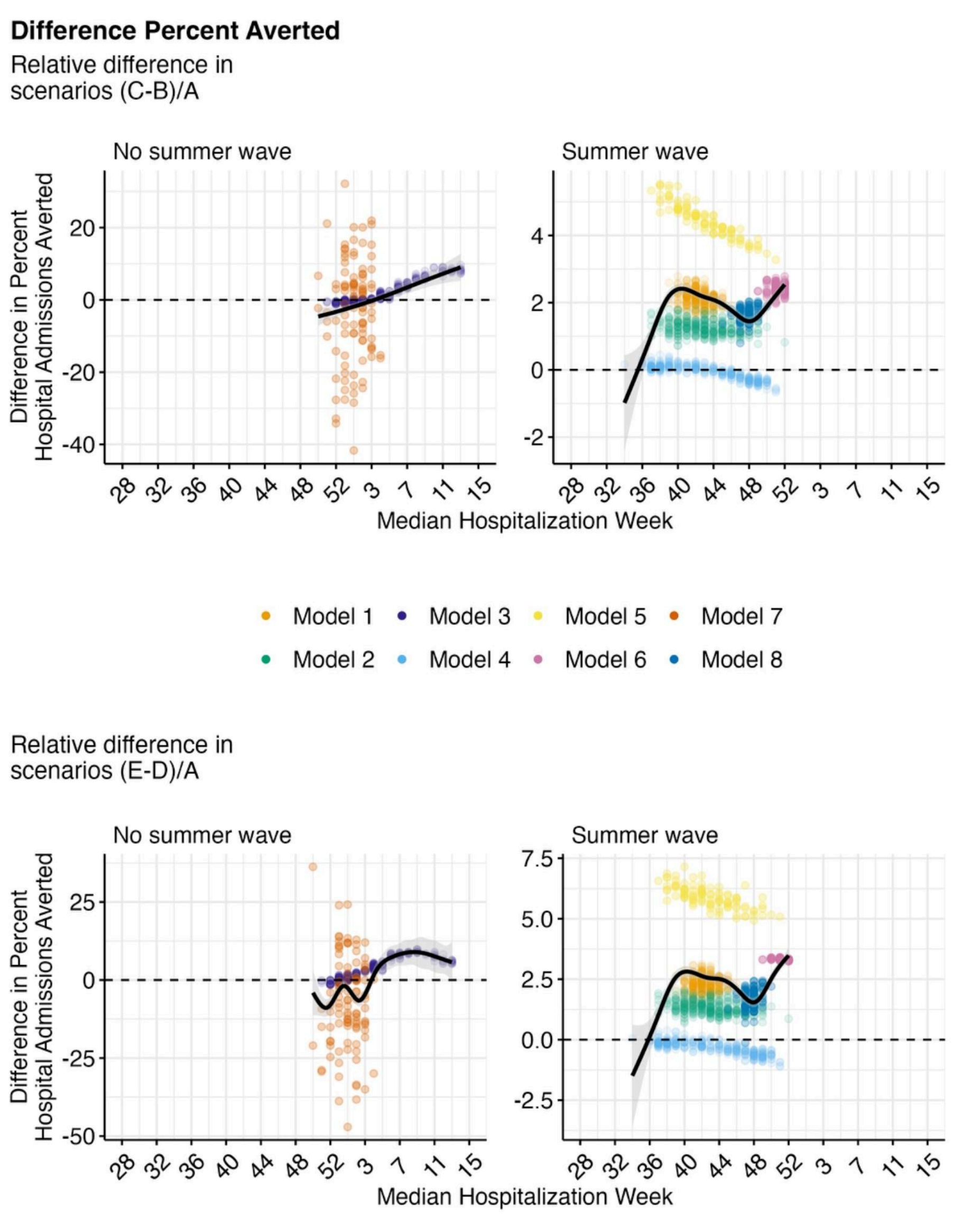

Figure S12. Difference in hospital admissions averted by median hospitalization week at the national level. Points denote individual model trajectories; ensemble regressions (black lines) are generated with a Generalized Additive Model. Dashed lines denote the threshold above which early timing is better and below which classic timing is better. Median hospitalization week is the epidemic week by which at least 50% of the total hospitalizations have been observed in the paired counterfactual Scenario A.

medRxiv preprint doi: https://doi.org/10.64898/2026.04.07.26349269; this version posted April 8, 2026. The copyright holder for this preprint (which was not certified by peer review) is the author/funder, who has granted medRxiv a license to display the preprint in perpetuity.
It is made available under a CC-BY 4.0 International license .

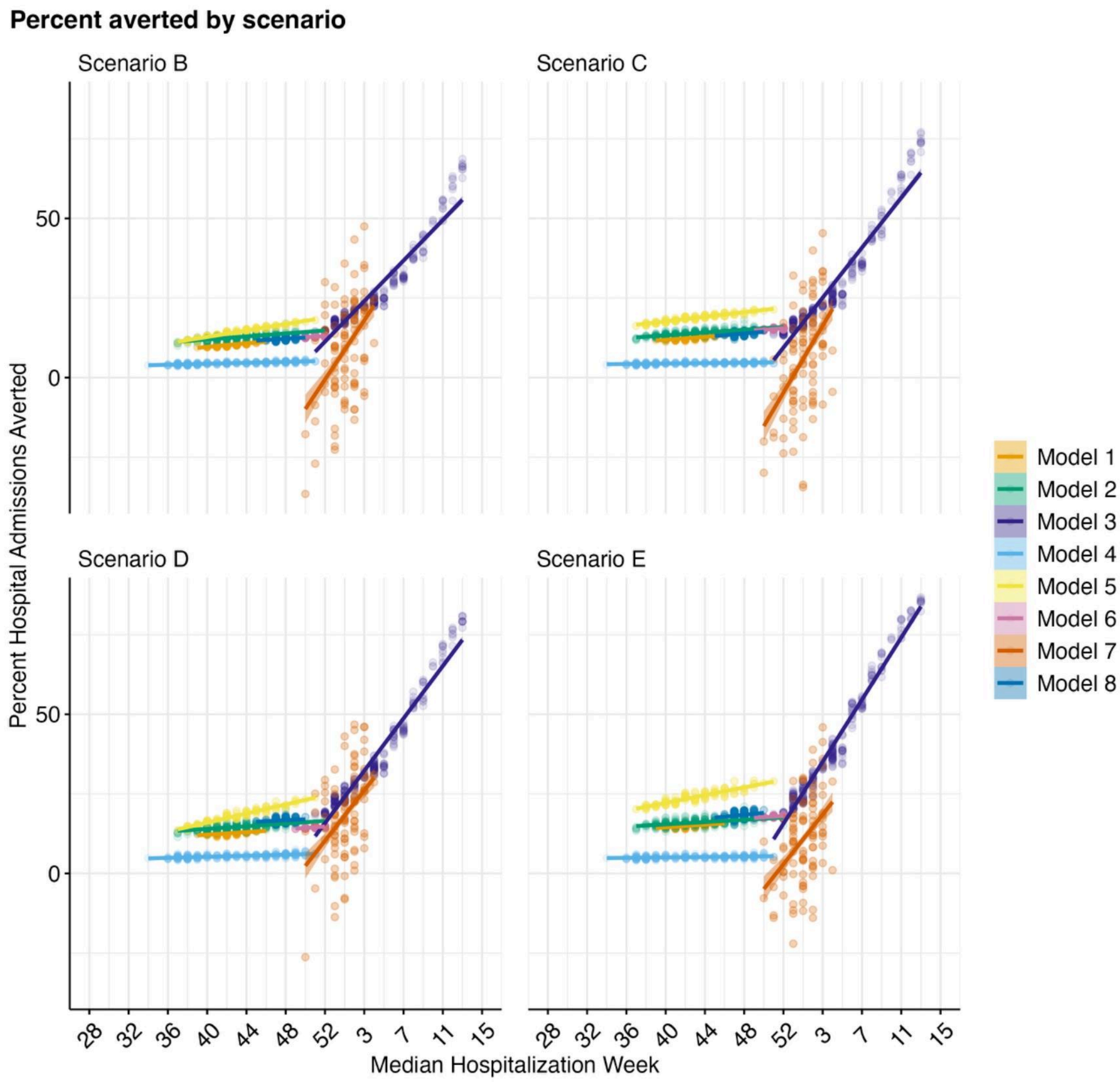

Figure S13. Hospital admissions averted by median hospitalization week at the national level, colored by model. Points denote individual model trajectories. Median hospitalization week is the epidemic week by which at least 50% of the total hospitalizations have been observed in the paired counterfactual Scenario A.

medRxiv preprint doi: https://doi.org/10.64898/2026.04.07.26349269; this version posted April 8, 2026. The copyright holder for this preprint (which was not certified by peer review) is the author/funder, who has granted medRxiv a license to display the preprint in perpetuity.
It is made available under a CC-BY 4.0 International license .

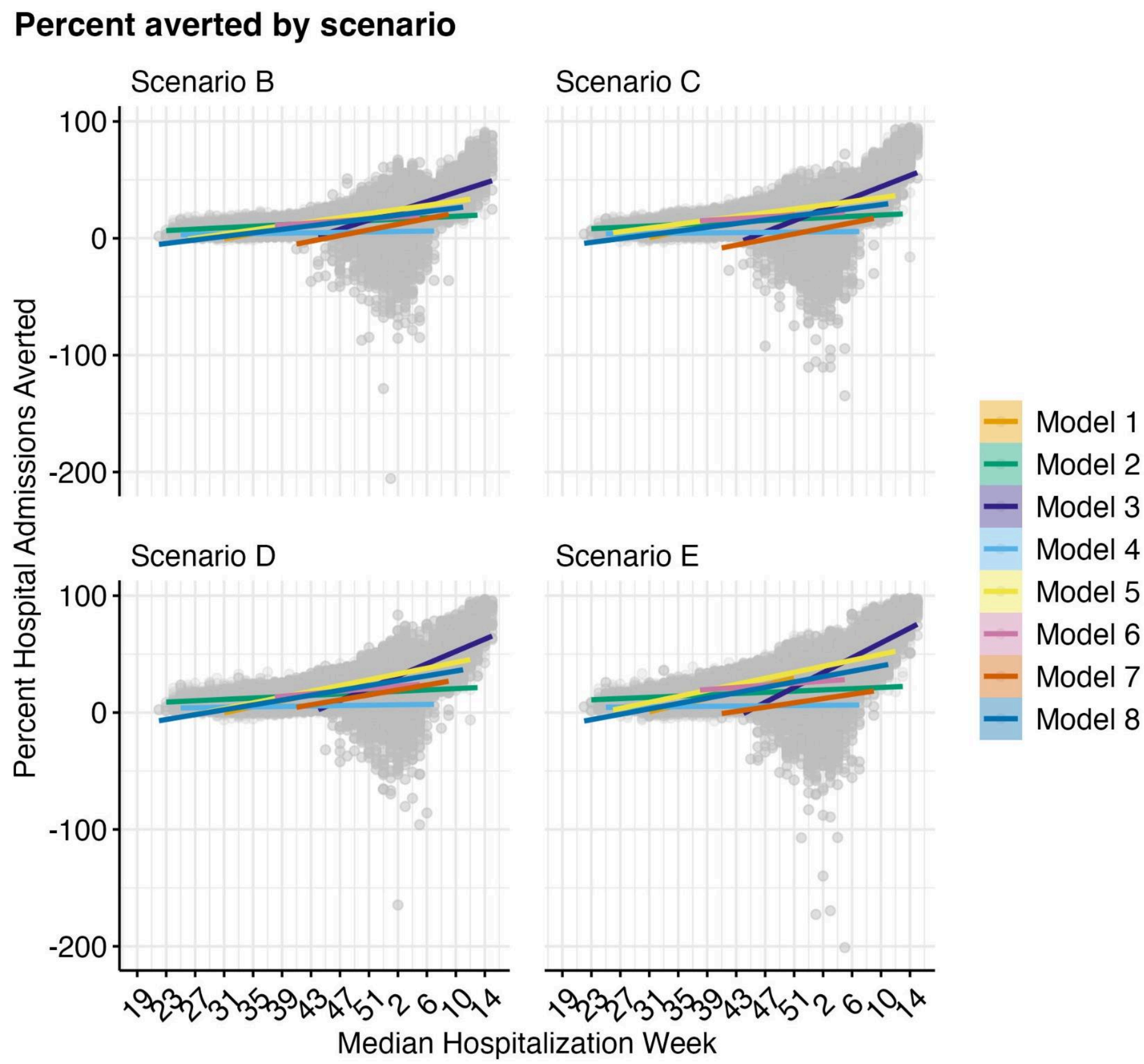

Figure S14. Hospital admissions averted by median hospitalization week across all subnational locations, colored by model. Points denote individual model trajectories. Median hospitalization week is the epidemic week by which at least 50% of the total hospitalizations have been observed in the paired counterfactual Scenario A.

medRxiv preprint doi: https://doi.org/10.64898/2026.04.07.26349269; this version posted April 8, 2026. The copyright holder for this preprint (which was not certified by peer review) is the author/funder, who has granted medRxiv a license to display the preprint in perpetuity.
It is made available under a CC-BY 4.0 International license .

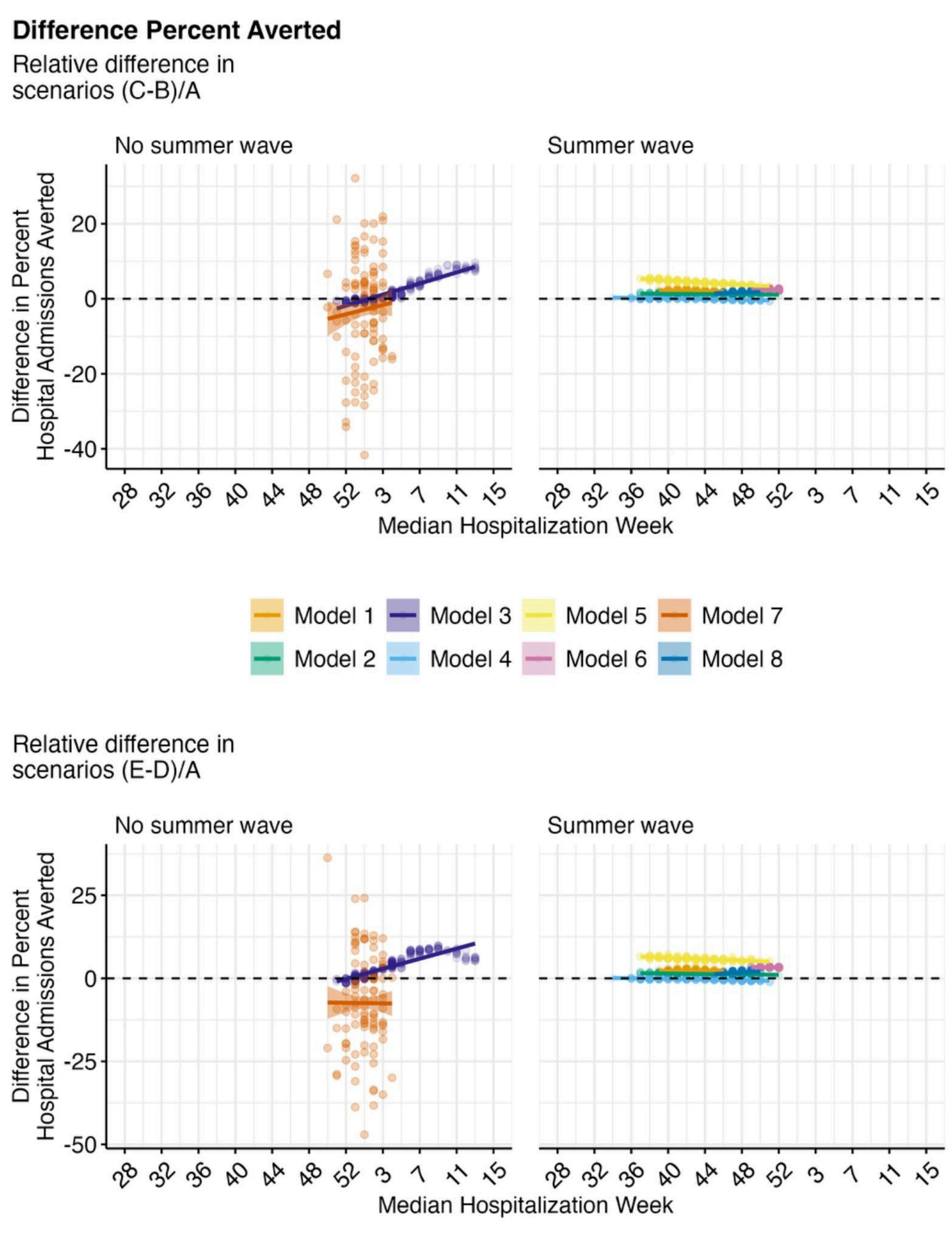

Figure S15. Hospital admissions averted by median hospitalization week, comparing scenarios C/B and E/D at the national level, colored by model. Points denote individual model trajectories. Dashed lines denote the threshold above which early timing is better and below which classic timing is better. Models are grouped by whether they project a summer wave. Median hospitalization week is the epidemic week by which at least 50% of the total hospitalizations have been observed in the paired counterfactual scenario (A).

medRxiv preprint doi: https://doi.org/10.64898/2026.04.07.26349269; this version posted April 8, 2026. The copyright holder for this preprint (which was not certified by peer review) is the author/funder, who has granted medRxiv a license to display the preprint in perpetuity.
It is made available under a CC-BY 4.0 International license .

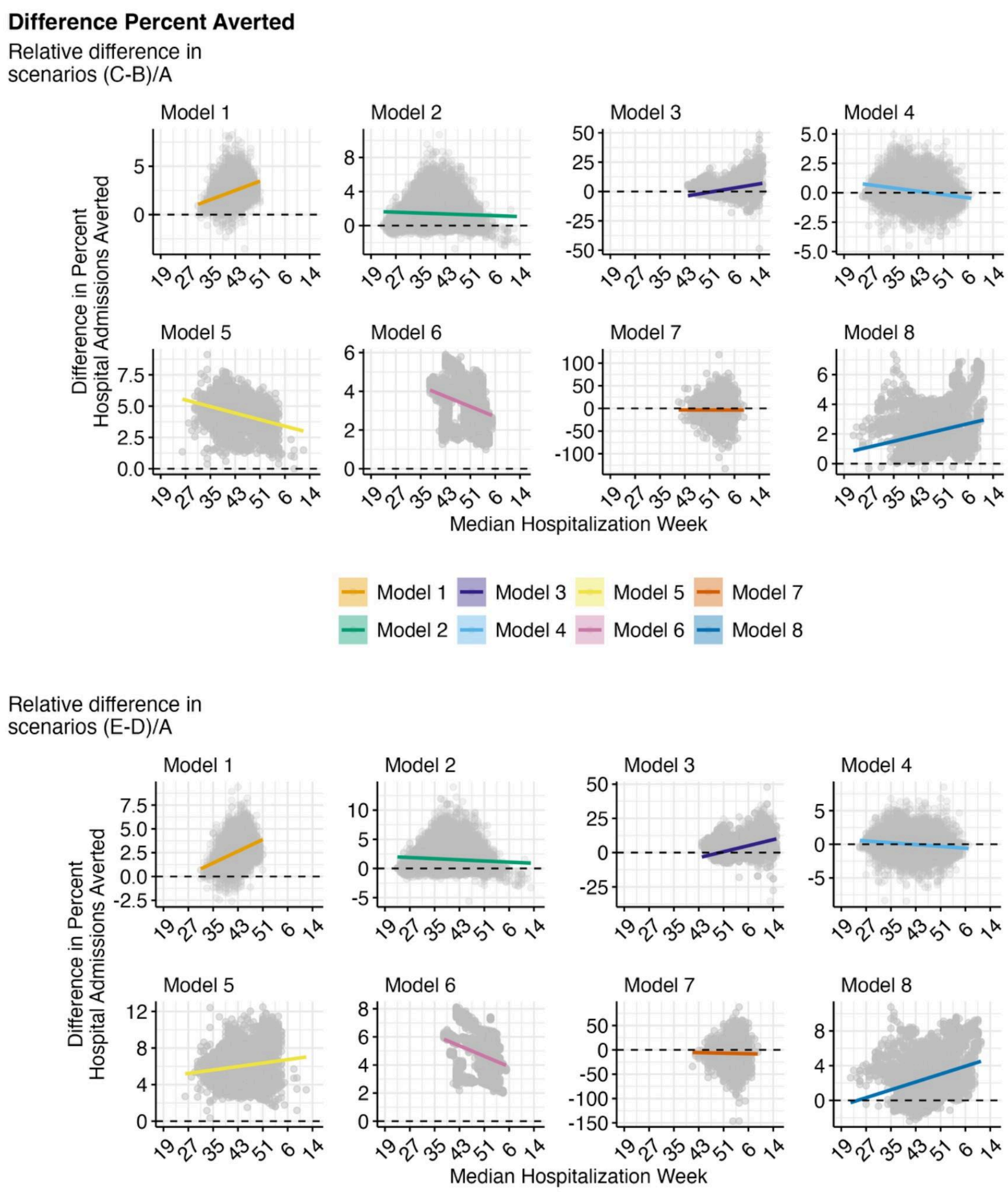

Figure S16. Hospital admissions averted by median hospitalization week across all states, as the difference between scenarios C/B and E/D. Points denote individual model trajectories. Dashed lines denote the threshold above which early timing is better and below which classic timing is better. Models are grouped by whether they project a summer wave. Median hospitalization week is the epidemic week by which at least 50% of the total hospitalizations have been observed in the paired counterfactual scenario (A).

medRxiv preprint doi: https://doi.org/10.64898/2026.04.07.26349269; this version posted April 8, 2026. The copyright holder for this preprint (which was not certified by peer review) is the author/funder, who has granted medRxiv a license to display the preprint in perpetuity.
It is made available under a CC-BY 4.0 International license .

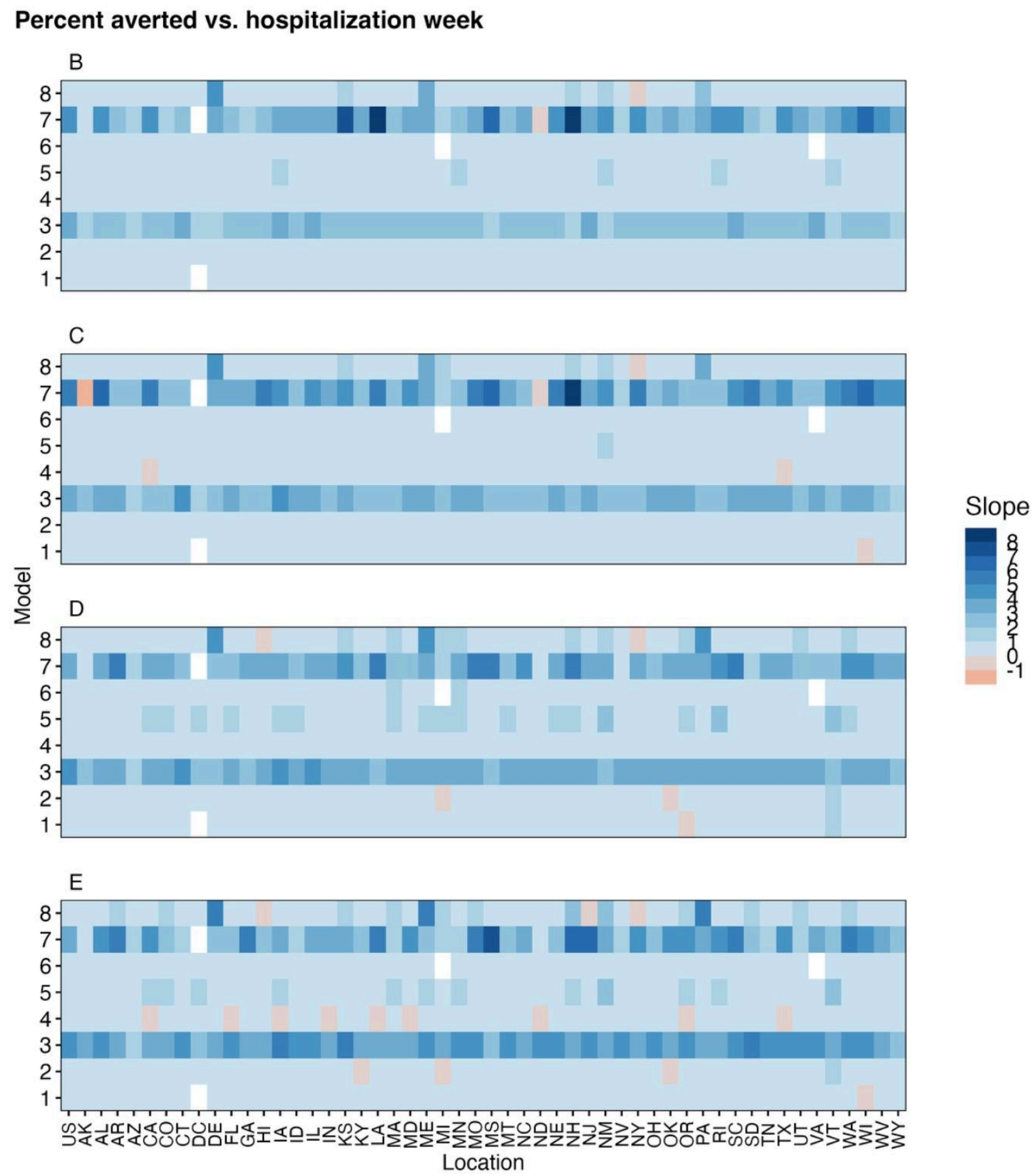

Figure S17. Slope of linear regression comparing percent averted to median hospitalization week, across models and locations. Median hospitalization week is the epidemic week by which at least 50% of the total hospitalizations have been observed in the paired counterfactual scenario (A).

medRxiv preprint doi: https://doi.org/10.64898/2026.04.07.26349269; this version posted April 8, 2026. The copyright holder for this preprint (which was not certified by peer review) is the author/funder, who has granted medRxiv a license to display the preprint in perpetuity.
It is made available under a CC-BY 4.0 International license .

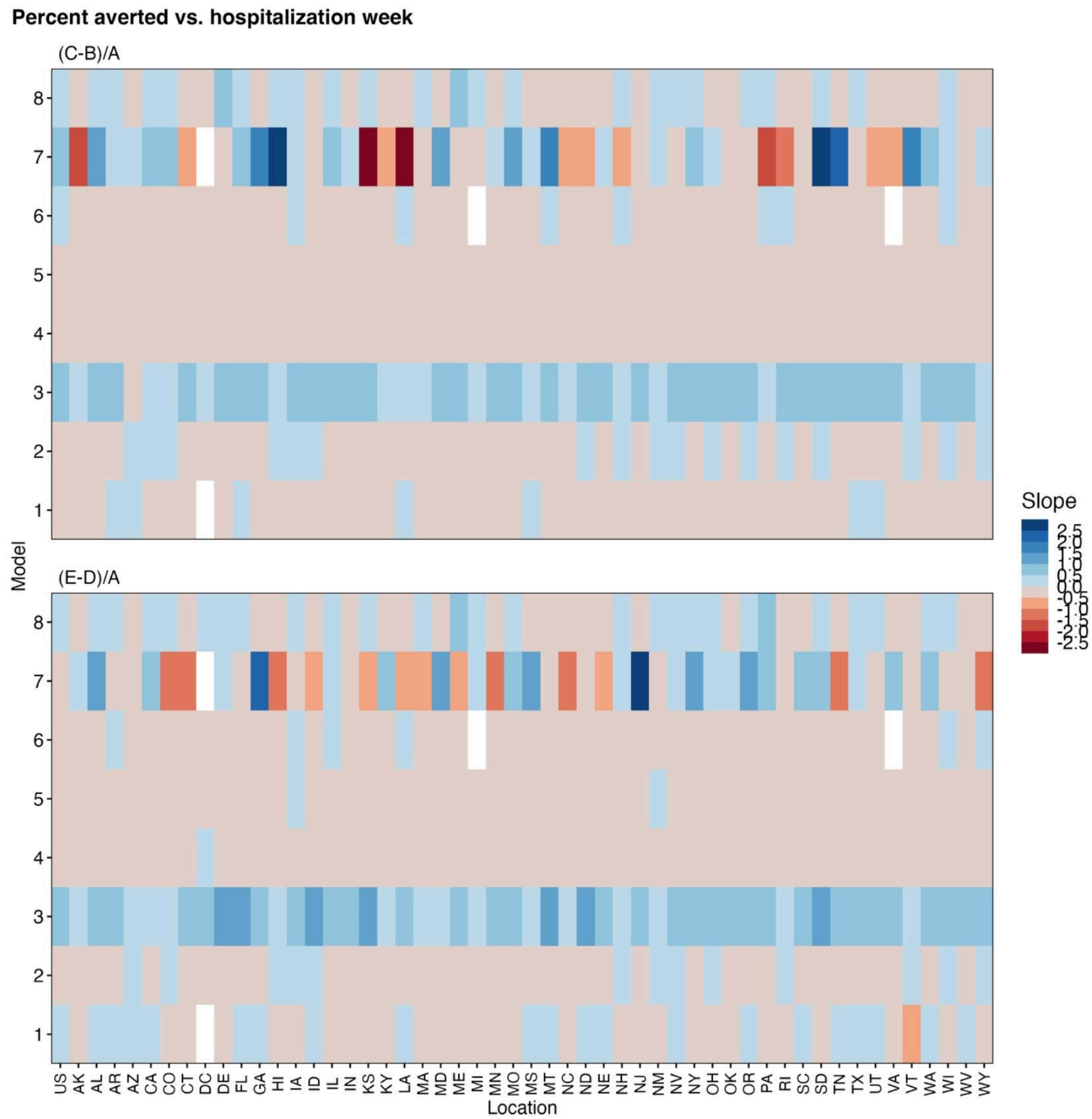

Figure S18. Slope of linear regression comparing difference in percent averted for scenarios C/B and D/E to median hospitalization week across models and locations. Median hospitalization week is the epidemic week by which at least 50% of the total hospitalizations have been observed in the paired counterfactual scenario (A).

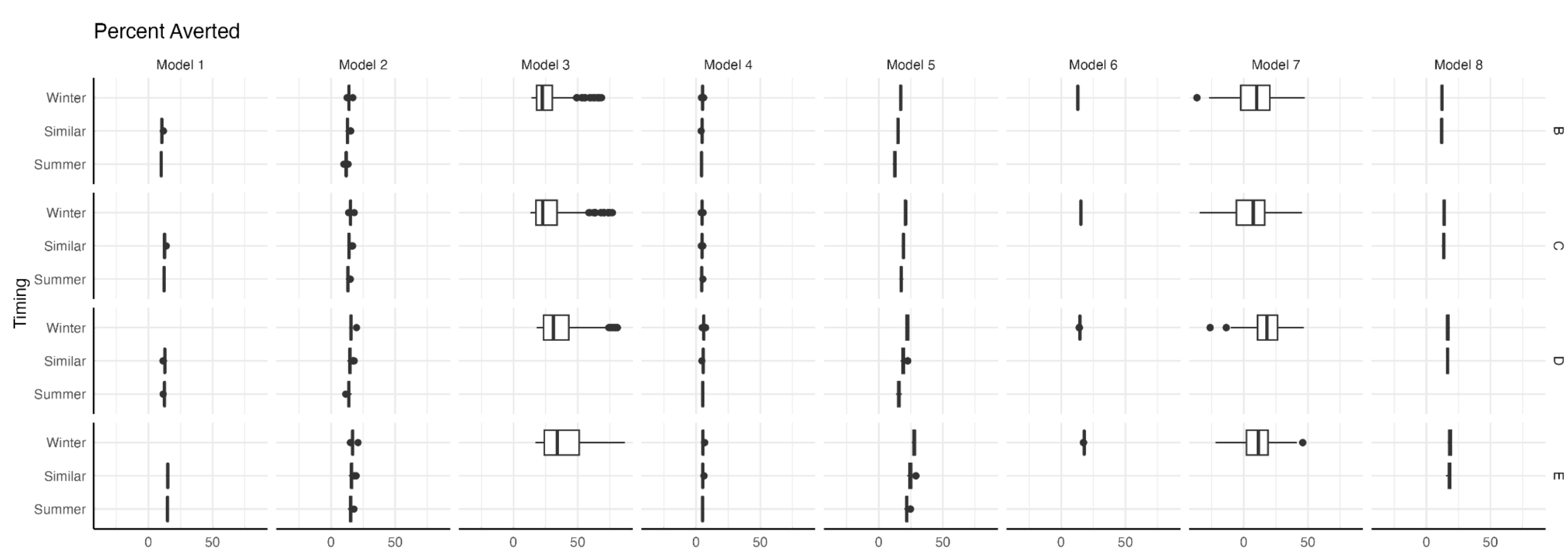

medRxiv preprint doi: https://doi.org/10.64898/2026.04.07.26349269; this version posted April 8, 2026. The copyright holder for this preprint (which was not certified by peer review) is the author/funder, who has granted medRxiv a license to display the preprint in perpetuity.
It is made available under a CC-BY 4.0 International license .

Figure S19. Percent averted by scenario and model, grouped by larger summer/winter wave. The categories are defined by trajectory cumulative proportion at Epidemic Week 43 (see methods for detail). Categories with fewer than 10 unique trajectories are removed.

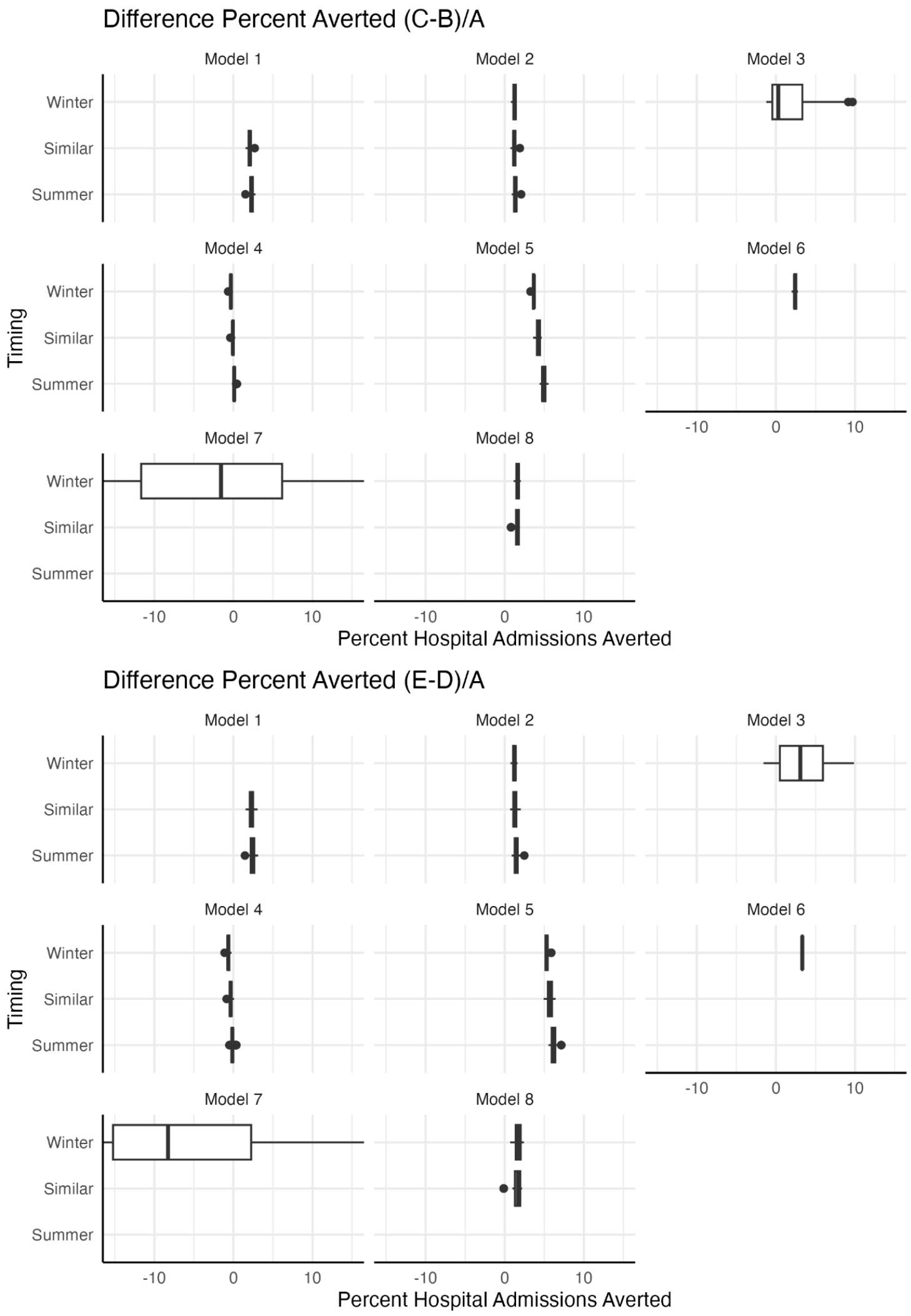

Figure S20. Percent averted comparing C/B and E/D by model, grouped by larger summer/winter wave. The categories are defined by trajectory cumulative proportion at Epidemic Week 43 (see methods for detail). Categories with fewer than 10 unique trajectories are removed.

medRxiv preprint doi: https://doi.org/10.64898/2026.04.07.26349269; this version posted April 8, 2026. The copyright holder for this preprint (which was not certified by peer review) is the author/funder, who has granted medRxiv a license to display the preprint in perpetuity.
It is made available under a CC-BY 4.0 International license .

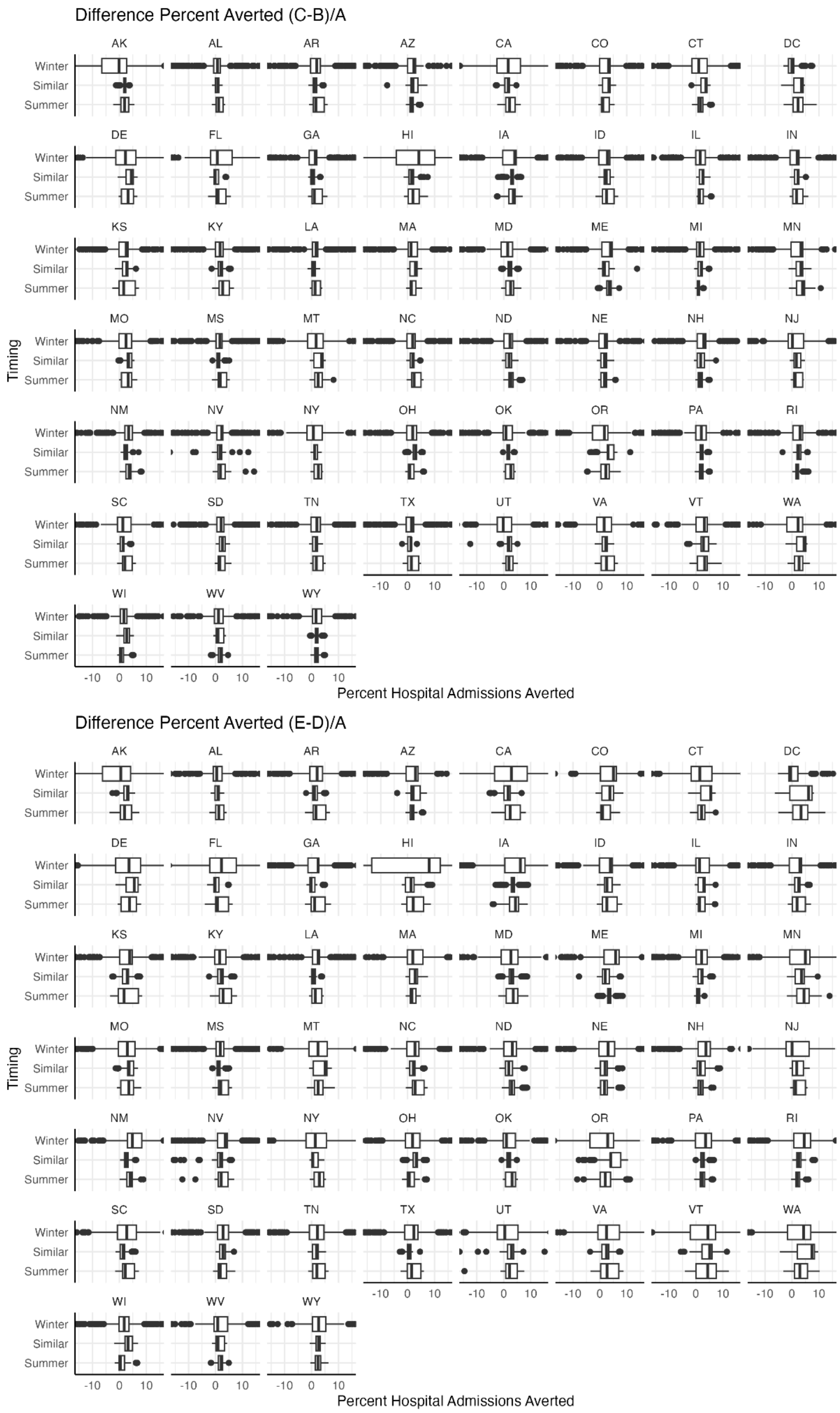

Figure S21. Percent averted comparing C/B and E/D by location, grouped by larger summer/winter wave. The categories are defined by trajectory cumulative proportion at Epidemic Week 43 (see methods for detail).

medRxiv preprint doi: https://doi.org/10.64898/2026.04.07.26349269; this version posted April 8, 2026. The copyright holder for this preprint (which was not certified by peer review) is the author/funder, who has granted medRxiv a license to display the preprint in perpetuity.
It is made available under a CC-BY 4.0 International license .

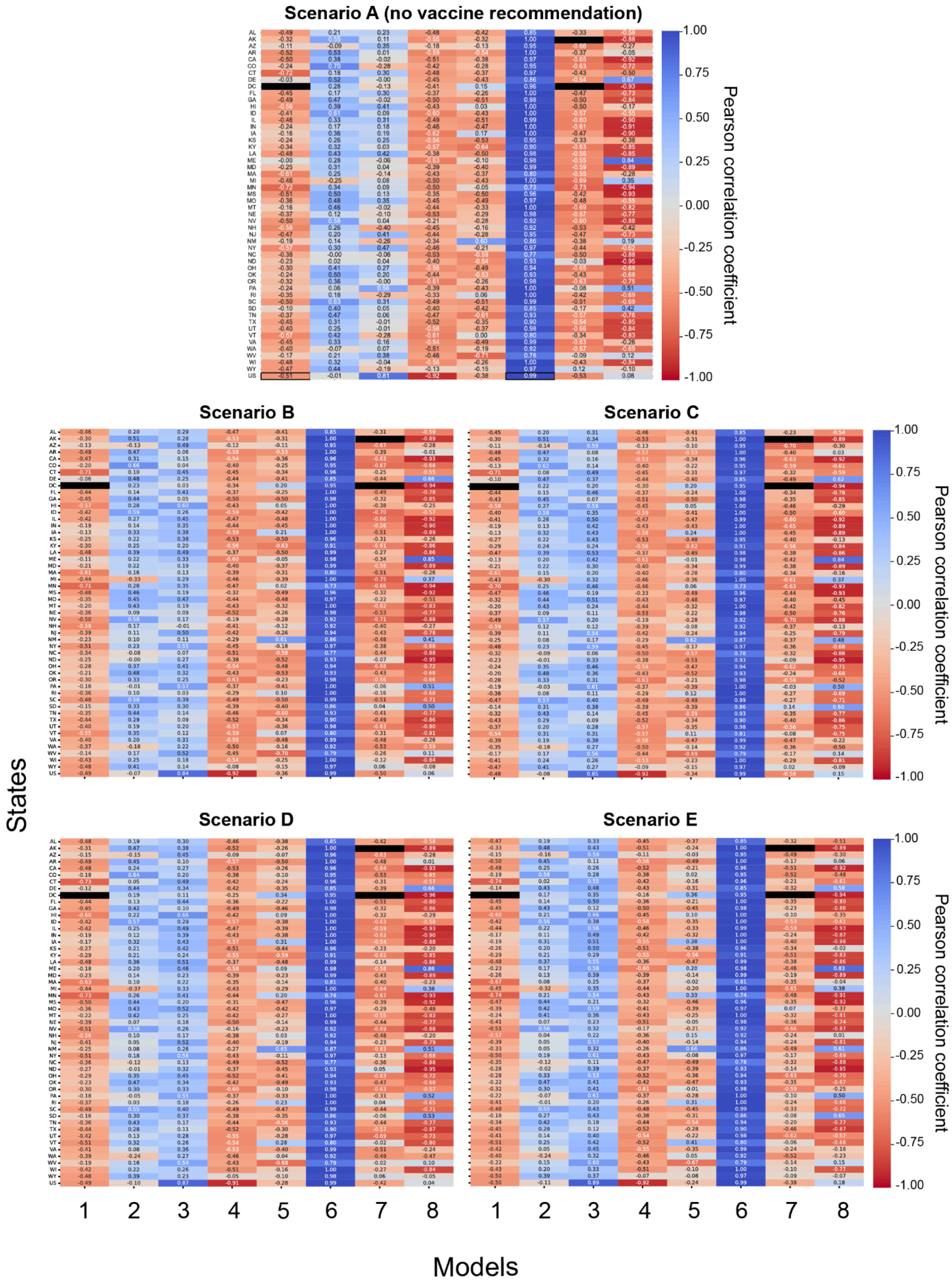

Figure S22. Pearson correlation coefficient *r* between summer and winter wave outbreak sizes for each team and location (state and national-level) for the no vaccine recommendation Scenario A and all vaccine recommendation scenarios B-E over the projection period April 27, 2025 to April 25, 2026. Here wave outbreak size corresponds to the number of hospitalizations per 100,000 individuals. Negative correlation (red) indicates a larger winter wave is associated with a smaller summer wave (example: Model 1), while positive correlation (blue) indicates a larger winter wave is associated with a larger summer wave (example: Model 6). Variation across models implies the

medRxiv preprint doi: https://doi.org/10.64898/2026.04.07.26349269; this version posted April 8, 2026. The copyright holder for this preprint (which was not certified by peer review) is the author/funder, who has granted medRxiv a license to display the preprint in perpetuity.
It is made available under a CC-BY 4.0 International license .

relative sizes of the waves are influenced by different mechanisms within the models. Blacked out cells indicate insufficient data for this analysis.

medRxiv preprint doi: https://doi.org/10.64898/2026.04.07.26349269; this version posted April 8, 2026. The copyright holder for this preprint (which was not certified by peer review) is the author/funder, who has granted medRxiv a license to display the preprint in perpetuity.
It is made available under a CC-BY 4.0 International license .

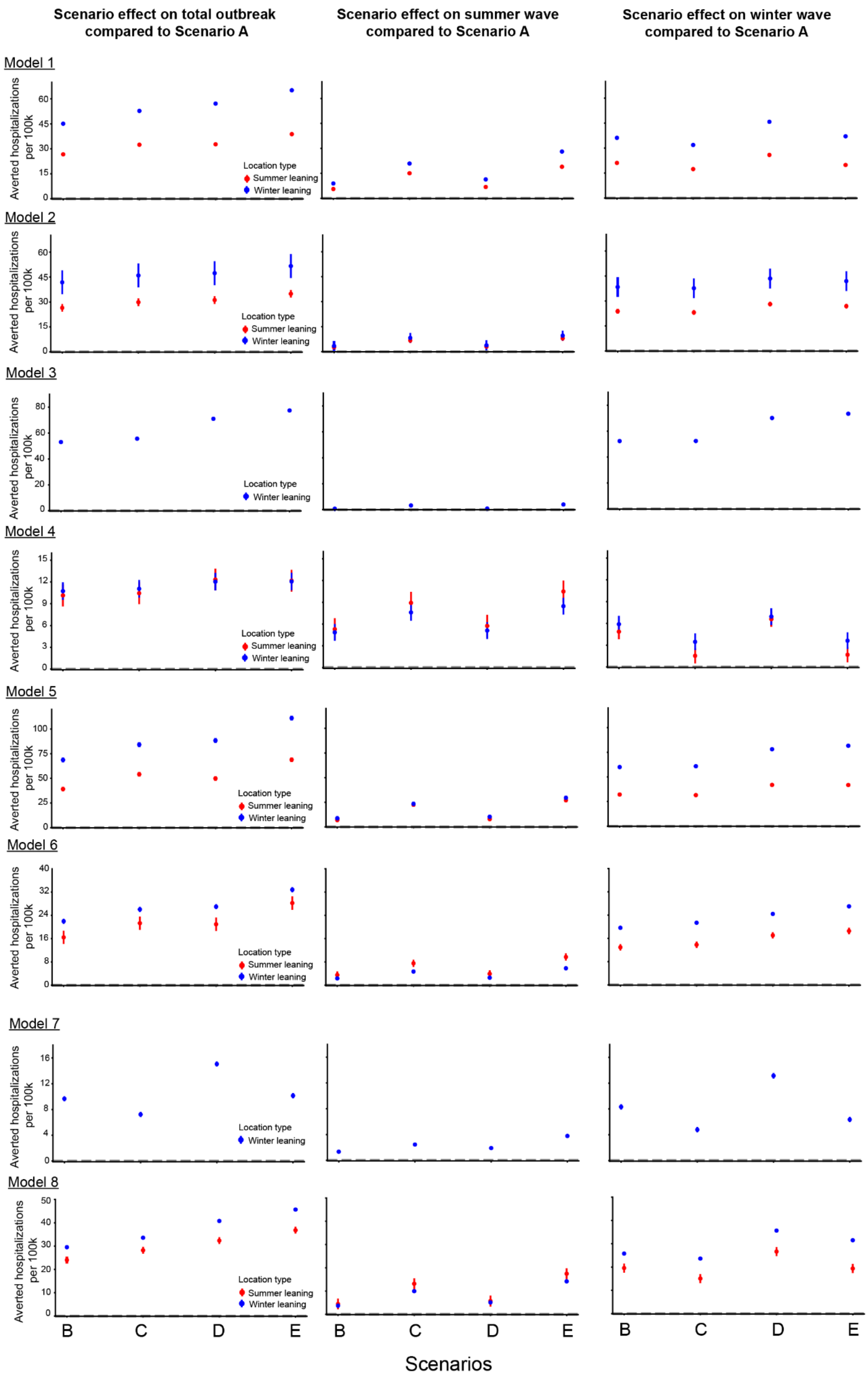

medRxiv preprint doi: https://doi.org/10.64898/2026.04.07.26349269; this version posted April 8, 2026. The copyright holder for this preprint (which was not certified by peer review) is the author/funder, who has granted medRxiv a license to display the preprint in perpetuity.
It is made available under a CC-BY 4.0 International license .

Figure S23. Vaccination scenario effects on the total outbreak, and summer and winter waves per team with scenarios as the fixed effect and location (states) as a random effect over the projection period April 27, 2025 to April 25, 2026. For each model, states are split into summer (red) or winter (blue) leaning based on which wave outbreak size was larger on average across Scenario A trajectories. Scenario effect is shown as the number of hospitalizations averted per 100,000. Error bars correspond to 95% confidence interval.

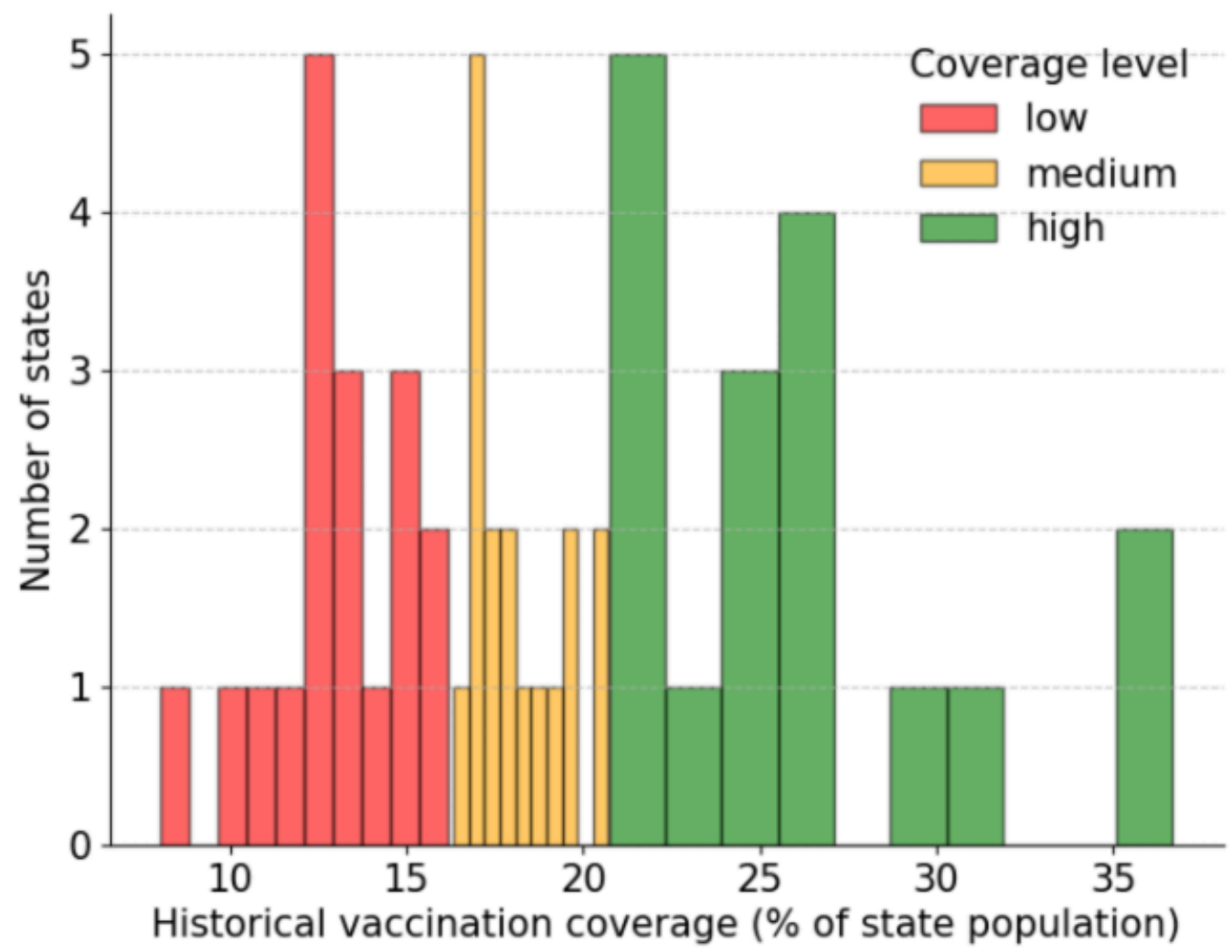

Figure S24. Distribution of states based on their vaccination coverage levels during the 2024-2025 campaign. States were divided into low, medium and high coverage-levels based on tertiles.

medRxiv preprint doi: https://doi.org/10.64898/2026.04.07.26349269; this version posted April 8, 2026. The copyright holder for this preprint (which was not certified by peer review) is the author/funder, who has granted medRxiv a license to display the preprint in perpetuity.
It is made available under a CC-BY 4.0 International license .

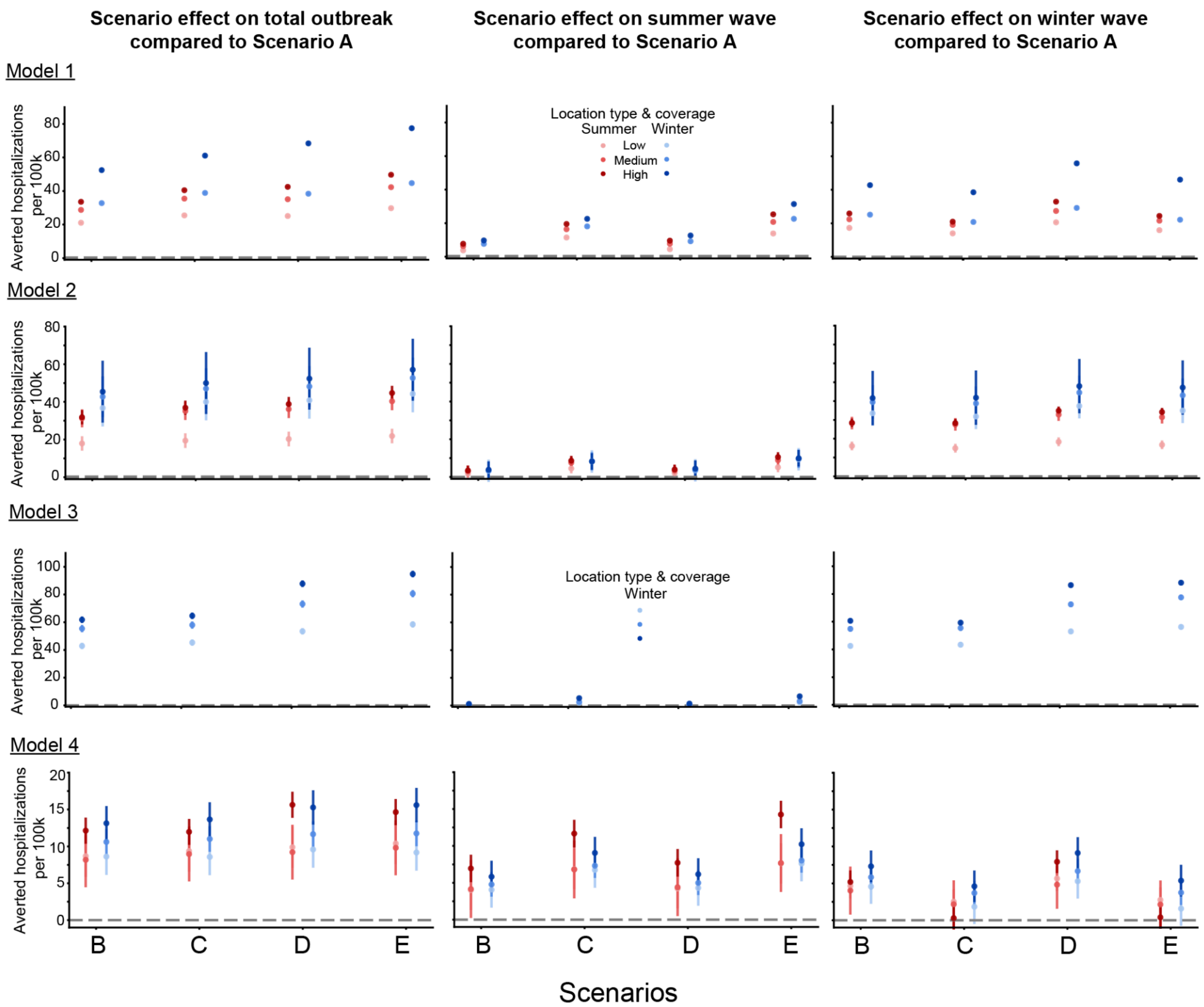

Figure S25. Vaccination scenario effects on the summer and winter wave outbreak sizes for Models 1-4 over the projection period April 27, 2025 to April 25, 2026. States are divided into low, medium and high vaccination coverage levels based on historical coverage levels. For each model, states are further split into summer (red) or winter (blue) leaning based on which wave outbreak size was larger on average across Scenario A trajectories. Scenario effect is expressed as the number of hospitalizations averted per 100,000. Error bars correspond to 95% CI.

medRxiv preprint doi: https://doi.org/10.64898/2026.04.07.26349269; this version posted April 8, 2026. The copyright holder for this preprint (which was not certified by peer review) is the author/funder, who has granted medRxiv a license to display the preprint in perpetuity.
It is made available under a CC-BY 4.0 International license .

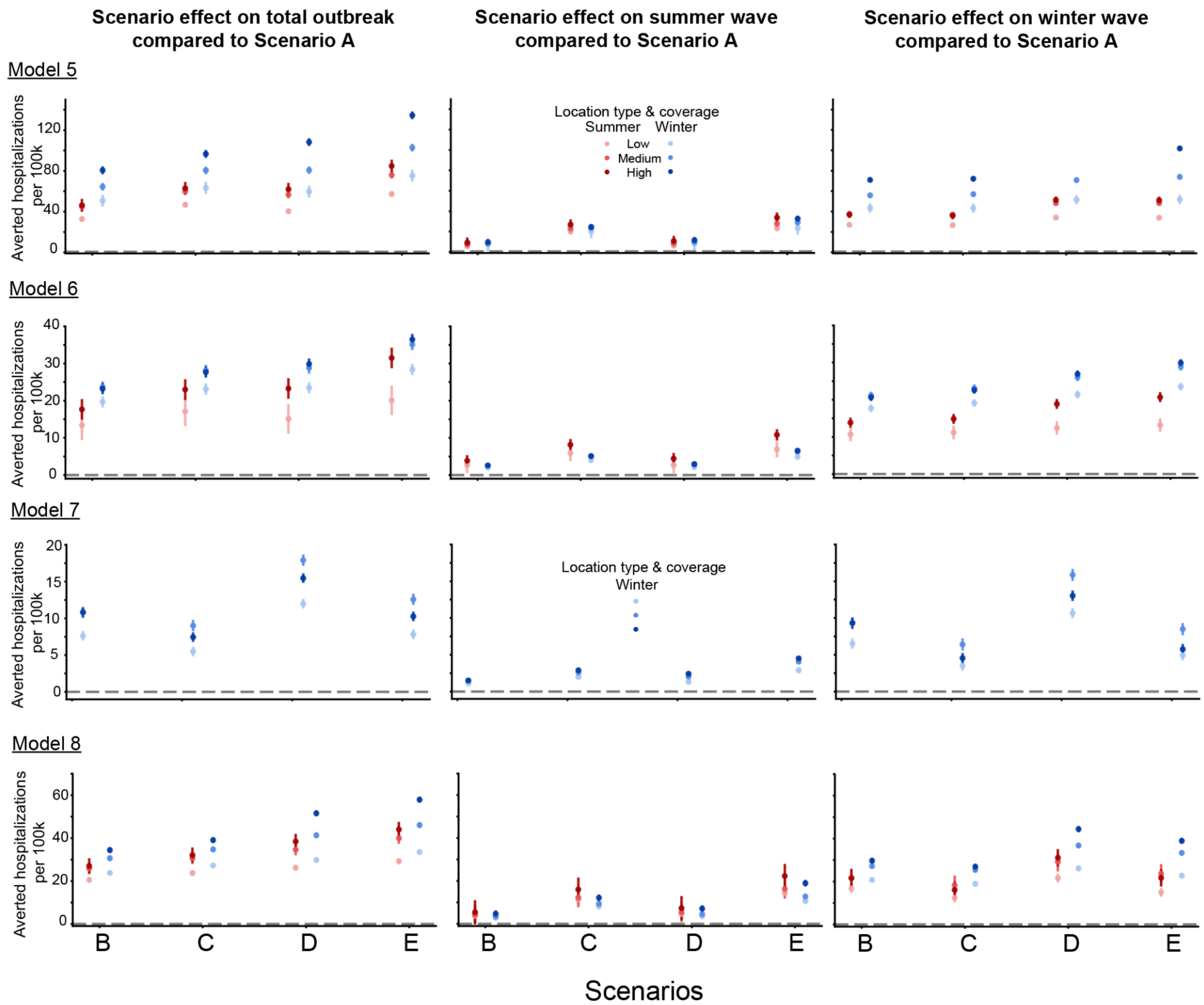

Figure S26. Vaccination scenario effects on the summer and winter wave outbreak sizes for Models 5-8 over the projection period April 27, 2025 to April 25, 2026. States are divided into low, medium and high vaccination coverage levels based on historical coverage levels. For each model, states are further split into summer (red) or winter (blue) leaning based on which wave outbreak size was larger on average across Scenario A trajectories. Scenario effect is expressed as the number of hospitalizations averted per 100,000. Error bars correspond to 95% CI.

medRxiv preprint doi: https://doi.org/10.64898/2026.04.07.26349269; this version posted April 8, 2026. The copyright holder for this preprint (which was not certified by peer review) is the author/funder, who has granted medRxiv a license to display the preprint in perpetuity.
It is made available under a CC-BY 4.0 International license .

4. Supplementary Tables

Model ID	Term	Estimate	Std. Error	t value	Pr(>|t|)	[0.025	0.975]
Model 1	**Intercept**	3.505	0.252	13.900	0.000	3.009	4.001
Model 1	**Slope**	0.278	0.010	26.854	0.000	0.258	0.298
Model 2	**Intercept**	6.789	0.283	23.981	0.000	6.232	7.346
Model 2	**Slope**	0.236	0.011	20.778	0.000	0.213	0.258
Model 3	**Intercept**	-96.896	2.286	-42.392	0.000	-101.394	-92.398
Model 3	**Slope**	3.182	0.059	54.181	0.000	3.067	3.298
Model 4	**Intercept**	2.695	0.089	30.380	0.000	2.520	2.869
Model 4	**Slope**	0.075	0.003	21.394	0.000	0.068	0.082
Model 5	**Intercept**	1.651	0.156	10.552	0.000	1.343	1.959
Model 5	**Slope**	0.505	0.006	81.095	0.000	0.493	0.518
Model 6	**Intercept**	6.520	0.485	13.440	0.000	5.565	7.475
Model 6	**Slope**	0.192	0.015	13.111	0.000	0.163	0.221
Model 7	**Intercept**	-158.483	19.378	-8.178	0.000	-196.619	-120.348
Model 7	**Slope**	4.644	0.537	8.641	0.000	3.586	5.701
Model 8	**Intercept**	6.358	1.032	6.161	0.000	4.327	8.389
Model 8	**Slope**	0.195	0.035	5.621	0.000	0.127	0.263

Table S5. Linear regression results for relative percent averted in Scenario B using the median horizon week of Scenario A as the independent variable.

Model ID	Term	Estimate	Std. Error	t value	Pr(>|t|)	[0.025	0.975]
Model 1	**Intercept**	6.632	0.399	16.628	0.000	5.847	7.417
Model 1	**Slope**	0.237	0.016	14.495	0.000	0.205	0.270

medRxiv preprint doi: https://doi.org/10.64898/2026.04.07.26349269; this version posted April 8, 2026. The copyright holder for this preprint (which was not certified by peer review) is the author/funder, who has granted medRxiv a license to display the preprint in perpetuity.
It is made available under a CC-BY 4.0 International license .

Model 2	**Intercept**	8.468	0.314	26.991	0.000	7.851	9.086
Model 2	**Slope**	0.219	0.013	17.400	0.000	0.194	0.243
Model 3	**Intercept**	-123.950	2.617	-47.358	0.000	-129.101	-118.799
Model 3	**Slope**	3.923	0.067	58.336	0.000	3.791	4.056
Model 4	**Intercept**	3.638	0.090	40.479	0.000	3.461	3.815
Model 4	**Slope**	0.035	0.004	9.792	0.000	0.028	0.042
Model 5	**Intercept**	9.785	0.184	53.172	0.000	9.423	10.147
Model 5	**Slope**	0.358	0.007	48.922	0.000	0.344	0.373
Model 6	**Intercept**	8.853	0.528	16.762	0.000	7.814	9.893
Model 6	**Slope**	0.195	0.016	12.205	0.000	0.163	0.226
Model 7	**Intercept**	-183.548	19.910	-9.219	0.000	-222.729	-144.366
Model 7	**Slope**	5.260	0.552	9.526	0.000	4.173	6.346
Model 8	**Intercept**	6.007	1.377	4.361	0.000	3.296	8.717
Model 8	**Slope**	0.262	0.046	5.657	0.000	0.171	0.353

Table S6. Linear regression results for relative percent averted in Scenario C using the median horizon week of Scenario A as the independent variable.

**Model ID**	**Term**	**Estimate**	**Std. Error**	**t value**	**Pr(>|t|)**	**[0.025**	**0.975]**
Model 1	**Intercept**	7.774	0.389	19.989	0.000	7.008	8.539
Model 1	**Slope**	0.200	0.016	12.522	0.000	0.169	0.231
Model 2	**Intercept**	9.391	0.424	22.142	0.000	8.556	10.226
Model 2	**Slope**	0.209	0.017	12.329	0.000	0.176	0.243
Model 3	**Intercept**	-123.961	2.082	-59.543	0.000	-128.058	-119.864
Model 3	**Slope**	4.111	0.053	76.853	0.000	4.006	4.216
Model 4	**Intercept**	3.411	0.147	23.206	0.000	3.122	3.700
Model 4	**Slope**	0.082	0.006	14.238	0.000	0.071	0.094

medRxiv preprint doi: https://doi.org/10.64898/2026.04.07.26349269; this version posted April 8, 2026. The copyright holder for this preprint (which was not certified by peer review) is the author/funder, who has granted medRxiv a license to display the preprint in perpetuity.
It is made available under a CC-BY 4.0 International license .

Model 5	**Intercept**	0.343	0.311	1.105	0.270	-0.268	0.955
Model 5	**Slope**	0.711	0.012	57.479	0.000	0.687	0.735
Model 6	**Intercept**	7.083	0.427	16.587	0.000	6.243	7.924
Model 6	**Slope**	0.225	0.013	17.483	0.000	0.200	0.251
Model 7	**Intercept**	-124.03	17.054	-7.273	0.000	-157.592	-90.469
Model 7	**Slope**	3.951	0.473	8.354	0.000	3.020	4.881
Model 8	**Intercept**	11.906	1.703	6.993	0.000	8.555	15.256
Model 8	**Slope**	0.160	0.057	2.799	0.005	0.048	0.273

Table S7. Linear regression results for relative percent averted in Scenario D using the median horizon week of Scenario A as the independent variable.

**Model ID**	**Term**	**Estimate**	**Std. Error**	**t value**	**Pr(>|t|)**	**[0.025**	**0.975**
Model 1	**Intercept**	9.927	0.451	22.025	0.000	9.040	10.814
Model 1	**Slope**	0.207	0.019	11.193	0.000	0.171	0.244
Model 2	**Intercept**	11.496	0.462	24.872	0.000	10.587	12.406
Model 2	**Slope**	0.178	0.019	9.642	0.000	0.142	0.215
Model 3	**Intercept**	-149.950	1.932	-77.633	0.000	-153.751	-146.149
Model 3	**Slope**	4.870	0.050	98.134	0.000	4.773	4.968
Model 4	**Intercept**	4.271	0.144	29.685	0.000	3.988	4.555
Model 4	**Slope**	0.035	0.006	6.134	0.000	0.024	0.046
Model 5	**Intercept**	8.799	0.407	21.642	0.000	7.999	9.599
Model 5	**Slope**	0.607	0.016	37.527	0.000	0.576	0.639
Model 6	**Intercept**	11.207	0.462	24.235	0.000	10.297	12.117
Model 6	**Slope**	0.201	0.014	14.412	0.000	0.174	0.229
Model 7	**Intercept**	-129.989	17.061	-7.619	0.000	-163.564	-96.414
Model 7	**Slope**	3.909	0.473	8.262	0.000	2.978	4.840
Model 8	**Intercept**	9.420	2.355	4.001	0.000	4.787	14.054

medRxiv preprint doi: https://doi.org/10.64898/2026.04.07.26349269; this version posted April 8, 2026. The copyright holder for this preprint (which was not certified by peer review) is the author/funder, who has granted medRxiv a license to display the preprint in perpetuity.
It is made available under a CC-BY 4.0 International license .

Model 8	**Slope**	0.300	0.079	3.797	0.000	0.145	0.456

Table S8. Linear regression results for relative percent averted in Scenario E using the median horizon week of Scenario A as the independent variable.

**Model ID**	**Term**	**Estimate**	**Std. Error**	**t value**	**Pr(>|t|)**	**[0.025**	**0.975**
Model 1	**Intercept**	3.127	0.256	12.206	0.000	2.623	3.631
Model 1	**Slope**	-0.041	0.011	-3.865	0.000	-0.061	-0.020
Model 2	**Intercept**	1.680	0.110	15.243	0.000	1.463	1.896
Model 2	**Slope**	-0.017	0.004	-3.836	0.000	-0.026	-0.008
Model 3	**Intercept**	-27.054	0.563	-48.026	0.000	-28.163	-25.946
Model 3	**Slope**	0.741	0.014	51.199	0.000	0.713	0.770
Model 4	**Intercept**	0.944	0.037	25.376	0.000	0.870	1.017
Model 4	**Slope**	-0.040	0.001	-27.367	0.000	-0.043	-0.037
Model 5	**Intercept**	8.134	0.084	96.384	0.000	7.968	8.300
Model 5	**Slope**	-0.147	0.003	-43.686	0.000	-0.153	-0.140
Model 6	**Intercept**	2.333	0.468	4.981	0.000	1.411	3.255
Model 6	**Slope**	0.003	0.014	0.183	0.855	-0.025	0.030
Model 7	**Intercept**	-25.064	19.672	-1.274	0.204	-63.779	13.650
Model 7	**Slope**	0.616	0.546	1.129	0.26	-0.458	1.690
Model 8	**Intercept**	-0.351	0.465	-0.755	0.451	-1.266	0.564
Model 8	**Slope**	0.067	0.016	4.280	0.000	0.036	0.098

Table S9. Linear regression results for difference in relative percent averted in Scenario C vs. B, using the median horizon week of Scenario A as the independent variable.

**Model ID**	**Term**	**Estimate**	**Std. Error**	**t value**	**Pr(>|t|)**	**[0.025**	**0.975**
Model 1	**Intercept**	2.153	0.336	6.417	0.000	1.493	2.813

medRxiv preprint doi: https://doi.org/10.64898/2026.04.07.26349269; this version posted April 8, 2026. The copyright holder for this preprint (which was not certified by peer review) is the author/funder, who has granted medRxiv a license to display the preprint in perpetuity.
It is made available under a CC-BY 4.0 International license .

Model 1	**Slope**	0.007	0.014	0.522	0.602	-0.020	0.034
Model 2	**Intercept**	2.105	0.138	15.239	0.000	1.833	2.377
Model 2	**Slope**	-0.031	0.006	-5.592	0.000	-0.042	-0.020
Model 3	**Intercept**	-25.990	0.963	-26.977	0.000	-27.886	-24.094
Model 3	**Slope**	0.759	0.025	30.674	0.000	0.711	0.808
Model 4	**Intercept**	0.861	0.053	16.148	0.000	0.756	0.966
Model 4	**Slope**	-0.048	0.002	-22.705	0.000	-0.052	-0.044
Model 5	**Intercept**	8.456	0.146	57.856	0.000	8.168	8.743
Model 5	**Slope**	-0.103	0.006	-17.766	0.000	-0.115	-0.092
Model 6	**Intercept**	4.124	0.095	43.380	0.000	3.937	4.311
Model 6	**Slope**	-0.024	0.003	-8.431	0.000	-0.030	-0.019
Model 7	**Intercept**	-5.958	20.886	-0.285	0.776	-47.061	35.144
Model 7	**Slope**	-0.042	0.579	-0.072	0.943	-1.182	1.098
Model 8	**Intercept**	-2.485	0.906	-2.742	0.006	-4.269	-0.701
Model 8	**Slope**	0.140	0.030	4.607	0.000	0.080	0.200

Table S10. Linear regression results for difference in relative percent averted in Scenario E vs. D, using the median horizon week of Scenario A as the independent variable.

Ensemble model:

Dependent variable: Total epidemic size per 100k

- **Observations:** 4,13,740 | **Method:** REML | **Groups:** 52 | **Scale:** 3431.5937
- **Log-Likelihood:** -22,72,686.8089 | **Converged:** Yes

Variable	Coef.	Std.Err.	z	P>|z|	[0.025	0.975]
**Intercept**	211.601	5.113	41.388	0.000	201.581	221.622
**scenario_id[T.B-2025-04-01]**	-30.091	0.288	-104.483	0.000	-30.655	-29.526
**scenario_id[T.C-2025-04-01]**	-33.750	0.288	-117.189	0.000	-34.314	-33.185

medRxiv preprint doi: https://doi.org/10.64898/2026.04.07.26349269; this version posted April 8, 2026. The copyright holder for this preprint (which was not certified by peer review) is the author/funder, who has granted medRxiv a license to display the preprint in perpetuity.
It is made available under a CC-BY 4.0 International license .

**scenario_id[T.D-2025-04-01]**	-38.244	0.288	-132.793	0.000	-38.808	-37.679
**scenario_id[T.E-2025-04-01]**	-43.045	0.288	-149.465	0.000	-43.610	-42.481
**Group Var**	585.783	6.695				
**Team Var**	6135.740	7.629				

Dependent variable: Summer wave size per 100k

- **Observations:** 4,13,740 | **Method:** REML | **Groups:** 52 | **Scale:** 1305.6893
- **Log-Likelihood:** -20,72,854.6960 | **Converged:** Yes

Variable	Coef.	Std.Err.	z	P>|z|	[0.025	0.975]
**Intercept**	89.363	3.012	29.664	0.000	83.458	95.267
**scenario_id[T.B-2025-04-01]**	-3.730	0.178	-20.998	0.000	-4.078	-3.382
**scenario_id[T.C-2025-04-01]**	-9.029	0.178	-50.824	0.000	-9.377	-8.680
**scenario_id[T.D-2025-04-01]**	-4.365	0.178	-24.572	0.000	-4.713	-4.017
**scenario_id[T.E-2025-04-01]**	-11.212	0.178	-63.113	0.000	-11.560	-10.864
**Group Var**	51.146					
**Team Var**	3343.503	6.459				

Dependent variable: Winter wave size per 100k

- **Observations:** 4,13,740 | **Method:** REML | **Groups:** 52 | **Scale:** 1957.0916
- **Log-Likelihood:** -2156330.5556 | **Converged:** Yes

Variable	Coef.	Std.Err.	z	P>|z|	[0.025	0.975]
**Intercept**	122.222	2.941	41.555	0.000	116.458	127.987
**scenario_id[T.B-2025-04-01]**	-26.360	0.217	-121.202	0.000	-26.787	-25.934
**scenario_id[T.C-2025-04-01]**	-24.721	0.217	-113.665	0.000	-25.147	-24.295
**scenario_id[T.D-2025-04-01]**	-33.879	0.217	-155.770	0.000	-34.305	-33.452
**scenario_id[T.E-2025-04-01]**	-31.833	0.217	-146.366	0.000	-32.260	-31.407

medRxiv preprint doi: https://doi.org/10.64898/2026.04.07.26349269; this version posted April 8, 2026. The copyright holder for this preprint (which was not certified by peer review) is the author/funder, who has granted medRxiv a license to display the preprint in perpetuity.
It is made available under a CC-BY 4.0 International license .

**Group Var**	279.346	1.765				
**Team Var**	1345.835	2.287				

Table S11. Mixed effects analysis results for the ensemble model over the projection period (April 27, 2025 to April 25, 2026) using Scenario A as baseline, rest of the scenarios (B-E) as fixed effects and location (states) and teams (models) as random effects. The three dependent variables considered are: total, summer, and winter wave sizes per 100k population. Absolute values of the entries in the Coef. column for the scenario variables corresponds to the average number of hospitalizations averted per 100k population compared to Scenario A.

Scenario	Difference in effect on total epidemic size (mean±std)	Difference in effect on summer wave size (mean±std)	Difference in effect on winter wave size (mean±std)
B	12.49±10.71	1.35±1.07	11.91±9.47
C	12.87±11.27	2.58±1.83	12.74±9.41
D	15.64±13.99	1.69±1.53	14.70±12.50
E	16.46±15.67	3.72±2.79	15.78±12.95

Table S12. Mean difference across teams in scenario effects on the total, summer, and winter wave sizes between winter and summer leaning states over the projection period (April 27, 2025 to April 25, 2026). The difference is the number of excess hospitalizations averted per 100k individuals in the winter leaning states compared to the summer ones.

Scenario C - Scenario B (mean±std)		Scenario D - Scenario B (mean±std)	
**Summer leaning**	**Winter leaning**	**Summer leaning**	**Winter leaning**
7.57±4.67	5.80±4.94	0.78±0.43	0.87±0.83

Table S13. Mean difference across teams in the scenario effects on the summer wave size between scenarios C and B (left) and D and B (right) for both summer and winter leaning states over the projection period (April 27, 2025 to April 25, 2026). The difference is the number of excess hospitalizations averted per 100k individuals.

Scenario C - Scenario B (mean±std)		Scenario D - Scenario B (mean±std)	
**Summer leaning**	**Winter leaning**	**Summer leaning**	**Winter leaning**
-1.99±2.14	-1.31±2.14	5.30±2.77	8.89±6.27

medRxiv preprint doi: https://doi.org/10.64898/2026.04.07.26349269; this version posted April 8, 2026. The copyright holder for this preprint (which was not certified by peer review) is the author/funder, who has granted medRxiv a license to display the preprint in perpetuity.
It is made available under a CC-BY 4.0 International license .

Table S14. Mean difference across teams in the scenario effects on the winter wave size between scenarios C and B (left) and D and B (right) for both summer and winter leaning locations over the projection period (April 27, 2025 to April 25, 2026). The difference is the number of excess hospitalizations averted per 100k individuals.

Model 1:
Summer leaning locations

Dependent variable: Total epidemic size per 100k

- **Observations:** 64,500 | **Method:** REML | **Groups:** 43 | **Scale:** 99.8540
- **Log-Likelihood:** -240213.8892 | **Converged:** Yes

Variable	Coef.	Std.Err.	z	P>|z|	[0.025	0.975]
**Intercept**	250.804	7.000	35.829	0.000	237.084	264.523
**scenario_id[T.B-2025-04-01]**	-26.677	0.124	-214.404	0.000	-26.921	-26.433
**scenario_id[T.C-2025-04-01]**	-32.451	0.124	-260.811	0.000	-32.695	-32.207
**scenario_id[T.D-2025-04-01]**	-32.705	0.124	-262.850	0.000	-32.949	-32.461
**scenario_id[T.E-2025-04-01]**	-38.808	0.124	-311.904	0.000	-39.052	-38.564
**Group Var**	2106.728	46.023				

Dependent variable: Summer wave size per 100k

- **Observations:** 64,500 | **Method:** REML | **Groups:** 43 | **Scale:** 53.2433
- **Log-Likelihood:** -219925.3182 | **Converged:** Yes

Variable	Coef.	Std.Err.	z	P>|z|	[0.025	0.975]
**Intercept**	137.707	4.024	34.223	0.000	129.821	145.594
**scenario_id[T.B-2025-04-01]**	-5.594	0.091	-61.574	0.000	-5.772	-5.416
**scenario_id[T.C-2025-04-01]**	-15.075	0.091	-165.921	0.000	-15.253	-14.897
**scenario_id[T.D-2025-04-01]**	-6.827	0.091	-75.143	0.000	-7.005	-6.649
**scenario_id[T.E-2025-04-01]**	-18.954	0.091	-208.615	0.000	-19.132	-18.776
**Group Var**	696.024	20.823				

medRxiv preprint doi: https://doi.org/10.64898/2026.04.07.26349269; this version posted April 8, 2026. The copyright holder for this preprint (which was not certified by peer review) is the author/funder, who has granted medRxiv a license to display the preprint in perpetuity.
It is made available under a CC-BY 4.0 International license .

Dependent variable: Winter wave size per 100k

- **Observations:** 64,500 | **Method:** REML | **Groups:** 43 | **Scale:** 87.6574
- **Log-Likelihood:** -235983.4154 | **Converged:** Yes

Variable	Coef.	Std.Err.	z	P>|z|	[0.025	0.975]
**Intercept**	113.096	3.254	34.754	0.000	106.718	119.475
**scenario_id[T.B-2025-04-01]**	-21.083	0.117	-180.846	0.000	-21.311	-20.854
**scenario_id[T.C-2025-04-01]**	-17.376	0.117	-149.052	0.000	-17.605	-17.148
**scenario_id[T.D-2025-04-01]**	-25.877	0.117	-221.977	0.000	-26.106	-25.649
**scenario_id[T.E-2025-04-01]**	-19.854	0.117	-170.310	0.000	-20.083	-19.626
**Group Var**	455.059	10.611				

Winter leaning locations

Dependent variable: Total wave size per 100k

- **Observations:** 12,000 | **Method:** REML | **Groups:** 8 | **Scale:** 233.7945
- **Log-Likelihood:** -49785.4152 | **Converged:** Yes

Variable	Coef.	Std.Err.	z	P>|z|	[0.025	0.975]
**Intercept**	247.722	15.066	16.442	0.000	218.192	277.252
**scenario_id[T.B-2025-04-01]**	-45.081	0.441	-102.134	0.000	-45.947	-44.216
**scenario_id[T.C-2025-04-01]**	-52.758	0.441	-119.525	0.000	-53.623	-51.893
**scenario_id[T.D-2025-04-01]**	-57.144	0.441	-129.464	0.000	-58.010	-56.279
**scenario_id[T.E-2025-04-01]**	-65.176	0.441	-147.660	0.000	-66.041	-64.311
**Group Var**	1815.209	63.481				

Dependent variable: Summer wave size per 100k

- **Observations:** 12,000 | **Method:** REML | **Groups:** 8 | **Scale:** 88.4117
- **Log-Likelihood:** -43951.3050 | **Converged:** Yes

medRxiv preprint doi: https://doi.org/10.64898/2026.04.07.26349269; this version posted April 8, 2026. The copyright holder for this preprint (which was not certified by peer review) is the author/funder, who has granted medRxiv a license to display the preprint in perpetuity.
It is made available under a CC-BY 4.0 International license .

Variable	Coef.	Std.Err.	z	P>|z|	[0.025	0.975]
**Intercept**	114.825	7.050	16.288	0.000	101.007	128.642
**scenario_id[T.B-2025-04-01]**	-8.925	0.271	-32.882	0.000	-9.457	-8.393
**scenario_id[T.C-2025-04-01]**	-20.893	0.271	-76.973	0.000	-21.425	-20.361
**scenario_id[T.D-2025-04-01]**	-11.356	0.271	-41.838	0.000	-11.888	-10.824
**scenario_id[T.E-2025-04-01]**	-28.100	0.271	-103.524	0.000	-28.632	-27.568
**Group Var**	397.299	22.595				

Dependent variable: Winter wave size per 100k

- **Observations:** 12,000 | **Method:** REML | **Groups:** 8 | **Scale:** 212.2843
- **Log-Likelihood:** -49203.1133 | **Converged:** Yes

Variable	Coef.	Std.Err.	z	P>|z|	[0.025	0.975]
**Intercept**	132.897	8.778	15.139	0.000	115.692	150.103
**scenario_id[T.B-2025-04-01]**	-36.156	0.421	-85.963	0.000	-36.980	-35.332
**scenario_id[T.C-2025-04-01]**	-31.865	0.421	-75.760	0.000	-32.689	-31.040
**scenario_id[T.D-2025-04-01]**	-45.788	0.421	-108.864	0.000	-46.612	-44.964
**scenario_id[T.E-2025-04-01]**	-37.076	0.421	-88.151	0.000	-37.901	-36.252
**Group Var**	615.785	22.556				

Table S15. Mixed effects analysis results for Model 1 over the projection period (April 27, 2025 to April 25, 2026) using Scenario A as baseline, rest of the scenarios (B-E) as fixed effects and location (states) as random effects for both summer and winter leaning locations. The three dependent variables considered are: total, summer, and winter wave sizes per 100k population. Absolute values of the entries in the Coef. column for the scenario variables corresponds to the average number of hospitalizations averted per 100k population compared to Scenario A.

Model 2:
Summer leaning locations

Dependent variable: Total epidemic size per 100k

- **Observations:** 61,295 | **Method:** REML | **Groups:** 41 | **Scale:** 8314.1600

medRxiv preprint doi: https://doi.org/10.64898/2026.04.07.26349269; this version posted April 8, 2026. The copyright holder for this preprint (which was not certified by peer review) is the author/funder, who has granted medRxiv a license to display the preprint in perpetuity.
It is made available under a CC-BY 4.0 International license .

- **Log-Likelihood:** -363726.0163 | **Converged:** Yes

Variable	Coef.	Std.Err.	z	P>|z|	[0.025	0.975]
**Intercept**	209.845	13.066	16.061	0.000	184.236	235.454
**scenario_id[T.B-2025-04-01]**	-26.486	1.165	-22.742	0.000	-28.769	-24.203
**scenario_id[T.C-2025-04-01]**	-29.856	1.165	-25.635	0.000	-32.139	-27.573
**scenario_id[T.D-2025-04-01]**	-31.143	1.165	-26.740	0.000	-33.425	-28.860
**scenario_id[T.E-2025-04-01]**	-34.917	1.165	-29.981	0.000	-37.200	-32.635
**Group Var**	6971.596	17.108				

Dependent variable: Summer wave size per 100k

- **Observations:** 61,295 | **Method:** REML | **Groups:** 41 | **Scale:** 3696.5846
- **Log-Likelihood:** -338882.3445 | **Converged:** Yes

Variable	Coef.	Std.Err.	z	P>|z|	[0.025	0.975]
**Intercept**	116.727	7.819	14.929	0.000	101.402	132.051
**scenario_id[T.B-2025-04-01]**	-2.685	0.777	-3.458	0.001	-4.207	-1.163
**scenario_id[T.C-2025-04-01]**	-6.745	0.777	-8.685	0.000	-8.267	-5.223
**scenario_id[T.D-2025-04-01]**	-3.031	0.777	-3.903	0.000	-4.553	-1.509
**scenario_id[T.E-2025-04-01]**	-8.033	0.777	-10.344	0.000	-9.555	-6.511
**Group Var**	2494.086	9.181				

Dependent variable: Winter wave size per 100k

- **Observations:** 61,295 | **Method:** REML | **Groups:** 41 | **Scale:** 3246.9621
- **Log-Likelihood:** -334897.1394| **Converged:** Yes

Variable	Coef.	Std.Err.	z	P>|z|	[0.025	0.975]
**Intercept**	93.118	5.592	16.652	0.000	82.158	104.078
**scenario_id[T.B-2025-04-01]**	-23.801	0.728	-32.701	0.000	-25.227	-22.374

medRxiv preprint doi: https://doi.org/10.64898/2026.04.07.26349269; this version posted April 8, 2026. The copyright holder for this preprint (which was not certified by peer review) is the author/funder, who has granted medRxiv a license to display the preprint in perpetuity.
It is made available under a CC-BY 4.0 International license .

**scenario_id[T.C-2025-04-01]**	-23.111	0.728	-31.754	0.000	-24.538	-21.685
**scenario_id[T.D-2025-04-01]**	-28.112	0.728	-38.625	0.000	-29.539	-26.686
**scenario_id[T.E-2025-04-01]**	-26.884	0.728	-36.938	0.000	-28.311	-25.458
**Group Var**	1271.229	5.028				

Winter leaning locations

Dependent variable: Total epidemic size per 100k

- **Observations:** 16,445 | **Method:** REML | **Groups:** 11 | **Scale:** 21898.7069
- **Log-Likelihood:** -105526.2011 | **Converged:** Yes

Variable	Coef.	Std.Err.	z	P>|z|	[0.025	0.975]
**Intercept**	286.528	16.371	17.502	0.000	254.442	318.615
**scenario_id[T.B-2025-04-01]**	-41.825	3.649	-11.461	0.000	-48.977	-34.672
**scenario_id[T.C-2025-04-01]**	-45.933	3.649	-12.587	0.000	-53.086	-38.781
**scenario_id[T.D-2025-04-01]**	-47.315	3.649	-12.966	0.000	-54.467	-40.163
**scenario_id[T.E-2025-04-01]**	-51.584	3.649	-14.136	0.000	-58.736	-44.432
**Group Var**	2874.812	9.099				

Dependent variable: Summer wave size per 100k

- **Observations:** 16,445 | **Method:** REML | **Groups:** 11 | **Scale:** 3765.4061
- **Log-Likelihood:** -91056.5038 | **Converged:** Yes

Variable	Coef.	Std.Err.	z	P>|z|	[0.025	0.975]
**Intercept**	129.854	8.432	15.400	0.000	113.328	146.380
**scenario_id[T.B-2025-04-01]**	-3.428	1.513	-2.266	0.023	-6.394	-0.463
**scenario_id[T.C-2025-04-01]**	-8.264	1.513	-5.461	0.000	-11.230	-5.298
**scenario_id[T.D-2025-04-01]**	-3.824	1.513	-2.527	0.012	-6.790	-0.858

medRxiv preprint doi: https://doi.org/10.64898/2026.04.07.26349269; this version posted April 8, 2026. The copyright holder for this preprint (which was not certified by peer review) is the author/funder, who has granted medRxiv a license to display the preprint in perpetuity.
It is made available under a CC-BY 4.0 International license .

**scenario_id[T.E-2025-04-01]**	-9.632	1.513	-6.365	0.000	-12.598	-6.666
**Group Var**	769.458	5.570				

Dependent variable: Winter wave size per 100k

- **Observations:** 16,445 | **Method:** REML | **Groups:** 11 | **Scale:** 14979.7536
- **Log-Likelihood:** -102399.7489 | **Converged:** Yes

Variable	Coef.	Std.Err.	z	P>|z|	[0.025	0.975]
**Intercept**	156.675	8.282	18.917	0.000	140.442	172.908
**scenario_id[T.B-2025-04-01]**	-38.396	3.018	-12.722	0.000	-44.312	-32.481
**scenario_id[T.C-2025-04-01]**	-37.670	3.018	-12.481	0.000	-43.585	-31.754
**scenario_id[T.D-2025-04-01]**	-43.491	3.018	-14.410	0.000	-49.406	-37.575
**scenario_id[T.E-2025-04-01]**	-41.952	3.018	-13.900	0.000	-47.867	-36.037
**Group Var**	704.476	2.611				

Table S16. Mixed effects analysis results for Model 2 over the projection period (April 27, 2025 to April 25, 2026) using Scenario A as baseline, rest of the scenarios (B-E) as fixed effects and location (states) as random effects for both summer and winter leaning locations. The three dependent variables considered are: total, summer, and winter wave sizes per 100k population. Absolute values of the entries in the Coef. column for the scenario variables corresponds to the average number of hospitalizations averted per 100k population compared to Scenario A.

Model 3:
Winter leaning locations

Dependent variable: Total epidemic size per 100k

- **Observations:** 78,000 | **Method:** REML | **Groups:** 52 | **Scale:** 3769.3318
- **Log-Likelihood:** -431993.3530 | **Converged:** Yes

Variable	Coef.	Std.Err.	z	P>|z|	[0.025	0.975]
**Intercept**	223.509	5.967	37.455	0.000	211.813	235.204
**scenario_id[T.B-2025-04-01]**	-53.063	0.695	-76.332	0.000	-54.425	-51.700
**scenario_id[T.C-2025-04-01]**	-55.654	0.695	-80.060	0.000	-57.017	-54.292

medRxiv preprint doi: https://doi.org/10.64898/2026.04.07.26349269; this version posted April 8, 2026. The copyright holder for this preprint (which was not certified by peer review) is the author/funder, who has granted medRxiv a license to display the preprint in perpetuity.
It is made available under a CC-BY 4.0 International license .

Variable	Coef.	Std.Err.	z	P>|z|	[0.025	0.975]
**scenario_id[T.D-2025-04-01]**	-71.003	0.695	-102.140	0.000	-72.366	-69.641
**scenario_id[T.E-2025-04-01]**	-77.439	0.695	-111.397	0.000	-78.801	-76.076
**Group Var**	1839.142	5.921				

Dependent variable: Summer wave size per 100k

- **Observations:** 78,000 | **Method:** REML | **Groups:** 52 | **Scale:** 670.3479
- **Log-Likelihood:** -364643.3348 | **Converged:** Yes

Variable	Coef.	Std.Err.	z	P>|z|	[0.025	0.975]
**Intercept**	28.889	2.198	13.143	0.000	24.581	33.197
**scenario_id[T.B-2025-04-01]**	-0.696	0.293	-2.374	0.018	-1.271	-0.121
**scenario_id[T.C-2025-04-01]**	-3.233	0.293	-11.027	0.000	-3.807	-2.658
**scenario_id[T.D-2025-04-01]**	-0.831	0.293	-2.833	0.005	-1.405	-0.256
**scenario_id[T.E-2025-04-01]**	-3.970	0.293	-13.541	0.000	-4.544	-3.395
**Group Var**	248.999	1.902				

Dependent variable: Winter wave size per 100k

- **Observations:** 78,000 | **Method:** REML | **Groups:** 52 | **Scale:** 2423.9763
- **Log-Likelihood:** -414773.6005 | **Converged:** Yes

Variable	Coef.	Std.Err.	z	P>|z|	[0.025	0.975]
**Intercept**	194.620	4.531	42.957	0.000	185.740	203.500
**scenario_id[T.B-2025-04-01]**	-52.367	0.557	-93.937	0.000	-53.459	-51.274
**scenario_id[T.C-2025-04-01]**	-52.422	0.557	-94.036	0.000	-53.514	-51.329
**scenario_id[T.D-2025-04-01]**	-70.173	0.557	-125.879	0.000	-71.265	-69.080
**scenario_id[T.E-2025-04-01]**	-73.469	0.557	-131.792	0.000	-74.562	-72.376
**Group Var**	1059.298	4.270				

medRxiv preprint doi: https://doi.org/10.64898/2026.04.07.26349269; this version posted April 8, 2026. The copyright holder for this preprint (which was not certified by peer review) is the author/funder, who has granted medRxiv a license to display the preprint in perpetuity.
It is made available under a CC-BY 4.0 International license .

Table S17. Mixed effects analysis results for Model 3 over the projection period (April 27, 2025 to April 25, 2026) using Scenario A as baseline, rest of the scenarios (B-E) as fixed effects and location (states) as random effects for winter leaning locations (model has no summer leaning locations). The three dependent variables considered are: total, summer, and winter wave sizes per 100k population. Absolute values of the entries in the Coef. column for the scenario variables corresponds to the average number of hospitalizations averted per 100k population compared to Scenario A.

Model 4:
Summer leaning locations

Dependent variable: Total epidemic size per 100k

- **Observations:** 36,000 | **Method:** REML | **Groups:** 24 | **Scale:** 2123.2791
- **Log-Likelihood:** -189059.4547 | **Converged:** Yes

Variable	Coef.	Std.Err.	z	P>|z|	[0.025	0.975]
**Intercept**	219.031	11.550	18.964	0.000	196.393	241.668
**scenario_id[T.B-2025-04-01]**	-10.147	0.768	-13.212	0.000	-11.652	-8.642
**scenario_id[T.C-2025-04-01]**	-10.453	0.768	-13.611	0.000	-11.958	-8.948
**scenario_id[T.D-2025-04-01]**	-12.334	0.768	-16.060	0.000	-13.839	-10.828
**scenario_id[T.E-2025-04-01]**	-12.169	0.768	-15.845	0.000	-13.674	-10.663
**Group Var**	3194.561	20.425				

Dependent variable: Summer wave size per 100k

- **Observations:** 36,000 | **Method:** REML | **Groups:** 24 | **Scale:** 2128.1653
- **Log-Likelihood:** -189089.7923 | **Converged:** Yes

Variable	Coef.	Std.Err.	z	P>|z|	[0.025	0.975]
**Intercept**	125.935	7.168	17.569	0.000	111.886	139.984
**scenario_id[T.B-2025-04-01]**	-5.293	0.769	-6.884	0.000	-6.800	-3.786
**scenario_id[T.C-2025-04-01]**	-8.926	0.769	-11.610	0.000	-10.433	-7.419
**scenario_id[T.D-2025-04-01]**	-5.749	0.769	-7.477	0.000	-7.256	-4.242
**scenario_id[T.E-2025-04-01]**	-10.499	0.769	-13.655	0.000	-12.006	-8.992

medRxiv preprint doi: https://doi.org/10.64898/2026.04.07.26349269; this version posted April 8, 2026. The copyright holder for this preprint (which was not certified by peer review) is the author/funder, who has granted medRxiv a license to display the preprint in perpetuity.
It is made available under a CC-BY 4.0 International license .

**Group Var**	1225.995	7.810				

Dependent variable: Winter wave size per 100k

- **Observations:** 36,000 | **Method:** REML | **Groups:** 24 | **Scale:** 1020.4034
- **Log-Likelihood:** -175862.8704 | **Converged:** Yes

Variable	Coef.	Std.Err.	z	P>|z|	[0.025	0.975]
**Intercept**	93.096	5.497	16.934	0.000	82.321	103.871
**scenario_id[T.B-2025-04-01]**	-4.854	0.532	-9.116	0.000	-5.897	-3.810
**scenario_id[T.C-2025-04-01]**	-1.527	0.532	-2.868	0.004	-2.570	-0.483
**scenario_id[T.D-2025-04-01]**	-6.585	0.532	-12.368	0.000	-7.628	-5.541
**scenario_id[T.E-2025-04-01]**	-1.670	0.532	-3.136	0.002	-2.713	-0.626
**Group Var**	721.917	6.683				

Winter leaning locations

Dependent variable: Total epidemic size per 100k

- **Observations:** 42,000 | **Method:** REML | **Groups:** 28 | **Scale:** 1591.9191
- **Log-Likelihood:** -214520.0154 | **Converged:** Yes

Variable	Coef.	Std.Err.	z	P>|z|	[0.025	0.975]
**Intercept**	232.046	8.189	28.336	0.000	215.996	248.097
**scenario_id[T.B-2025-04-01]**	-10.744	0.616	-17.452	0.000	-11.951	-9.538
**scenario_id[T.C-2025-04-01]**	-11.062	0.616	-17.967	0.000	-12.268	-9.855
**scenario_id[T.D-2025-04-01]**	-12.044	0.616	-19.563	0.000	-13.250	-10.837
**scenario_id[T.E-2025-04-01]**	-12.075	0.616	-19.613	0.000	-13.282	-10.868
**Group Var**	1872.462	12.784				

Dependent variable: Summer wave size per 100k

medRxiv preprint doi: https://doi.org/10.64898/2026.04.07.26349269; this version posted April 8, 2026. The copyright holder for this preprint (which was not certified by peer review) is the author/funder, who has granted medRxiv a license to display the preprint in perpetuity.
It is made available under a CC-BY 4.0 International license .

- **Observations:** 42,000 | **Method:** REML | **Groups:** 28 | **Scale:** 1447.9722
- **Log-Likelihood:** -212511.6866 | **Converged:** Yes

Variable	Coef.	Std.Err.	z	P>|z|	[0.025	0.975]
**Intercept**	105.685	3.983	26.532	0.000	97.878	113.492
**scenario_id[T.B-2025-04-01]**	-4.858	0.587	-8.274	0.000	-6.009	-3.707
**scenario_id[T.C-2025-04-01]**	-7.608	0.587	-12.957	0.000	-8.759	-6.457
**scenario_id[T.D-2025-04-01]**	-5.109	0.587	-8.701	0.000	-6.260	-3.958
**scenario_id[T.E-2025-04-01]**	-8.468	0.587	-14.422	0.000	-9.619	-7.317
**Group Var**	439.452	3.160				

Dependent variable: Winter wave size per 100k

- **Observations:** 42,000 | **Method:** REML | **Groups:** 28 | **Scale:** 1435.8051
- **Log-Likelihood:** -212338.9519 | **Converged:** Yes

Variable	Coef.	Std.Err.	z	P>|z|	[0.025	0.975]
**Intercept**	126.361	4.672	27.049	0.000	117.205	135.517
**scenario_id[T.B-2025-04-01]**	-5.886	0.585	-10.067	0.000	-7.032	-4.740
**scenario_id[T.C-2025-04-01]**	-3.454	0.585	-5.907	0.000	-4.600	-2.308
**scenario_id[T.D-2025-04-01]**	-6.935	0.585	-11.861	0.000	-8.081	-5.789
**scenario_id[T.E-2025-04-01]**	-3.607	0.585	-6.168	0.000	-4.753	-2.461
**Group Var**	606.287	4.361				

Table S18. Mixed effects analysis results for Model 4 over the projection period (April 27, 2025 to April 25, 2026) using Scenario A as baseline, rest of the scenarios (B-E) as fixed effects and location (states) as random effects for both summer and winter leaning locations. The three dependent variables considered are: total, summer, and winter wave sizes per 100k population. Absolute values of the entries in the Coef. column for the scenario variables corresponds to the average number of hospitalizations averted per 100k population compared to Scenario A.

<u>Model 5:</u>
Summer leaning locations

medRxiv preprint doi: https://doi.org/10.64898/2026.04.07.26349269; this version posted April 8, 2026. The copyright holder for this preprint (which was not certified by peer review) is the author/funder, who has granted medRxiv a license to display the preprint in perpetuity.
It is made available under a CC-BY 4.0 International license .

Dependent variable: Total epidemic size per 100k

- **Observations:**13,500 | **Method:** REML | **Groups:** 27 | **Scale:** 1781.8972
- **Log-Likelihood:** -69760.2172 | **Converged:** Yes

Variable	Coef.	Std.Err.	z	P>|z|	[0.025	0.975]
**Intercept**	331.868	9.193	36.099	0.000	313.850	349.887
**scenario_id[T.B-2025-04-01]**	-39.175	1.149	-34.099	0.000	-41.427	-36.924
**scenario_id[T.C-2025-04-01]**	-54.127	1.149	-47.113	0.000	-56.379	-51.875
**scenario_id[T.D-2025-04-01]**	-49.900	1.149	-43.434	0.000	-52.152	-47.649
**scenario_id[T.E-2025-04-01]**	-68.917	1.149	-59.986	0.000	-71.169	-66.665
**Group Var**	2264.100	14.913				

Dependent variable: Summer wave size per 100k

- **Observations:** 13,500 | **Method:** REML | **Groups:** 27 | **Scale:** 1593.4632
- **Log-Likelihood:** -68999.5256 | **Converged:** Yes

Variable	Coef.	Std.Err.	z	P>|z|	[0.025	0.975]
**Intercept**	198.181	6.776	29.247	0.000	184.900	211.462
**scenario_id[T.B-2025-04-01]**	-7.045	1.086	-6.485	0.000	-9.175	-4.916
**scenario_id[T.C-2025-04-01]**	-22.602	1.086	-20.804	0.000	-24.732	-20.473
**scenario_id[T.D-2025-04-01]**	-8.014	1.086	-7.376	0.000	-10.143	-5.884
**scenario_id[T.E-2025-04-01]**	-27.131	1.086	-24.972	0.000	-29.260	-25.001
**Group Var**	1223.824	8.532				

Dependent variable: Winter wave size per 100k

- **Observations:** 13,500 | **Method:** REML | **Groups:** 27 | **Scale:** 663.6187
- **Log-Likelihood:**-63090.2404 | **Converged:** Yes

Variable	Coef.	Std.Err.	z	P>|z|	[0.025	0.975]
**Intercept**	133.687	4.584	29.165	0.000	124.703	142.671

medRxiv preprint doi: https://doi.org/10.64898/2026.04.07.26349269; this version posted April 8, 2026. The copyright holder for this preprint (which was not certified by peer review) is the author/funder, who has granted medRxiv a license to display the preprint in perpetuity.
It is made available under a CC-BY 4.0 International license .

**scenario_id[T.B-2025-04-01]**	-32.130	0.701	-45.827	0.000	-33.504	-30.756
**scenario_id[T.C-2025-04-01]**	-31.525	0.701	-44.963	0.000	-32.899	-30.150
**scenario_id[T.D-2025-04-01]**	-41.887	0.701	-59.743	0.000	-43.261	-40.513
**scenario_id[T.E-2025-04-01]**	-41.786	0.701	-59.600	0.000	-43.161	-40.412
**Group Var**	560.658	6.054				

Winter leaning locations

Dependent variable: Total epidemic size per 100k

- **Observations:** 12,500 | **Method:** REML | **Groups:** 25 | **Scale:** 1900.1115
- **Log-Likelihood:** -64986.1611 | **Converged:** Yes

Variable	Coef.	Std.Err.	z	P>|z|	[0.025	0.975]
**Intercept**	361.245	7.372	49.004	0.000	346.796	375.693
**scenario_id[T.B-2025-04-01]**	-68.746	1.233	-55.759	0.000	-71.163	-66.330
**scenario_id[T.C-2025-04-01]**	-84.234	1.233	-68.321	0.000	-86.650	-81.817
**scenario_id[T.D-2025-04-01]**	-88.355	1.233	-71.663	0.000	-90.772	-85.939
**scenario_id[T.E-2025-04-01]**	-111.125	1.233	-90.132	0.000	-113.542	-108.709
**Group Var**	1339.540	8.917				

Dependent variable: Summer wave size per 100k

- **Observations:** 12,500 | **Method:** REML | **Groups:** 25 | **Scale:** 1502.0085
- **Log-Likelihood:** -63518.1650 | **Converged:** Yes

Variable	Coef.	Std.Err.	z	P>|z|	[0.025	0.975]
**Intercept**	145.168	6.788	21.387	0.000	131.864	158.472
**scenario_id[T.B-2025-04-01]**	-8.774	1.096	-8.004	0.000	-10.922	-6.625
**scenario_id[T.C-2025-04-01]**	-23.392	1.096	-21.340	0.000	-25.541	-21.244

medRxiv preprint doi: https://doi.org/10.64898/2026.04.07.26349269; this version posted April 8, 2026. The copyright holder for this preprint (which was not certified by peer review) is the author/funder, who has granted medRxiv a license to display the preprint in perpetuity.
It is made available under a CC-BY 4.0 International license .

**scenario_id[T.D-2025-04-01]**	-10.294	1.096	-9.391	0.000	-12.442	-8.145
**scenario_id[T.E-2025-04-01]**	-29.518	1.096	-26.928	0.000	-31.667	-27.370
**Group Var**	1136.785	8.497				

Dependent variable: Winter wave size per 100k

- **Observations:** 12,500 | **Method:** REML | **Groups:** 25 | **Scale:** 922.6426
- **Log-Likelihood:** -60461.0528 | **Converged:** Yes

Variable	Coef.	Std.Err.	z	P>|z|	[0.025	0.975]
**Intercept**	216.077	3.177	68.012	0.000	209.850	222.304
**scenario_id[T.B-2025-04-01]**	-59.972	0.859	-69.805	0.000	-61.656	-58.288
**scenario_id[T.C-2025-04-01]**	-60.841	0.859	-70.817	0.000	-62.525	-59.157
**scenario_id[T.D-2025-04-01]**	-78.061	0.859	-90.860	0.000	-79.745	-76.378
**scenario_id[T.E-2025-04-01]**	-81.607	0.859	-94.987	0.000	-83.291	-79.923
**Group Var**	243.115	2.330				

Table S19. Mixed effects analysis results for Model 5 over the projection period (April 27, 2025 to April 25, 2026) using Scenario A as baseline, rest of the scenarios (B-E) as fixed effects and location (states) as random effects for both summer and winter leaning locations. The three dependent variables considered are: total, summer, and winter wave sizes per 100k population. Absolute values of the entries in the Coef. column for the scenario variables corresponds to the average number of hospitalizations averted per 100k population compared to Scenario A.

Model 6:

Summer leaning locations

Dependent variable: Total epidemic size per 100k

- **Observations:** 3,500 | **Method:** REML | **Groups:** 7 | **Scale:** 472.7684
- **Log-Likelihood:** -15756.9472 | **Converged:** Yes

Variable	Coef.	Std.Err.	z	P>|z|	[0.025	0.975]
**Intercept**	130.365	9.070	14.373	0.000	112.587	148.142
**scenario_id[T.B-2025-04-01]**	-16.446	1.162	-14.150	0.000	-18.723	-14.168

medRxiv preprint doi: https://doi.org/10.64898/2026.04.07.26349269; this version posted April 8, 2026. The copyright holder for this preprint (which was not certified by peer review) is the author/funder, who has granted medRxiv a license to display the preprint in perpetuity.
It is made available under a CC-BY 4.0 International license .

**scenario_id[T.C-2025-04-01]**	-21.318	1.162	-18.342	0.000	-23.595	-19.040
**scenario_id[T.D-2025-04-01]**	-20.947	1.162	-18.023	0.000	-23.225	-18.669
**scenario_id[T.E-2025-04-01]**	-28.235	1.162	-24.293	0.000	-30.512	-25.957
**Group Var**	571.171	15.203				

Dependent variable: Summer wave size per 100k

- **Observations:** 3,500 | **Method:** REML | **Groups:** 7 | **Scale:** 139.7157
- **Log-Likelihood:** -13627.4697 | **Converged:** Yes

Variable	Coef.	Std.Err.	z	P>|z|	[0.025	0.975]
**Intercept**	72.004	5.554	12.964	0.000	61.119	82.890
**scenario_id[T.B-2025-04-01]**	-3.518	0.632	-5.568	0.000	-4.756	-2.280
**scenario_id[T.C-2025-04-01]**	-7.522	0.632	-11.905	0.000	-8.760	-6.283
**scenario_id[T.D-2025-04-01]**	-3.920	0.632	-6.205	0.000	-5.158	-2.682
**scenario_id[T.E-2025-04-01]**	-9.708	0.632	-15.366	0.000	-10.946	-8.470
**Group Var**	214.541	10.503				

Dependent variable: Winter wave size per 100k

- **Observations:** 3,500 | **Method:** REML | **Groups:** 7 | **Scale:** 106.5870
- **Log-Likelihood:** -13153.5826 | **Converged:** Yes

Variable	Coef.	Std.Err.	z	P>|z|	[0.025	0.975]
**Intercept**	58.360	4.158	14.035	0.000	50.210	66.510
**scenario_id[T.B-2025-04-01]**	-12.928	0.552	-23.426	0.000	-14.009	-11.846
**scenario_id[T.C-2025-04-01]**	-13.796	0.552	-25.000	0.000	-14.878	-12.714
**scenario_id[T.D-2025-04-01]**	-17.027	0.552	-30.855	0.000	-18.109	-15.945
**scenario_id[T.E-2025-04-01]**	-18.526	0.552	-33.572	0.000	-19.608	-17.445
**Group Var**	119.969	6.727				

medRxiv preprint doi: https://doi.org/10.64898/2026.04.07.26349269; this version posted April 8, 2026. The copyright holder for this preprint (which was not certified by peer review) is the author/funder, who has granted medRxiv a license to display the preprint in perpetuity.
It is made available under a CC-BY 4.0 International license .

Winter leaning locations

Dependent variable: Total epidemic size per 100k

- **Observations:** 22,500 | **Method:** REML | **Groups:** 45 | **Scale:** 438.3817
- **Log-Likelihood:**-100496.7127 | **Converged:** Yes

Variable	Coef.	Std.Err.	z	P>|z|	[0.025	0.975]
**Intercept**	127.324	3.161	40.280	0.000	121.129	133.520
**scenario_id[T.B-2025-04-01]**	-21.971	0.441	-49.776	0.000	-22.836	-21.106
**scenario_id[T.C-2025-04-01]**	-26.054	0.441	-59.025	0.000	-26.919	-25.189
**scenario_id[T.D-2025-04-01]**	-26.982	0.441	-61.127	0.000	-27.847	-26.117
**scenario_id[T.E-2025-04-01]**	-32.816	0.441	-74.345	0.000	-33.681	-31.951
**Group Var**	445.244	4.547				

Dependent variable: Summer wave size per 100k

- **Observations:** 22,500 | **Method:** REML | **Groups:** 45 | **Scale:** 59.4171
- **Log-Likelihood:** -78040.2037 | **Converged:** Yes

Variable	Coef.	Std.Err.	z	P>|z|	[0.025	0.975]
**Intercept**	43.280	1.901	22.764	0.000	39.554	47.006
**scenario_id[T.B-2025-04-01]**	-2.348	0.163	-14.448	0.000	-2.666	-2.029
**scenario_id[T.C-2025-04-01]**	-4.694	0.163	-28.885	0.000	-5.012	-4.375
**scenario_id[T.D-2025-04-01]**	-2.582	0.163	-15.890	0.000	-2.901	-2.264
**scenario_id[T.E-2025-04-01]**	-5.818	0.163	-35.804	0.000	-6.137	-5.500
**Group Var**	162.063	4.490				

Dependent variable: Winter wave size per 100k

- **Observations:** 22,500 | **Method:** REML | **Groups:** 45 | **Scale:** 190.3056
- **Log-Likelihood:** -91109.4597 | **Converged:** Yes

medRxiv preprint doi: https://doi.org/10.64898/2026.04.07.26349269; this version posted April 8, 2026. The copyright holder for this preprint (which was not certified by peer review) is the author/funder, who has granted medRxiv a license to display the preprint in perpetuity.
It is made available under a CC-BY 4.0 International license .

Variable	Coef.	Std.Err.	z	P>|z|	[0.025	0.975]
**Intercept**	84.044	2.005	41.908	0.000	80.114	87.975
**scenario_id[T.B-2025-04-01]**	-19.623	0.291	-67.474	0.000	-20.193	-19.053
**scenario_id[T.C-2025-04-01]**	-21.360	0.291	-73.445	0.000	-21.930	-20.790
**scenario_id[T.D-2025-04-01]**	-24.399	0.291	-83.897	0.000	-24.969	-23.829
**scenario_id[T.E-2025-04-01]**	-26.998	0.291	-92.831	0.000	-27.568	-26.428
**Group Var**	179.083	2.776				

Table S20. Mixed effects analysis results for Model 6 over the projection period (April 27, 2025 to April 25, 2026) using Scenario A as baseline, rest of the scenarios (B-E) as fixed effects and location (states) as random effects for both summer and winter leaning locations. The three dependent variables considered are: total, summer, and winter wave sizes per 100k population. Absolute values of the entries in the Coef. column for the scenario variables corresponds to the average number of hospitalizations averted per 100k population compared to Scenario A.

Model 7:
Winter leaning locations

Dependent variable: Total epidemic size per 100k

- **Observations:** 25,500 | **Method:** REML | **Groups:** 51 | **Scale:** 100.2266
- **Log-Likelihood:** -95151.3127 | **Converged:** Yes

Variable	Coef.	Std.Err.	z	P>|z|	[0.025	0.975]
**Intercept**	78.196	5.198	15.044	0.000	68.009	88.384
**scenario_id[T.B-2025-04-01]**	-9.668	0.198	-48.765	0.000	-10.056	-9.279
**scenario_id[T.C-2025-04-01]**	-7.234	0.198	-36.487	0.000	-7.622	-6.845
**scenario_id[T.D-2025-04-01]**	-15.055	0.198	-75.939	0.000	-15.444	-14.667
**scenario_id[T.E-2025-04-01]**	-10.146	0.198	-51.176	0.000	-10.534	-9.757
**Group Var**	1376.808	27.536				

Dependent variable: Summer wave size per 100k

medRxiv preprint doi: https://doi.org/10.64898/2026.04.07.26349269; this version posted April 8, 2026. The copyright holder for this preprint (which was not certified by peer review) is the author/funder, who has granted medRxiv a license to display the preprint in perpetuity.
It is made available under a CC-BY 4.0 International license .

- **Observations:** 25,500 | **Method:** REML | **Groups:** 51 | **Scale:** 8.8444
- **Log-Likelihood:**-64199.9922 | **Converged:** Yes

Variable	Coef.	Std.Err.	z	P>|z|	[0.025	0.975]
**Intercept**	13.996	1.402	9.987	0.000	11.249	16.743
**scenario_id[T.B-2025-04-01]**	-1.344	0.059	-22.813	0.000	-1.459	-1.228
**scenario_id[T.C-2025-04-01]**	-2.454	0.059	-41.661	0.000	-2.569	-2.338
**scenario_id[T.D-2025-04-01]**	-1.907	0.059	-32.377	0.000	-2.022	-1.791
**scenario_id[T.E-2025-04-01]**	-3.785	0.059	-64.271	0.000	-3.901	-3.670
**Group Var**	100.089	6.739				

Dependent variable: Winter wave size per 100k

- **Observations:** 25,500 | **Method:** REML | **Groups:** 51 | **Scale:** 116.2437
- **Log-Likelihood:** -97023.9331 | **Converged:** Yes

Variable	Coef.	Std.Err.	z	P>|z|	[0.025	0.975]
**Intercept**	64.200	3.965	16.193	0.000	56.429	71.971
**scenario_id[T.B-2025-04-01]**	-8.324	0.214	-38.988	0.000	-8.743	-7.906
**scenario_id[T.C-2025-04-01]**	-4.780	0.214	-22.388	0.000	-5.199	-4.362
**scenario_id[T.D-2025-04-01]**	-13.148	0.214	-61.583	0.000	-13.567	-12.730
**scenario_id[T.E-2025-04-01]**	-6.361	0.214	-29.792	0.000	-6.779	-5.942
**Group Var**	800.541	14.869				

Table S21. Mixed effects analysis results for Model 7 over the projection period (April 27, 2025 to April 25, 2026) using Scenario A as baseline, rest of the scenarios (B-E) as fixed effects and location (states) as random effects for winter leaning locations (model has no summer leaning locations). The three dependent variables considered are: total, summer, and winter wave sizes per 100k population. Absolute values of the entries in the Coef. column for the scenario variables corresponds to the average number of hospitalizations averted per 100k population compared to Scenario A.

Model 8:
Summer leaning locations

medRxiv preprint doi: https://doi.org/10.64898/2026.04.07.26349269; this version posted April 8, 2026. The copyright holder for this preprint (which was not certified by peer review) is the author/funder, who has granted medRxiv a license to display the preprint in perpetuity.
It is made available under a CC-BY 4.0 International license .

Dependent variable: Total epidemic size per 100k

- **Observations:** 6000 | **Method:** REML | **Groups:** 12 | **Scale:** 332.5050
- **Log-Likelihood:** -25974.1610 | **Converged:** Yes

Variable	Coef.	Std.Err.	z	P>|z|	[0.025	0.975]
**Intercept**	226.950	13.400	16.937	0.000	200.687	253.213
**scenario_id[T.B-2025-04-01]**	-24.022	0.744	-32.268	0.000	-25.481	-22.563
**scenario_id[T.C-2025-04-01]**	-28.218	0.744	-37.905	0.000	-29.677	-26.759
**scenario_id[T.D-2025-04-01]**	-32.370	0.744	-43.483	0.000	-33.829	-30.911
**scenario_id[T.E-2025-04-01]**	-36.827	0.744	-49.470	0.000	-38.286	-35.368
**Group Var**	2151.324	50.368				

Dependent variable: Summer wave size per 100k

- **Observations:** 6000 | **Method:** REML | **Groups:** 12 | **Scale:** 851.5527
- **Log-Likelihood:** -28784.3126 | **Converged:** Yes

Variable	Coef.	Std.Err.	z	P>|z|	[0.025	0.975]
**Intercept**	126.726	9.731	13.022	0.000	107.653	145.799
**scenario_id[T.B-2025-04-01]**	-4.450	1.191	-3.735	0.000	-6.785	-2.115
**scenario_id[T.C-2025-04-01]**	-13.148	1.191	-11.036	0.000	-15.483	-10.813
**scenario_id[T.D-2025-04-01]**	-5.715	1.191	-4.797	0.000	-8.050	-3.380
**scenario_id[T.E-2025-04-01]**	-17.452	1.191	-14.649	0.000	-19.787	-15.117
**Group Var**	1127.893	16.536				

Dependent variable: Winter wave size per 100k

- **Observations:** 6000 | **Method:** REML | **Groups:** 12 | **Scale:** 577.2962
- **Log-Likelihood:** -27615.3503 | **Converged:** Yes

Variable	Coef.	Std.Err.	z	P>|z|	[0.025	0.975]
**Intercept**	100.224	5.681	17.643	0.000	89.090	111.358

medRxiv preprint doi: https://doi.org/10.64898/2026.04.07.26349269; this version posted April 8, 2026. The copyright holder for this preprint (which was not certified by peer review) is the author/funder, who has granted medRxiv a license to display the preprint in perpetuity.
It is made available under a CC-BY 4.0 International license .

**scenario_id[T.B-2025-04-01]**	-19.572	0.981	-19.953	0.000	-21.494	-17.649
**scenario_id[T.C-2025-04-01]**	-15.070	0.981	-15.363	0.000	-16.992	-13.147
**scenario_id[T.D-2025-04-01]**	-26.655	0.981	-27.174	0.000	-28.577	-24.732
**scenario_id[T.E-2025-04-01]**	-19.375	0.981	-19.752	0.000	-21.298	-17.453
**Group Var**	381.459	6.790				

Winter leaning locations

Dependent variable: Total epidemic size per 100k

- **Observations:** 20,000 | **Method:** REML | **Groups:** 40 | **Scale:** 285.9056
- **Log-Likelihood:** -85097.5179 | **Converged:** Yes

Variable	Coef.	Std.Err.	z	P>|z|	[0.025	0.975]
**Intercept**	206.778	7.741	26.711	0.000	191.606	221.951
**scenario_id[T.B-2025-04-01]**	-29.530	0.378	-78.103	0.000	-30.271	-28.789
**scenario_id[T.C-2025-04-01]**	-33.595	0.378	-88.855	0.000	-34.336	-32.854
**scenario_id[T.D-2025-04-01]**	-40.300	0.378	-107.911	0.000	-41.541	-40.059
**scenario_id[T.E-2025-04-01]**	-45.697	0.378	-120.863	0.000	-46.438	-44.956
**Group Var**	2394.240	32.105				

Dependent variable: Summer wave size per 100k

- **Observations:** 20,000 | **Method:** REML | **Groups:** 40 | **Scale:** 349.2814
- **Log-Likelihood:** -87081.6382 | **Converged:** Yes

Variable	Coef.	Std.Err.	z	P>|z|	[0.025	0.975]
**Intercept**	72.482	5.461	13.273	0.000	61.779	83.185
**scenario_id[T.B-2025-04-01]**	-3.732	0.418	-8.931	0.000	-4.551	-2.913
**scenario_id[T.C-2025-04-01]**	-9.961	0.418	-23.835	0.000	-10.780	-9.142

medRxiv preprint doi: https://doi.org/10.64898/2026.04.07.26349269; this version posted April 8, 2026. The copyright holder for this preprint (which was not certified by peer review) is the author/funder, who has granted medRxiv a license to display the preprint in perpetuity.
It is made available under a CC-BY 4.0 International license .

**scenario_id[T.D-2025-04-01]**	-5.141	0.418	-12.301	0.000	-5.960	-4.322
**scenario_id[T.E-2025-04-01]**	-14.181	0.418	-33.934	0.000	-15.000	-13.362
**Group Var**	1189.335	14.434				

Dependent variable: Winter wave size per 100k

- **Observations:** 20,000 | **Method:** REML | **Groups:** 40 | **Scale:** 284.4008
- **Log-Likelihood:** -85024.2881 | **Converged:** Yes

Variable	Coef.	Std.Err.	z	P>|z|	[0.025	0.975]
**Intercept**	134.296	4.572	29.375	0.000	125.336	143.257
**scenario_id[T.B-2025-04-01]**	-25.798	0.377	-68.412	0.000	-26.537	-25.059
**scenario_id[T.C-2025-04-01]**	-23.635	0.377	-62.676	0.000	-24.374	-22.896
**scenario_id[T.D-2025-04-01]**	-35.659	0.377	-94.564	0.000	-36.399	-34.920
**scenario_id[T.E-2025-04-01]**	-31.516	0.377	-83.576	0.000	-32.255	-30.777
**Group Var**	833.202	11.207				

Table S22. Mixed effects analysis results for Model 8 over the projection period (April 27, 2025 to April 25, 2026) using Scenario A as baseline, rest of the scenarios (B-E) as fixed effects and location (states) as random effects for both summer and winter leaning locations. The three dependent variables considered are: total, summer, and winter wave sizes per 100k population. Absolute values of the entries in the Coef. column for the scenario variables corresponds to the average number of hospitalizations averted per 100k population compared to Scenario A.

5. Code availability

All analyses were performed in *Python*[7] (with additional libraries *NumPy, SciPy*, *statsmodels*, *pandas, matplotlib*) and *R*[8] (with additional libraries *tidyverse*, *arrow, grid*, *broom*, *MMWRweek*). The analysis code is freely available at https://github.com/midas-network/covid19-scenario-hub_r19_manuscript.

6. Supplementary references

1 flepiMoP: The evolution of a flexible infectious disease modeling pipeline during the COVID-19 pandemic. *Epidemics* 2024; **47**: 100753.

2 Davis JT, Chinazzi M, Perra N, *et al.* Cryptic transmission of SARS-CoV-2 and the first COVID-19 wave. *Nature* 2021; **600**: 127–32.

3 Larsen S. Codes for UIUC-IMMCYC v1. DOI:10.5281/zenodo.17575727.

medRxiv preprint doi: https://doi.org/10.64898/2026.04.07.26349269; this version posted April 8, 2026. The copyright holder for this preprint (which was not certified by peer review) is the author/funder, who has granted medRxiv a license to display the preprint in perpetuity.
It is made available under a CC-BY 4.0 International license .

4 Leveraging advances in data-driven deep learning methods for hybrid epidemic modeling. *Epidemics* 2024; **48**: 100782.

5 Projecting Omicron scenarios in the US while tracking population-level immunity. *Epidemics* 2024; **46**: 100746.

6 Data-driven mechanistic framework with stratified immunity and effective transmissibility for COVID-19 scenario projections. *Epidemics* 2024; **47**: 100761.

7 Van Rossum G, Drake FL Jr. The Python Language Reference Manual. Network Theory., 2011.

8 Website. R Core Team (2025). R: A Language and Environment for Statistical Computing. R Foundation for Statistical Computing, Vienna, Austria. https://www.R-project.org/.
